# Psychometric validation of a 7C-model of antecedents of vaccine acceptance among healthcare workers, parents and adolescents in France

**DOI:** 10.1038/s41598-023-46864-9

**Published:** 2023-11-14

**Authors:** Damien Oudin Doglioni, Amandine Gagneux-Brunon, Aurélie Gauchet, Sebastien Bruel, Cyril Olivier, Gérard Pellissier, Nathalie Thilly, Jonathan Sicsic, Jocelyn Raude, Judith E. Mueller

**Affiliations:** 1 Emerging Disease Epidemiology Unit, Institut Pasteur, Université Paris Cité, 75015 Paris, France; 2 Laboratoire Interuniversitaire de Psychologie/Personnalité, Cognition, Changement Social (LIP/PC2S), Univ. Grenoble Alpes, Univ. Savoie Mont-Blanc, 38000 Grenoble, France; 3 CHU de Saint-Étienne – Service d’infectiologie, Saint-Étienne, France; 4 Centre International de Recherche en Infectiologie, Team GIMAP, Inserm, U1111, CNRS, UMR530, Université de Lyon, Université Jean Monnet, Université Claude Bernard Lyon 1, Saint-Étienne, France; 5 Laboratoire Interuniversitaire de Psychologie/Personnalité, Cognition, Changement Social (LIP/PC2S), Univ. Savoie Mont-Blanc, Univ. Grenoble Alpes, 38000 Grenoble, France; 6 Department of General Practice, Faculté de Médecine Jacques Lisfranc, Université Jean Monnet, Université de Lyon, Saint-Étienne, France; 7 Health, Systemic, Process UR 4129 Research Unit, University Claude Bernard, University of Lyon, Lyon, France; 8 GERES (Groupe d’Étude sur le Risque d’Exposition des Soignants), UFR de Médecine Bichat, Paris, France; 9 APEMAC, Université de Lorraine, 54000 Nancy, France; 10 Département Méthodologie, Promotion, Investigation, CHRU-Nancy, Université de Lorraine, 54000 Nancy, France; 11 LIRAES, Université Paris Cité, 75006 Paris, France; 12 EHESP, CNRS, Inserm, Arènes - UMR 6051, RSMS (Recherche sur les Services et Management en Santé) - U 1309, Université de Rennes, 35000 Rennes, France

**Keywords:** Human behaviour, Vaccines, Preventive medicine, Health policy

## Abstract

Support for vaccine decision-making requires a tailored approach taking into account psychological antecedents of vaccine acceptance. We aimed at validating an extended 7C-model of antecedents in three different target population groups (healthcare workers [n = 3870], parents [n = 2002] and adolescents [n = 7118]) and two vaccinations (COVID-19, HPV) in France. We performed a secondary analysis of questionnaires collecting sociodemographic characteristics, attitudes and knowledge on vaccination, and vaccine status and intention. We used standard psychometric techniques to validate a first and second order latent structure, and evaluated their association with vaccine intentionality in three levels (refusal, indecision, acceptance). In all populations, the 7C-model yielded a very good model fit (CFI and TLI > 0.90) and, in comparison with non-nested and nested 5C-models, significantly improved the model performance (Ω^2^, *p* < 0.05; Wald’s test, *p* < 0.05). The resulting vaccine readiness score was strongly associated with vaccine intentionality (acceptance vs. indecision: β_HCW_ = 2.93, β_Parents_ = 2.41, β_Adolescents_ = 1.34; refusal vs. indecision: β_HCW_ = − 1.68, β_Parents_ = − 0.16, β_Adolescents_ = − 0.89.). The addition of confidence in the system and social conformism among antecedents of vaccine acceptance allowed a finer understanding of the continuum moving from refusal to indecision and acceptance. To work with these antecedents in interventional research, appropriate questionnaire items should be developed for various vaccines and target populations.

To foster the understanding of factors that cause doubt, delay or refusal of recommended vaccines, the World Health Organisation defined in 2015 three ‘determinants’^1^ of vaccination:* Confidence* aggregated trust in the effectiveness and safety of a vaccine, as well as in the system that recommends vaccines and makes them available. *Complacency* combining the perception of the risk of contracting a disease and the perception of its threat or negative impact on health. *Convenience* grouped the perceived barriers to vaccine access, such as physical availability, affordability, geographical accessibility, etc. ^1^. Since this first definition, Betsch et al. have extended the model to reach the ‘5C-antecedents of vaccination’^2^ by adding two dimensions*: Calculation* encompassed benefit-risk considerations about vaccination, and *Collective responsibility* considered individuals’ interest in the protection of others by vaccination – be it close contacts or the wider collective group. The 5C-model received a concurrent, a construct, a convergent, and a discriminant validation^3^ and could be considered as a validated descriptive model. This semantic change proposed by Betsch et al., from ‘determinant’ to ‘antecedent’, highlights the contribution of the psychological sciences in understanding the phenomenon of vaccine hesitancy. Antecedents are psychological factors existing prior to the decision to vaccinate and which have an influence on the vaccination decision process^4^.

Results from more recent studies suggested the need for two additional antecedents. First, several authors argued that confidence in the vaccine may be distinguished from confidence in the system^5–7^. In particular during the roll-out of COVID-19 vaccination, several studies found that various proxy items of confidence in the system were predictive factors of intention to get vaccinated against COVID-19^8,9^.la Low confidence in the system could be assessed by expressed distrust of ‘official’ discourse, endorsement of conspiracy affirmations, opposition to governmental decisions or persons, extreme wing voting, etc.^6,10–12^. Second, conformism with the social group has appeared as a factor influencing vaccine decision. Heuristics are commonly used by individuals for complex decisions and have been called ‘bandwaggoning’ in the context of vaccine acceptance^13^. The association of social conformism behaviour with vaccine acceptance or refusal is difficult to show in standard surveys, while discrete choice experiments including the contextual description of vaccine uptake suggested a major impact on theoretical acceptance^14–16^. This descriptive social norm can be distinguished from the injunctive social norm (compliance), included by Geiger^10^ in a recent 7C-model. Geiger et al*.* also coined the term *readiness*, which we use in the following instead of *hesitancy* to emphasise the continuum of attitudes.

Although several studies showed that the two additional attitude domains were associated with vaccine intention or vaccination status^11^, it remains to be demonstrated that they refer to a common concept together with the previously demonstrated five antecedents of vaccination, which we here refer to as vaccine readiness (VR). Accordingly, the primary objective of the present report is to assess whether *Confidence in the system* and S*ocial conformism* can be integrated into the psychological model of antecedents of vaccination and whether they improve its predicting power. The second objective was to evaluate the association of each antecedent domain with *vaccine intentionality* at three levels (refusal, indecision and acceptance). Finally, because various expressions of trust in the system have been described in the literature, we also explore alternative items that may represent the *Confidence in the system* domain.

The concept of psychological antecedents of vaccine hesitancy or readiness implies to some extent a claim of universal validity on most if not all vaccines and vaccinations. This is illustrated by the evaluation of the 5C model published by Betsch et al., which explored associations of the 5C domains with three different vaccines (MMR, HPV, influenza) and respective study populations. In consequence, we included in the present evaluation two recommended vaccinations (COVID-19 and human papilloma virus vaccines [HPV]) and three population groups involved in these two vaccinations (healthcare workers, parents of middle school pupils aged 11–15 years, and 13–15-year-old girls and boys). While evidence available on effectiveness and safety of HPV and Covid-19 vaccines differed at the time of the data collection, with Covid-19 being a new vaccine for epidemic response, these two vaccines both were recommended, but not mandatory, and recommended primarily to the evaluated group.

## Materiel and methods

### Data collection

The three datasets were collected through anonymous online surveys in adherence to national and international standards of research ethics (French law, Helsinki declaration) and data protection (GDPR). The study protocol on COVID-19 (dataset 1: HCW-Covid-19) was approved by the Institutional Review Board “Terre d’Éthique” of Centre Hospitalier Universitaire Saint-Etienne (N° IRBN1092021/CHUSTE), the study on HPV (dataset 2: Parents-HPV and dataset 3: Adolescents-HPV) was approved by the French Ethics Committee ‘Comité de Protection des Personnes Sud-Est VI’ (ID-RCB: 2020-A02031-38). Invited individuals had to check “I agree to participate” after reading the study information to start the online survey. Due to the anonymous nature of the data collection, no formal informed consent could be collected.

For the first database (dataset 1: HCW-Covid-19), data were collected between December 18, 2020, and February 1, 2021, among healthcare and welfare sector workers in France, at the beginning of the COVID-19 vaccine roll-out^11^. As previously reported, the Research Group for the Prevention of Occupational Infections in Healthcare Workers (GERES) published an online survey via the Sphinx online survey platform that was disseminated by email chain referral throughout France, including overseas departments. At study start, vaccine efficacy data had been published or announced by AstraZeneca, Pfizer and Moderna. During the data collection, vaccination became recommended for nursing homes residents, ≥ 50-year-old HCWs and eventually ≥ 75-year-old persons. During the entire study period, a curfew, but no travel or work restrictions were imposed. No controversy had yet emerged about these vaccines and vaccination strategy, although recurrent concerns were the unusual speed of vaccine development. This data set has served for an epidemiological analysis of knowledge and attitudes with the objective to explore the psychological antecedents as determinants of COVID-19 vaccine acceptance, in particular their extension from the 5C- to a 7C-model including *Social conformism* and *Confidence in the system*^11^. However, we had not yet conducted a psychometric analysis to demonstrate the validity of the theoretical model. From the initially 5234 participants, we excluded non-healthcare professionals (e.g., welfare system workers), leaving 3870 participants in five professional categories: nurses, nurse assistants, medical doctors, biomedical professionals (midwives, pharmacists, biologists) and other healthcare workers (paramedical and non-medical staff in direct physical contact with patients) (Table 1).

**Table 1:** Sociodemographic characteristics of participants in the three datasets.

Profession	N total	Female		Age (years)					
				18–34		35–49		50 and more	
		N	%^h^	N	%^h^	N	%^h^	N	%^h^
**Total healthcare workers (COVID-19)**	3870	3007	77.7	949	24.5	1547	40.0	1374	35.5
Other HCW^a^	819	696	85.0	247	30.2	341	41.6	231	28.2
Nurse assistants	491	444	90.4	134	27.3	210	42.8	147	29.9
Nurses	1197	1019	85.1	296	24.7	518	43.3	383	32.0
Biomedical professionals^b^	487	330	67.8	91	18.7	203	41.7	193	39.6
Medical doctors	876	518	59.1	181	20.7	275	31.4	420	47.9
**Total parents (human papilloma virus)**	2002	1798	89.8	119	5.9	1211	60.5	673	33.6
Independent^c^	106	95	89.6	8	7.5	65	61.3	33	31.1
Executive^d^	423	349	82.5	4	0.9	250	59.1	169	40.0
Technician^e^	444	408	91.9	8	1.8	288	64.9	148	33.3
Employee^f^	604	585	96.9	36	6.0	393	65.1	175	29.0
Worker^g^	80	55	68.8	10	12.5	44	55.0	26	32.5
Other	345	306	88.7	53	15.4	170	49.3	122	35.4
**Total adolescents (human papilloma virus)**	7118	3813	53.6	NA	NA	NA	NA	NA	NA
4th grade (college)	3813	1926	50.5	NA	NA	NA	NA	NA	NA
3rd grade (college)	3305	1887	57.1	NA	NA	NA	NA	NA	NA

^a^For example radio manipulator, laboratory technician, psychologist, other scientific support functions. ^b^Include pharmacist, midwife, biologist, dentist. ^c^For example farmer, company manager, craftsman. ^d^For example executive, doctor, engineer. ^e^For example technician, nurse, teacher. ^f^For example waiter, casher, secretary. ^g^ For example plumber, electrician, hairdresser.

^h^Row percentage.

The other two datasets were part of a cluster-randomised controlled trial evaluating the effectiveness, efficiency and implementation of three intervention components to increase HPV vaccine uptake in 61 French middle schools^17^. Data were collected between 22 November 2021 and 8 February 2022, through an internet-based survey. Parents of middle school pupils (French classes 6th to 3rd grade) were invited to study participation via an email sent by their child’s school (dataset2: Parents-HPV). Parents received information on the project in the same email and had the opportunity to oppose to their child’s participation in the survey. If more than one child attended the school, parents were asked to complete the questionnaire with their oldest child in mind. Middle school pupils (French classes of 4th and 3rd grade, typically aged 13–15 years) completed the questionnaire during in-class sessions (dataset 3: Adolescents-HPV). The datasets include 2002 adults divided into six groups according to their current or last professional activity (for example, independent, executive level, blue-collar worker) and 7118 adolescents in two grade levels (4th and 3rd grade) (Table 1).

#### Evaluation of the psychological antecedents

The questionnaires on 7C antecedents in the three studies were developed by the same research team. HPV-related questionnaires were elaborated for parents and adolescents in the randomised controlled trial on HPV vaccine promotion (protocol approved by the French Ethics Committee in mid-2020). This questionnaire then served as model for the HCW questionnaire that was adapted for data collection at the start of the Covid-19 vaccine roll-out late 2020. This procedure assured coherence between the three questionnaires.

The questionnaire for dataset 1 was developed to explore healthcare workers’ attitudes and knowledge around 7C antecedents (5C plus *Social conformism* and *Confidence in the system*) as determinants of COVID-19 vaccine acceptance.

The questionnaires for dataset 2 (parents-HPV) and 3 (adolescents-HPV) were developed to allow the evaluation of the effect of intervention components on knowledge, attitudes and practices^18^ in relation to the 5C antecedents plus *Social conformism*. Following the observation of the importance of *Confidence in the system* items among healthcare workers, we added items on this attitude domain to the parent and adolescent questionnaires. In addition, we aimed at understanding the different meanings of the items related to *Confidence in the system*. Thus, we assessed the difference between a formulation close to health/vaccination used only in the parents’ questionnaire (Item A, database 2: parents-HPV) and a more distal formulation used with both parents and adolescents (Item B).

The questionnaire items corresponding to the hypothetical antecedents are shown in supplementary material 1.

#### Evaluation of vaccination intentionality

The dataset 1 collected vaccine intentionality through the question ‘If a COVID-19 vaccine was offered to you today, would you get vaccinated?’, with the modalities ‘Yes/No/Do not know’. Answering positively, negatively and ‘do not know’ defined acceptance (n = 2256), refusal (n = 869), and indecision (n = 745), respectively.

The dataset 2 (Parents-HPV) and 3 (Adolescents-HPV) evaluated vaccine awareness, intentionality and uptake following the Prochaska and DiClemente model of behaviour change^19^ adapted to vaccination. The variable comprises six modalities:

1. *Ignorance* of the HPV and its vaccination (‘Have you heard about HPV and HPV vaccination?’).
2. *Precontemplation*: respondents who had heard about HPV and HPV vaccination, but who did not consider it as relevant (‘My child is / I’m not concerned with the vaccination against HPV.’).
3. *Contemplation*: respondents who feel concerned by the HPV vaccination but who are not yet certain of willing to vaccinate their child or to be vaccinated (‘I consider HPV vaccination as relevant for my child/for me, but I am not sure of getting (her/him) vaccinated.’).
4. *Intention*: respondents who wanted to vaccinate/be vaccinated but do not yet have implemented actions (‘I have the intention to get soon an appointment to get (my child) vaccinated.’).
5. *Preparation*: respondents who have already implemented actions but are not yet vaccinated (‘I have an appointment or prescription to get (my child) vaccinated’).
6. *Vaccinated* (‘Is your child/are you vaccinated against the human papilloma virus (HPV)? (Vaccine name: Gardasil™ or Cervarix™.)’)

After excluding participants who are unaware of HPV since they could not make a decision about vaccination (Ignorance; n_Parents_ = 212, n_Adolescents_ = 3336), we grouped into refusal (n_Parents_ = 139, n_Adolescents_ = 1020) those participants who were in the precontemplation phase since they are aware of HPV and its vaccination but did not intent to accept vaccination. We assume that this group would refuse HPV vaccination if proposed, although we did not ask the question directly. Indecision (n_Parents_ = 352, n_Adolescents_ = 967) included participants in the contemplation phase who were still considering whether to be vaccinated against HPV. Finally, acceptance (n_Parents_ = 1276, n_Adolescents_ = 2725) included participants who intended to be vaccinated (intention), were in the preparation phase or were vaccinated.

We also collected sociodemographic data, such as age, gender, or professional category.

### Statistical analyses

#### Extension from five to seven antecedents: factor analysis

The main objective of the factor analysis was to confirm a theoretical model of seven antecedents of vaccination that refers to a common latent factor, vaccine readiness (VR). In consequence, we explored the 7C-model’s internal consistency and determined the relative performance of the 7C- versus the 5C-model in predicting VR.

The seven psychological antecedents of vaccination are latent variables, named factors, i.e. unobservable variables assessed from observed variables, named indicators. Similarly, VR, in itself, is also a latent variable assessed from other latent variables (the seven antecedents). Thus, antecedents are first order latent variables while VR is a second order latent variable.

##### Confirmatory factor analysis (CFA)

To address the first objective of this study, we conducted a CFA to validate, in the three datasets, the existence of a first-order latent factorial organisation in seven factors corresponding to the seven psychological antecedents of vaccination. Such organisation requires a covariance relationship that indicates the possible existence of a common formative second order factor, postulated to be VR. Then, we conducted a second CFA on the first order latent factors grouped in a single factor to confirm the existence of second order latent structure. As assessing the statistical significance of the loading of each indicator is important, we fixed the variance of the latent factor^20,21^.

We used the two-index presentation strategy^22^ including the maximum likelihood (ML)‐based standardised root mean squared residual (SRMR) with a cut-off value under 0.08^22^; and, the root mean square error of approximation (RMSEA) with 90% confidence intervals and cut-off value close to 0.07^23^. We added three indices of goodness of fit^24^: the Wheaton et al*.*’s relative/normed chi square (χ2/dF) with a range from 2 to 5^25^; the comparative fit index (CFI) with a value greater than 0.90^22^; and, the non-normed fit index also known as the Tucker-Lewis index (TLI) with a 0.90 threshold^24^.

For the first order latent factors, if the CFA models were a poor fit to the data, we aimed at improving the fit in two successive steps:

1. We removed from the model indicators with low factor loadings (< 0.30), while keeping at least one indicator per factor.
2. We added error covariance between indicator pairs as suggested by modification indices (Residual Covariance Modification Indices produced by Lavaan ^26^) beginning with the highest.

In case of model modification, we used Akaike Information Criterion (AIC) to compare the non-nested models^20^, favouring lower AIC values.

To estimate the factor score of the first and second order latent factors, we used the regression or exact method with Bartlett’s correction for bias in factor means^27^ by using the lavPredict function included in the Lavaan library^26^. The resulting scores had a group mean close to zero and a free standard deviation.

We compared the resulting mean scores of the seven psychological antecedents of vaccination and of VR between subgroups of the three populations: professional category (HCW, parents) and school grade (adolescents). Vaccine intention varies substantially between the subgroups, observed in the present data bases and in the literature^28^.

##### Internal consistency

Internal consistency represents the extent to which items are closely related, i.e. the extent to which they measure the same dimension of a latent variable. We assessed internal consistency using McDonald’s Omega (ώ), which is based on a common factor analysis model and is more accurate than the Cronbach’s alpha^29,30^. Evaluation was performed on the first- and second-order latent factors. ώ is interpreted in the same way as α: the closer the value is to one, the more consistent the model is considered to be. Thus, a value equal to or greater than 0.70 indicates satisfactory consistency^31^.

##### Comparative performance analysis

We consecutively used two steps to evaluate the performance of the extended 7C-model compared to the 5C-model:

1. First, following the same steps as for the definition of the extended 7C-model, we elaborated a concurrent non-nested 5C-model. This concurrent model was compared to the 7C-model using Vuong’s test for non-nested model^32^ which consists of two subtests: first, a test of distinguishability, indicating whether or not the models can possibly be distinguished on the basis of the observed data; second, the robust likelihood test, indicating whether or not each model fits better than the reference model (i.e. the 5C-model).
2. Second, based on the extended model with seven antecedents, we defined three nested models by subtraction of *Social conformism* (6C.1-model), *Confidence in the system* (6C.2-model) and both new antecedents (5C-model). Then we performed two Wald’s tests (5C–6C.1–7C/5C–6C.2–7C). A performance score, included in the Performance library^33^, was added to rank these models.

#### Influence of the seven antecedents on vaccine intentionality: regression analysis

We performed multinomial logistic regressions (MLR) with vaccine intentionality (refusal, indecision, acceptance) as dependent variable. Modality ‘indecision’ served as reference category, since we aimed at understanding which determinants, among people uncertain about their choice of vaccination, encouraged them to be vaccinated or, on the contrary, hindered vaccination. The seven psychological antecedents of vaccination served as independent variables. An alternative model included the vaccine readiness factorial score (second order latent variable) as the independent variable. The MLR was constructed in two blocks of variable: (i) the five known antecedents, i.e. the 5C-model and (ii) the two additional antecedents in order to assess the plus-value of adding these two antecedents. All analyses were conducted separately in each dataset, without performing any comparison between the three populations.

We performed crude analyses, as we aimed at assessing the overall predicting power of the seven psychological antecedents of VR on the vaccination intentionality. Result of additional analyses adjusting for sociodemographic data (age, gender, and professional category for HCW and parents, and gender, and school grades for adolescents) are available in supplementary material (2 for HCW, 3 for parents, and 4 for adolescents). All statistical analyses, including scripts and extended results, are available as supplementary material (2 for HCW, 3 for parents, and 4 for adolescents).

Statistical analyses were conducted using Jamovi software^34^, version 2.3 or R Studio, version 3.6, both run under R^35^, version 4.1. For confirmatory factor analysis (CFA), we used the package Lavaan^26^. For principal component analysis and internal consistency, we used the Psych library^36^. Comparisons of performance were made with package Performance^33^. Comparison and regression were performed using the Car libraries^37,38^, the Emmeans library^39^ and the Nnet library^40^.

## Results

### Extension from five to seven psychological antecedents

#### Confirmatory factor analysis (CFA)

In all three study databases, the confirmatory analyses suggested a factorial structure with seven first order latent factors (Table 2A) and a second order latent factor (Table 2B). Although all the seven first order factors participated in the definition of the second order latent factor, some of them participate poorly in the definition of VR: for HCW-Covid-19 *Convenience* (λ = 0.269), for Parents-HPV and Adolescents-HPV, *Confidence in the system* (respectively, λ_item B_ = 0.291 and λ_item A_ = 0.145).

**Table 2:** Summary of results from confirmatory factor analyses.

Model	CFI	TLI	SRMR	RMSEA			Chi^2^/dF	AIC
					90% CI			
	> 0.90	> 0.90	< 0.08	< 0.07	Lower	Upper	< 5	
**A:** **Result synthesis of CFA on first order latent variables**								
*Healthcare workers (COVID-19)*								
Initial #1	0.825	0.801	0.049	0.049	0.048	0.050	13.508	423,174
#2	0.902	0.876	0.044	0.055	0.053	0.057	16.710	275,468
#3	0.928	0.908	0.036	0.047	0.045	0.049	12.670	274,837
** Final**	0.934	0.912	0.035	0.050	0.048	0.052	14.020	246,244
* Parents (HPV)*								
Initial #1	0.831	0.790	0.056	0.067	0.064	0.070	9.917	99,312
#2	0.923	0.872	0.045	0.077	0.072	0.083	12.790	59,583
**Final**	0.980	0.963	0.025	0.045	0.038	0.051	4.937	54,306
*Adolescents (HPV)*								
Initial #1	0.826	0.764	0.050	0.059	0.057	0.061	25.560	225,430
#2	0.834	0.753	0.056	0.072	0.069	0.074	37.820	185,437
**Final**	0.958	0.914	0.031	0.049	0.045	0.053	18.140	145,204
**B:** **Result synthesis of CFA on second order latent variables**								
HCW	0.980	0.968	0.023	0.063	0.056	0.069	21.485	105,589
Parents	0.982	0.974	0.021	0.055	0.045	0.066	6.988	33,746
Adolescents	0.974	0.961	0.023	0.046	0.041	0.051	16.064	98,269

*CFI:* comparative fit index. *TLI:* Tucker-Lewis Index. *SRMR:* maximum likelihood (ML)‐based standardised root mean squared residual. *RMSEA:* root mean square error of approximation. *Chi*^2^*/dF:* Wheaton et al.’s relative/normed chi square. *AIC:* Akaike Information Criterion.

In Parents-HPV, we tested the differentiated saturation of two formulations that may represent the *Confidence in the system* domain (item A vs. item B; see supplementary material 1). In CFA, the distal formulation (item A, λ_item A_ = 0.101) was less strongly associated with VR than the proximal formulation (item B, λ_item B_ = 0.291). We thus kept item B in further analyses on Parents-HPV.

The contribution of knowledge items to the first-order factors (included through the CFA selection process) varied between databases: eight out of 17 items for HCW-Covid-19, two out of 12 for Parents-HPV and three out of 11 for Adolescents-HPV (supplementary materials 1 to 3).

#### Internal consistency

Internal consistency of the first order latent structure was good in all three study databases (> 0.70) and comprised between ώ = 0.853 (HCW-Covid-19), ώ = 0.851 (Parents-HPV) and ώ = 0.790 (Adolescents-HPV). For the second order latent structure, reliability was good, spanning from ώ = 0.839 (HCW-Covid-19), ώ = 0.840 (Parents-HPV) to ώ = 0.771 (Adolescents-HPV). Using item A or B for *Confidence in the system* for Parent-HPV did not yield different results.

#### Comparative performance analysis

Comparative performance analysis suggested better performance of the 7C- compared to the 5C-model (*p* < 0.001, Table 3). The non-nested analysis using Vuong’s test confirmed that, in all three databases, the 7C-model was distinguishable from the 5C-model and provided a significantly better data fit (*p* < 0.001). Moreover, nested analysis using Wald’s test confirmed that the 7C-model was of significantly better performance than a 6C- or 5C-model (*p* < 0.001).

**Table 3:** Summary of results from comparative performance analysis.

	Non-nested model				Nested model				
	Distinguishability		Robust likelihood test		dF	ΔdF	Wald’s test	*p*-value	Performance score (%)
	Ώ^2^	*p*-value	RL value	*p*-value					
**Healthcare workers (COVID-19)**									
5C-model					5228		(reference)		0.00
6C-model:									
^(1)^ with *Confidence system*					5227	1	5.89e + 08	< 0.001	35.67
^(2)^ with *Social conformism*					5227	1	3.91e + 08	< 0.001	22.78
7C-model	0.71	< 0.001	− 520.35	< 0.001					100
7C versus 6C.1					5226	1	3.09e + 08	< 0.001	–
7C versus 6C.2					5226	1	5.08e + 08	< 0.001	–
**Parents (HPV)**									
5C-model					1769		(reference)		0.00
6C-modelss:									
^(1)^With *Confidence system*					1768	1	7.78e + 29	< 0.001	0.06
^(2)^With *Social conformism*					1768	1	1.15e + 30	< 0.001	68.98
7C-model	0.95	< 0.001	− 82.14	< 0.001					100
7C versus 6C.1					1767	1	1.12e + 30	< 0.001	–
7C versus 6C.2					1767	1	7.53e + 29	< 0.001	–
**Adolescents (HPV)**									
5C-model					4757		(reference)		0.00
6C-models									
^(1)^With *Confidence system*					4756	1	4.45e + 29	< 0.001	51.59
^(2)^With *Social conformism*					4756	1	2.23e + 30	< 0.001	8.97
7C-model	1.75	< 0.001	− 63.61	< 0.001					100
7C versus 6C.1					4755	1	2.07e + 30	< 0.001	–
7C versus 6C.2					4755	1	2.88e + 29	< 0.001	–

*dF*: degree of freedom. *ΔdF:* variation of degree of freedom between subsequent models.

#### Comparison of the distribution of the seven antecedents, vaccine readiness score and vaccine intentionality between population subgroups

Factorial mean scores of the individual antecedents and of VR differed substantially and significantly between the subgroups of the three populations (Table 4). An exception was *Social conformism* for Parents-HPV and Adolescents-HPV, for which no significant difference between professional categories and school grade was observed.

**Table 4:** Distribution of the factorial scores of seven antecedents of vaccine readiness, vaccine readiness score and level of vaccine intentionality in population subgroups.

	Vaccine intentionality				Factorial scores of antecedents and vaccine readiness in population subgroups							
	Refusal	Indecision	Acceptance	*p*-value for difference between groups (χ^2^)	Confidence vaccine	Convenience	Complacency	Calculation	Collective responsibility	Confidence system	Social conformism	Vaccine readiness score
	%	%	%		Means	Means	Means	Means	Means	Means	Means	Means
**French HCW**	22	19.8	58.1		0.000**	0.000**	0.000**	0.000**	0.000**	0.000**	0.000**	0.000**
Other HCW	26.3	23.4	50.3	< 0.001	0.780	0.218	0.625	0.591	0.380	0.358	0.731	− 0.626
Nurse assistant	45.2	24.2	30.6		0.466	0.073	0.410	0.479	0.319	0.246	0.656	− 0.465
Nurse	27.3	23.1	49.6		− 0.130	− 0.078	− 0.159	− 0.169	− 0.109	− 0.315	− 0.382	0.187
Biomedical	10.5	13.1	76.4		− 0.783	− 0.285	− 0.672	− 0.754	− 0.609	− 0.567	− 0.682	0.768
Medical doctors	6.2	10.7	83.1		− 0.311	− 0.043	− 0.327	− 0.193	− 0.115	− 0.030	− 0.055	0.200
**Adults**	7.9	19.9	72.2		0.004**	0.006**	0.008**	0.002**	− 0.001**	0.000**	0.003^**NS**^	0.004**−
Independent	25.5	12.7	61.8	< 0.001	0.016	− 0.038	0.036	0.034	− 0.060	− 0.040	0.020	0.004
Executive	10.4	14.9	74.6		0.194	0.233	0.290	0.439	0.328	0.191	0.042	− 0.321
Technician	9.3	18.6	72.1		0.103	0.138	0.175	0.228	0.213	0.055	0.010	− 0.183
Employee	18.6	20.9	60.5		− 0.080	− 0.061	− 0.156	− 0.238	− 0.140	− 0.085	0.024	0.147
Workers	27.8	26.6	45.6		− 0.227	− 0.461	− 0.552	− 0.522	− 0.849	− 0.121	− 0.194	0.568
Other	31.4	14.2	54.3		− 0.209	− 0.282	− 0.222	− 0.403	− 0.327	− 0.118	− 0.067	0.303
**Adolescents**	21.6	20.5	57.8		0.004*	0.000*	− 0.005*	0.003**	0.003**	0.000**	0.004^**NS**^	0.011**
4th grade	49.9	13.8	36.4	< 0.001	− 0.028	− 0.047	− 0.068	− 0.047	− 0.047	0.069	− 0.005	− 0.049
3rd grade	44.7	13.7	41.6		0.038	0.049	0.063	0.057	0.058	− 0.080	0.014	0.076

Asterisks indicate *P*-values for difference between population subgroups: ^NS^ non-significant; * < 0.05; ** < 0.001. For further details and post-hoc analysis, see supplementary material.

**Figure 1:**
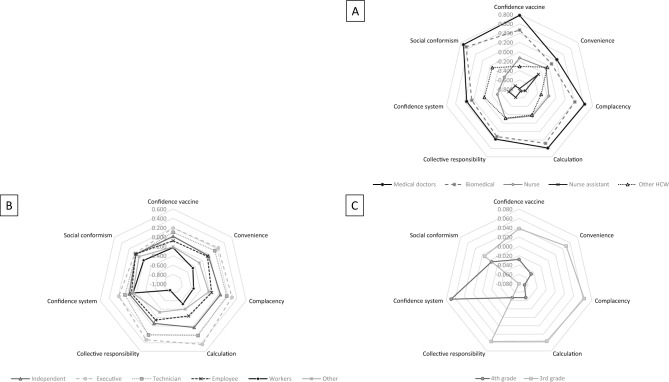
Kiviat diagrams of the mean level of the seven antecedents of vaccine readiness in the three databases (France, 2020–2021). (**A**) Healthcare workers (HCW) – COVID-19 (n = 3870). (**B**) Parents – HPV (n = 1791). (**C**) Adolescents – HPV (n = 4802). (*Supplemental 5 provides further details*.)

In the three databases, the vaccine readiness score was lower among nurse assistants, workers of the parent-HPV population and 4th graders; and greater in medical doctors (HCW-Covid-19), executive professionals (Parents-HPV) and 3rd graders. This gradient was observed for all antecedents, except for adolescents, were 4th graders expressed more *Confidence in the system* (‘School responds to my needs’) than 3rd graders (Fig. 1).

Frequency of the vaccine intentionality levels differed significantly between all population subgroups (Table 4) with higher frequency of acceptance among medical doctors and other biomedical professionals, executive and technician categories, and 3rd (vs. 4th) grade.

### Association between the seven antecedents, the vaccine readiness score and vaccine acceptance

The addition of the antecedents *Confidence in the system* and *Social conformism* improved significantly the fit of models evaluating the association between antecedents and vaccine intentionality (Table 5). The Nagelkerke pseudo-R squared (for the models including the seven psychological antecedents) was R^2^_N_ = 0.571 (HCW-Covid-19), R^2^_N_ = 0.449 (Parents-HPV) and R^2^_N_ = 0.339 (Adolescents-HPV).

**Table 5:** Model fit for prediction of vaccine intentionality (with reference: indecision against acceptance or refusal).

Population	Model	R^2^N	Overall model test			Δχ^2^	ΔdF	*p*-value^a^
			*χ*^2^	*dF*	*p-value*			
**Seven antecedents of vaccine readiness**								
HCW-Covid-19	5C	0.542	4688	10	< 0.001			
	7C	0.571	4985	14	< 0.001	297	4	< 0.001
Parents-HPV	5C	0.442	1020	10	< 0.001			
	7C	0.449	1037	14	< 0.001	17	4	0.002
Adolescents-HPV	5C	0.331	2433	10	< 0.001			
	7C	0.339	2493	14	< 0.001	60	4	< 0.001
**Vaccine readiness score alone**								
HCW-Covid-19	VR	0.556	4824	2	< 0.001			
Parents-HPV	VR	0.434	999.4	2	< 0.001			
Adolescents-HPV	VR	0.315	2301	2	< 0.001			

Models were unadjusted to reflect only the influence of the seven antecedents of vaccine readiness and of vaccine readiness.

^a^*p*-value obtain after Wald’s test.

*R*^2^*N*: Nagelkerke pseudo-R-squared.* Δχ*^2^: variation of chi squared between subsequent models. *ΔdF:* variation of degree of freedom between subsequent models.

**Figure 2:**
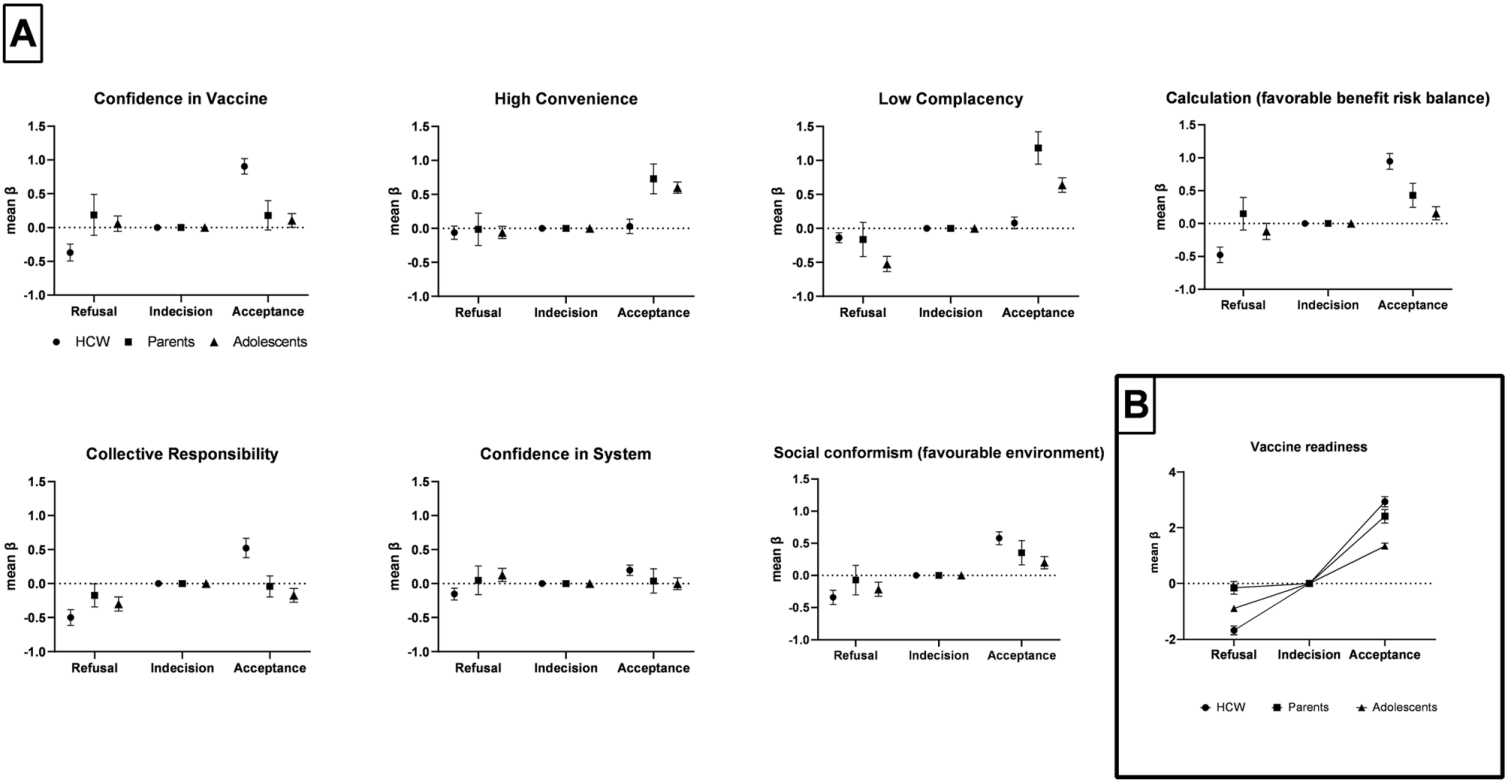
Association between antecedents of vaccine readiness and vaccine intentionality. Healthcare workers (HCW) – COVID-19 (n = 3870), Parents – HPV (n = 1791), Adolescents – HPV (n = 4802) in France, 2020–2021. (**A**) Including the seven antecedents of vaccine readiness. (**B**) Using the factorial score of vaccine readiness. (*Coefficients estimated in multinomial logistic regression models unadjusted for sociodemographic data*.)

In all three populations, higher levels of the vaccine readiness score were significantly associated with acceptance (vs. indecision) and lower scores with refusal (vs. indecision) (Fig. 2B). The latter association was strong for HCW-Covid-19 (β = 2.93) and Parents-HPV (β = 2.41) but weaker for Adolescents-HPV (β = 1.34). This pattern was more distinctive for individual antecedents (Fig. 2A).

For HCW-Covid-19, refusal (vs. indecision) was mostly predicted by low *Collective responsibility* (β = − 0.49), low *Confidence in vaccine* (β = − 0.37) low *Calculation* (benefice-risk balance, β = − 0.34), and low *Social conformism* (sceptical environment, β = − 0.34), and to a smaller degree by *Confidence in the system* (β = − 0.15). Acceptance (vs. indecision) was predicted by *Calculation* (high benefice-risk balance, β = 0.95), *Confidence in vaccine* (β = 0.91), high *Social conformism* (favourable environment, β = 0.58), *Collective responsibility* (β = 0.52) and to a smaller degree by *Confidence in the system* (β = 0.20). *Convenience* was not associated with any level of intentionality.

For Parents-HPV, refusal was only predicted by low *Collective responsibility* (β = − 0.17); while acceptance was predicted by low *Complacency* (β = 1.18), high *Convenience* (β = 0.73), high *Calculation* (benefit-risk balance, β = 0.43) and high *Social Conformism* (favourable environment, β = 0.35).

For Adolescents-HPV, refusal was mainly predicted by high *Complacency* (β = − 0.52) and low *Collective responsibility* (β = − 0.30), but also *Social Conformism* (β = − 0.21) and *Calculation* (β = − 0.12). Notably, those who agreed with the statement: ‘the school system meets my needs’ were more prone to refuse (*Confidence in the system*, β = 0.13). Acceptance was strongly associated with all antecedents except for *Confidence in the system*, but mostly with low *Complacency* (β = 0.64) and a high *Convenience* (β = 0.60).

## Discussion

In this psychometric study reanalysing separately three distinct databases from the French population, the expansion of the 5C-model of antecedents by *Social conformism* and *Confidence in the system*, significantly improved the quality of the model, with a second order latent factor representing the global attitude towards the given vaccination (vaccine readiness). The *Confidence* dimensions of the 5C-model was split into two dimensions, one referring specifically to the confidence in vaccines, i.e. safety and efficacy (*Confidence in vaccine*) and the other referring to confidence in the system that recommends vaccines and makes them available (*Confidence in the system*). We highlighted differences in the level of each antecedent of vaccination between population subgroups (with professions for HCW and parents, or level of study for adolescents used as proxies for different levels of health literacy). Moreover, the seven individual antecedents of VR and the VR score were closely associated with vaccine intentionality. Assessing *Confidence in the system* and *Social conformism* separately as two antecedents of VR performed better to define VR and added explanatory power to understand the impact of VR on vaccination acceptance and behaviour.

Some items used to define 7C antecedents and intentionality varied between the three study populations, and these variations represent necessary adaptations of the questionnaire to the vaccine and the population. As we explored whether the 7C antecedents can help understand vaccine acceptance within each of the three populations, the specific differences in item wordings between the three study populations do not substantially impact the validity of analyses. The analyses do not aim at comparing levels of intentionality or vaccine readiness between the three populations, which should not be attempted, due to the differences in item formulations and as the sampling procedures did not aim at optimising representativity. Furthermore, despite differences, the theoretical model fits the data from three independent populations, which we interpret as proof of robustness.

*Confidence in the system* was assessed differently in the three populations and vaccinations. In HCW, it was assessed by two attitudinal questions, a first item on the perception of an incentive from the employer on the intention to get vaccinated and a second item on the confidence in the authorities to manage the health and economic crisis related to COVID-19. The former represented the capacity of reactance against vaccine promotion by an entity with potentially conflictual relationship; and the second the general confidence in an overriding authority which included the negative emotions felt by the respondent on this authority. It is of note that by the time of the questionnaire, at the start of the vaccine rollout in France, confidence in the authorities did not yet include vaccine-related aspects, but was dominated by decisions related to non-pharmaceutical measures (personal communication, based on verbatim collected in the PrefMeCo study^41^). Both items referred to health and vaccination and had a strong load to their latent factor. Consequently, *Confidence in the system* had a strong association with the second-order latent factor and influence HCW’s intentionality.

For HCW-Covid-19, both proximal and distal formulations of *Confidence in the system* were retained by the CFA as first-order factors and the resulting second-order factor (7C-domain) contributed significantly to the prediction of vaccine intentionality. By contrast, in Parents-HPV and Adolescents-HPV, the corresponding items were less integrated into the factors, possibly due to the chosen formulations. In parents, *Confidence in the system* was assessed by two items questions: general agreement with school-based vaccination as a proximal item and attitude that the school meets the child’s needs as a distal item. The proximal item, however not specific to HPV, had a higher albeit weak association with the second-order latent factor compared to the distal item. Despite this association, the proximal item had no influence on parental intentionality. An explanation might be that parents intending vaccination have more confidence in their family physician than in school-based health services. In adolescents, the same distal item was poorly associated with the second order latent variable and consequently was not associated with vaccine intentionality, either acceptance or refusal (vs. indecision). Overall, we suggest that the formulation of the items on *Confidence in the system* should be further refined to better capture the underlying concept. This concept should be framed between the confidence given to vaccine recommendations and people who deliver them on one extreme (which is part of the antecedent *Confidence in vaccines*) and adherence to conspiracy on the other (as suggested by Geiger et al*.* for a seventh antecedent^10^). The presented results can guide towards such improved formulations, possibly referring to reactance against vaccine promotion^42,43^.

In our proposed model, *Social conformism* focuses on the influence of trusted person, which rather refers to emotions and heuristics, i.e. descriptive social norms, in contrast to Geiger et al*.* who focused on compliance with monitoring and sanctions, which represents agreement with injunctive social norms. We found in all three study groups moderate to strong associations between this antecedent and vaccine readiness and with intentionality, meaning that vaccination attitude, and consequently intentionality and behaviour, is socially contextualised and influenced by trusted persons. This provides a strong argument for tailored and community-level interventions.

It is of particular interest that among parents and regarding HPV, acceptance (vs. indecision) was strongly associated with several antecedents, while refusal (vs. indecision) was associated with only a few antecedents, and this less strongly. This observation could in part be due to the less sharp distinction we made between strict refusal and the state of pre-contemplation in the studies on HPV, but this pattern was also observed in the HCW-Covid-19 study, where refusal corresponded to absence of intention. Another explanation may lie in the rather favourable attitudes towards vaccination of our populations. This pattern suggests that antecedents of vaccine readiness will be useful for making individual move from indecision to acceptance, for example during motivational interviewing. An appropriate technique but could consist of supporting indecision with positive antecedents, instead of correction of wrong convictions and negative attitudes.

Within all three databases, we found statistically significant differences in the level of the seven antecedents of vaccine readiness between individual categories (HCW and parents) and school grades (adolescents). For example, the group of parents who were workers stood out with particular low levels of perception of collective responsibility and accessibility, and with high complacency around HPV vaccination. Further research should explore the role that specific 7C antecedents play in such differences between population subgroups, as such finer understanding could help developing targeted interventions to improve vaccine acceptance.

The proportion of knowledge and attitude items was different in the three databases. In HCW, knowledge items account for almost half of the items kept after CFA which could depict the influence of their biomedical background^44^. However, in the general population (parents and adolescents), these items were marginal suggesting that interventions might primarily focus on attitudes. Further studies are needed to disentangle the influence of knowledge on attitudes and on vaccine intention.

One originality of our analysis of vaccine intentionality lies in that we differentiated between refusal and indecision, instead of regrouping them into absence of intention, as usually done by studies. As discussed above, our results suggest that psychological antecedents have different importance depending on whether one looks at the difference between refusal and indecision or between indecision and acceptance. To quit refusal, the perception of the collective benefit of vaccination (*Collective responsibility*), a low *Complacency* and a vaccine-favourable environment (*Social conformism*) were prominent in all study populations and vaccinations. To reach acceptance, the prominent antecedents differed between groups: a complex mix for HCW-Covid-19, but mainly *Convenience* and *Complacency* for HPV vaccination. Previous studies have usually analysed intention versus indecision/refusal, typically yielding stronger effect estimates and gaining statistical power. However, given our results, it appears that a three-level approach for understanding of vaccine acceptance and development of interventions should be preferred.

Our study has several limitations. First, the information is based on stated intention without information on actual uptake (COVID-19); or only self-reported uptake (HPV). There is a considerable gap between intention and behaviour^45^ and future studies may need to work on validated vaccine uptake information. Second, the term ‘antecedent’ implies a prospective association, which requires prospective study design, to assure that attitudes were present before the vaccine decision. Our cross-sectional data therefore cannot conclude with certainty in the direction of association. Third, compared to the results found with HCW, parents showed less internal coherence in terms of the 7C-model structure (McDonald’s Omega). This may reflect the greater diversity of the general adult population than HCW population. As our study samples were not established to be representative of the population group, interpretation should be made with caution and results may not be generalisable to the entire French population.

## Conclusion

Our results suggest that it is appropriate to include *Confidence in the system* and *Social conformism* as psychological antecedents of vaccination. We found that the seven antecedents capture differences between population subgroups that have difference levels of vaccine intention and acceptance. These results can guide towards a better understanding of barriers and drivers of vaccination behaviour. Further work is needed to refine the antecedent of *confidence in the system*, between confidence, reactance against vaccine promotion, and conspiracy.

This paper highlights the value of studying psychological antecedents to understand the barriers to and the levers for improved vaccine acceptance. The extended model of seven antecedents of vaccine readiness is a diagnostic tool that allows detailed comprehension of drivers of vaccine acceptance and may support tailoring intervention as suggested by the WHO^46,47^. In addition, the 7C-model could be used during motivational interviewing to explore the individual situation in the vaccine indecision process and to counterbalance negative attitudes by positively perceived domains.

### Supplementary Information

Supplementary Information.

## Data Availability

The data that support the findings of this study are not publicly available. They are however available upon restrictions from the authors upon reasonable request and with permission of the French National Institute for Health and Medical Research (Inserm). Request should be sent to Prof. Judith E. Mueller (judith.mueller@ehesp.fr). The reuse of data is subject to compliance with the GDPR and French regulations.
