# Supplementary material for: Psychometric validation of a 7C-model of antecedents of vaccine acceptance among healthcare workers, parents and adolescents in France
Source: Sci Rep. 2023 Nov 14;13:19895. doi: 10.1038/s41598-023-46864-9 (PMC10646074; doi:10.1038/s41598-023-46864-9)
Supplement: Supplementary file 1 — Supplementary Information. [file 41598_2023_46864_MOESM1_ESM.docx]

**Psychometric Validation of a 7C-Model of Antecedents of Vaccine Acceptance Among Healthcare Workers, Parents and Adolescents in France.**

**Damien Oudin Doglioni**

1. Institut Pasteur, Université Paris Cité, Emerging Disease Epidemiology Unit, F-75015 Paris, France
2. Univ. Grenoble Alpes, Univ. Savoie Mont-Blanc, Laboratoire Interuniversitaire de Psychologie/Personnalité, Cognition, Changement social (LIP/PC2S), 38000 Grenoble, France

ORCID: https://orcid.org/0000-0003-3624-7251

e-mail: damien.oudin-doglioni@univ-grenoble-alpes.fr

**Amandine Gagneux-Brunon**

1. CHU de Saint-Étienne – Service d’infectiologie
2. Centre International de Recherche en Infectiologie, Team GIMAP, Université de Lyon, Université Jean Monnet, Université Claude Bernard Lyon 1, Inserm, U1111, CNRS, UMR530

ORCID: https://orcid.org/0000-0002-0892-2187

e-mail: amandine.gagneux-brunon@chu-st-etienne.fr

**Aurélie Gauchet**

1. Univ. Savoie Mont-Blanc, Univ. Grenoble Alpes, Laboratoire Interuniversitaire de Psychologie/Personnalité, Cognition, Changement social (LIP/PC2S), 38000 Grenoble, France

ORCID: https://orcid.org/0000-0003-1144-403X

e-mail: aurelie.gauchet@univ-smb.fr

**Sebastien Bruel**

1. Department of General Practice, Faculté de Médecine Jacques Lisfranc, Université Jean Monnet, Université de Lyon, Saint-Etienne, France.
2. Health, Systemic, Process UR 4129 Research Unit, University Claude Bernard, University of Lyon, Lyon, France.

ORCID: https://orcid.org/0000-0001-7413-5248

e-mail: bruel.sebastien@gmail.com

**Cyril Olivier**

1. GERES (Groupe d'Étude sur le Risque d'Exposition des Soignants), UFR de médecine Bichat, Paris

ORCID: https://orcid.org/0000-0001-9557-9227

e-mail: c.olivier@has-sante.fr

**Gérard Pellissier**

1. GERES (Groupe d'Étude sur le Risque d'Exposition des Soignants), UFR de médecine Bichat, Paris

ORCID: https://orcid.org/0000-0002-3872-4536

e-mail: g.pellissier@free.fr

**Nathalie Thilly**

1. Université de Lorraine, APEMAC, F-54000 Nancy, France.
2. Université de Lorraine, CHRU-Nancy, Département Méthodologie, Promotion, Investigation, F-54000 Nancy, France.

ORCID: https://orcid.org/0000-0003-1655-0624

e-mail: n.thilly@chru-nancy.fr

**Jonathan Sicsic**

1. Université Paris Cité, LIRAES, F-75006, Paris, France

ORCID: https://orcid.org/0000-0002-5509-4791

e-mail: jonathan.sicsic@u-paris.fr

**Jocelyn Raude**

1. Université de Rennes, EHESP, CNRS, Inserm, Arènes - UMR 6051, RSMS (Recherche sur les Services et Management en Santé) - U 1309 – F-35000 Rennes, France

ORCID: https://orcid.org/0000-0001-8020-4975

e-mail: jocelyn.raude@ehesp.fr

**Judith E. Mueller***

1. Institut Pasteur, Université Paris Cité, Emerging Disease Epidemiology Unit, F-75015 Paris, France

14. Université de Rennes, EHESP, CNRS, Inserm, Arènes - UMR 6051, RSMS (Recherche sur les Services et Management en Santé) - U 1309 – F-35000 Rennes, France

ORCID: 0000-0003-0797-9971

e-mail: judith.mueller@ehesp.fr

Table des matières

[1 Supplemental material 1: CFA items 5](#_Toc146527484)

[2 Supplemental material 2: Healthcare workers – COVID-19 9](#_Toc146527485)

[2.1 First-order latent structure 9](#_Toc146527486)

[2.1.1 Exploration of each antecedent 11](#_Toc146527487)

[2.1.2 Exploration of the theoretical model 12](#_Toc146527488)

[2.1.3 Latent factors scores estimation 21](#_Toc146527489)

[2.2 Second-order latent structure 26](#_Toc146527490)

[2.2.1 Definition of the latent factor 26](#_Toc146527491)

[2.2.2 Comparison with a 5 C theoretical model 28](#_Toc146527492)

[2.2.3 Comparison within the 7 C model 30](#_Toc146527493)

[2.3 Influence of VR and its antecedent on intention to be vaccinated against SARS-COV-2 31](#_Toc146527494)

[2.3.1 MLR with the antecedents of VR 31](#_Toc146527495)

[2.3.2 MLR with VR 35](#_Toc146527496)

[References 38](#_Toc146527497)

[3 Supplemental material 3: Parents – Human Papilloma Virus (HPV) 39](#_Toc146527498)

[3.1 First-order latent structure 39](#_Toc146527499)

[3.1.1 Exploration of each antecedent 41](#_Toc146527500)

[3.1.2 Exploration of the theoretical 7 C model 42](#_Toc146527501)

[3.1.3 Latent factors scores estimation 48](#_Toc146527502)

[3.2 Second-order latent structure 52](#_Toc146527503)

[3.2.1 Definition of the latent factor 53](#_Toc146527504)

[3.2.2 Comparison with a 5 C theoretical model 55](#_Toc146527505)

[3.2.3 Comparison within the 7 C model 57](#_Toc146527506)

[3.3 Influence of VR and its antecedent on intention to be vaccinated against HPV 58](#_Toc146527507)

[3.3.1 MLR with the antecedents of VR 59](#_Toc146527508)

[3.3.2 MLR with VR 62](#_Toc146527509)

[References 65](#_Toc146527510)

[4 Supplemental material 4: Adolescents – Human Papilloma Virus (HPV) 66](#_Toc146527511)

[4.1 First-order latent structure 66](#_Toc146527512)

[4.1.1 Exploration of each antecedents 68](#_Toc146527513)

[4.1.2 Exploration of the theoretical model 69](#_Toc146527514)

[4.1.3 Latent factors scores estimation 75](#_Toc146527515)

[4.2 Second-order latent structure 77](#_Toc146527516)

[4.2.1 Definition of the latent factor 77](#_Toc146527517)

[4.2.2 Comparison with a 5 C theoretical model 79](#_Toc146527518)

[4.2.3 Comparison within the 7C model 81](#_Toc146527519)

[4.3 Influence of VR and its antecedent on intention to be vaccinated against HPV 82](#_Toc146527520)

[4.3.1 MLR with the antecedents of VR 83](#_Toc146527521)

[4.3.2 MLR with VR 86](#_Toc146527522)

[References 88](#_Toc146527523)

[5 MLR resultat synthesis 89](#_Toc146527524)

[6 Vaccine readiness influence on acceptance and refusal (non-linear relationship) **Erreur ! Signet non défini.**](#_Toc146527525)

# Supplemental material 1: CFA items

**Questions used in the final CFA model to evaluate the seven psychological antecedents of vaccination among HCW (COVID-19), parents and adolescents (HPV)**

|  | **HCW (COVID-19)** | **Parents (HPV)** | **Adolescents (HPV)** |
| --- | --- | --- | --- |
| Distrust system | If my employer encourages me to get vaccinated, it will ... (answer : dissuasion gradient from dissuades me to motivates me).  On a scale of 0 to 10, how confident are you that the authorities will manage the health and economic crisis related to Covid-19? | (**Item A**) Generally speaking, would you be in favour of your child being vaccinated at school? (5-points Lickert scale).  (**Item B**) Do you agree with the statement 'the school system meets my child’s needs'? (5-points Lickert scale). | (**Item B**) Do you agree with the statement 'the school system meets my needs'? (5-points Lickert scale). |
| Distrust vaccine | I am afraid of having a severe side effect (5-points Lickert scale).  With new genetic technology new vaccines can easily be developed (yes/no/DNK answer).  The security of vaccines is monitored not only at the national level, but also in collaboration between other European countries (yes/no/DNK answer).  Severe side effects could appear beyond 6 months after vaccination (yes/no/DNK answer).  Economic considerations could lead to  recommendation of insufficiently evaluated vaccines (yes/no/DNK answer).  Some stages of vaccine development (control) have been skipped due to the epidemic emergency (yes/no/DNK answer). | I think the HPV vaccine is… (answer: gradient in five points from very dangerous to very safe). | I think the HPV vaccine is… (answer: gradient in five points from very dangerous to very safe). |
| Low convenience | In practice, it will be difficult for me to get the vaccine (5-points Lickert scale). | For me, vaccinating my child against HPV is… (answer: easiness gradient in 5-points from very complicated to very easy).  I have difficulties in talking about HPV vaccination with my child (answer: agreement gradient in 5-points).  I have difficulty talking about HPV vaccination with a HCW (answer: agreement gradient in 5-points). | Ideally, the HPV vaccine is given… (answer: five age ranges).  For me, being vaccinated against HPV is… (answer: easiness gradient in 5-points from very complicated to very easy). |
| High complacency | The gravity of the epidemic requires making vaccines quickly available (yes/no/DNK answer).  The efficacy of getting the vaccine for COVID-19 is higher than for the flu vaccine (yes/no/DNK answer).  On a scale of 0 to 10: globally, the coronavirus (COVID-19) epidemic in France worries you? | For me, to vaccinate my child against HPV is… (answer: usefulness gradient in 5-points).  I find my child too young to be vaccinated against HPV (answer: agreement gradient in 5-points). | What are the diseases caused by the HPV (answers: five diseases).  The vaccination against HPV is recommended for early adolescents cause the immune response is better before 14 y-o (yes/no/DNK answer).  For me, being vaccinated against HPV is… (answer: usefulness gradient in 5-points).  I find myself too young to be vaccinated against HPV (answer: agreement gradient in 5-points). |
| Low benefit-risk balance | I think that vaccination against COVID-19 will have more benefits than risks for me (5-points Lickert scale).  For a person with risk factors, these vaccines have more benefits than risks in the current epidemic situation (yes/no/DNK answer). | The vaccination against HPV have more benefits than risks, even for boys (answer: Yes/no/DNK).  I think the vaccination against HPV will have more benefits than risks for my child (answer: agreement gradient in 5-points). | I think the vaccination against HPV will have more benefits than risks for me (answer: agreement gradient in 5-points). |
| Self-centred vision | Getting vaccinated will also be a collective action to stop the crisis due to the epidemic (5-points Lickert scale). | If many young people are vaccinated against HPV, cancers caused by these viruses can be eliminated (answer: yes/no/DNK).  For me, the fact that the vaccine prevents the transmission of the HPV is… (answer: importance gradient in 5-points). | In the future, it will be important for me not to transmit the virus (answer: importance gradient in 5-points). |
| Sceptic environment | In your professional environment, how would you describe the majority opinion towards COVID-19 vaccination? (5-points Lickert scale).  In your familial and personal environment, how would you describe the majority opinion towards COVID-19? (5-points Lickert scale).  Do you know the approximate percentage of healthcare workers who will get the vaccine? (30%/60%/90%/DNK). | I find that the vaccine against HPV is now better accepted in France (answer: agreement gradient in 5-points). | The fact that others are vaccinated against HPV encourages me to get vaccinated (answer: agreement gradient in 5-points). |

# Supplemental material 2: Healthcare workers – COVID-19

## First-order latent structure

**Objective**: to identify a latent factorial organisation referring to the antecedents of vaccine readiness (VR) with a covariance relationship indicating the possible existence of a common formative second-order factor. As assessing the statistical significance of the loading for each indicator is important, we fixed the variance of the factor (Hoyle, 2012; Steiger, 2002) and freed all indicators. To estimate the factor score, we used the regression or exact method with Bartlett’s correction for bias in factor means (Estabrook & Neale, 2013). The score produced have a mean of zero and a free standard deviation. It is understood that each antecedent should be defined by, at least, one item as the objective is not to validate a questionnaire but a theoretical model.

We used the two-index presentation strategy (Hu & Bentler, 1999) including: the maximum likelihood (ML)‐based standardised root mean squared residual (SRMR) with a cut-off value under .08; and, the root mean square of approximation (RMSEA) with 90% confidence intervals and cut-off value close to .07 (Steiger, 2007). We added 3 indicators of goodness of fit (Hooper et al., 2008): the Wheaton *et al.*’s relative/normed chi square with a range from 2 to 5 (Wheaton et al., 1977); the comparative fit index (CFI) with a value greater than .90; and, the non-normed fit index (NNFI) also known as the Tucker-Lewis index (TLI) with a .90 threshold (Oudin Doglioni et al., 2021).

| **Indices** | **Presentation** | **Cut off** | **Reference** |
| --- | --- | --- | --- |
| SRMR | Absolute fit indice which determine how well an *a priori* model fits the sample data. The SRMR are the square root of the difference between the residuals of the sample covariance matrix and the hypothesised covariance model. | <0.08 | Hu & Bentler, 1999 |
| RMSEA | The RMSEA tells us how well the model, with unknown but optimally chosen parameter estimates would fit the populations covariance matrix. the RMSEA favours parsimony in that it will choose the model with the lesser number of parameters. | <0.07 | Steiger, 2007 |
| relative/normed chi square | The Chi-Square value is the traditional measure for evaluating overall model fit and, assesses the magnitude of discrepancy between the sample and fitted covariances matrices. Because the Chi-Square statistic is in essence a statistical significance test it is sensitive to sample size which means that the Chi-Square statistic nearly always rejects the model when large samples are used. One example of a statistic that minimises the impact of sample size on the model Chi-Square is Wheaton *et al*’s relative/normed chi-square (χ2/df). | [2;5] | Wheaton et al., 1977 |
| CFI | The Comparative Fit Index is a revised form of the NFI which takes into account sample size that performs well even when sample size is small. | >0.90 | Hu & Bentler, 1999 |
| NNFI/TLI | the Non-Normed Fit Index assesses the model by comparing the χ2 value of the model to the χ2 of the null model. | >0.90 | Hooper et al.; 2008 |

The statistics were carried out using Jamovi software (The Jamovi Project, 2020), version 2.3 or R Studio, version 3.6, both run under R (R Core Team, 2019), version 4.1, and the Laavan (Rosseel, 2012; Rosseel & Jorgensen, 2019), the Psych (Revelle, 2022), the Car libraries (Fox & Weisberg, 2019; fox & Weisberg, 2021) and the Emmeans library (Lenth et al., 2022).

The script used in Jamovi and R Studio are indicative. In particular, word written in italic refers to generic terms that must be adapted to the specific context.

*Script:*

| Model = ‘*factor* =~ *variable of interest*’  Model.fit = cfa(Model, data = *data*, missing = “ML”)  summary(Model.fit, fit.measures = TRUE)  Model.fit.predict = lavpredict(Model.fit, type = “lv”, method = “Bartlett”, label = TRUE, std.lv = TRUE) |
| --- |
| jmv::cfa(data = data,  factors = list(  list(label="*factor name*", vars=c("*variables of interest*")),  resCov = NULL,  stdEst = TRUE,  fitMeasures = c("cfi", "tli", "rmsea", "srmr", "aic")  mi = TRUE) |

### Exploration of each antecedent

#### Confidence in the vaccine

All indicators are significantly participating in the definition of the latent factor. Despite one indicator, most of them have loading higher than 0.30.

| **Factor** | **Indicator** | **Estimate** | **SE** | **Z** | **p** | **Stand. Estimate** |
| --- | --- | --- | --- | --- | --- | --- |
| Confidence vaccine | ZAEfear | 0.69922 | 0.01514 | 46.184 | <.001 | 0.69929 |
|  | ZKgentech | -0.45652 | 0.01547 | -29.517 | <.001 | -0.45657 |
|  | **ZKAEreport** | **-0.07468** | **0.01616** | **-4.623** | **<.001** | **-0.07469** |
|  | ZKAEsurveyEU | -0.3876 | 0.0157 | -24.686 | <.001 | -0.38763 |
|  | ZKAEsixmonth | -0.40692 | 0.01556 | -26.152 | <.001 | -0.40696 |
|  | Zconsideco | 0.49269 | 0.01531 | 32.176 | <.001 | 0.49274 |
|  | ZKskipcontrol | -0.66286 | 0.01517 | -43.694 | <.001 | -0.66292 |

The goodness of fit indices are above their thresholds indicating that the latent factor is well defined.

| **Antecedent** | **CFI** | **TLI** | **SRMR** | **RMSEA** | | | **Chi²/dF** | **AIC** |
| --- | --- | --- | --- | --- | --- | --- | --- | --- |
|  |  |  |  |  | 90% CI | |  |  |
|  | *>0.90* | *>0.90* | *<0.08* | *<0.07* | *Lower* | *Upper* | *<5* |  |
| Confidence vaccine | 0.9693 | 0.954 | 0.02161 | 0.04254 | 0.03646 | 0.0489 | 10.47 | 99813 |

#### Complacency

All indicators are significantly participating in the definition of the latent factor, but most of them load poorly on the latent factor.

| **Factor** | **Indicator** | **Estimate** | **SE** | **Z** | **p** | **Stand. Estimate** |
| --- | --- | --- | --- | --- | --- | --- |
| Complacency | Zseriousform | 0.45355 | 0.01859 | 24.394 | <.001 | 0.45359 |
|  | ZKepisevere | 0.39208 | 0.01758 | 22.306 | <.001 | 0.39211 |
|  | ZKlunchrisk | 0.38274 | 0.01969 | 19.434 | <.001 | 0.38277 |
|  | **ZKtouchrisk** | **0.2041** | **0.0179** | **11.401** | **<.001** | **0.20412** |
|  | **ZKbloodrisk** | **-0.03796** | **0.01777** | **-2.136** | **0.033** | **-0.03796** |
|  | ZKeventrisk | 0.43407 | 0.01952 | 22.235 | <.001 | 0.43411 |
|  | **ZKobeserisk** | **0.12994** | **0.01746** | **7.441** | **< .001** | **0.12996** |
|  | **ZKagerisk** | **0.09901** | **0.01753** | **5.648** | **<.001** | **0.09902** |
|  | **ZKlongsymp** | **0.09873** | **0.01746** | **5.654** | **<.001** | **0.09874** |
|  | **ZKfiftyrisk** | **0.17072** | **0.01742** | **9.8** | **<.001** | **0.17073** |
|  | **ZKVEflu** | **0.18107** | **0.0178** | **10.17** | **<.001** | **0.18109** |
|  | Zworry | 0.64334 | 0.02023 | 31.797 | <.001 | 0.6434 |

As a result, the goodness of fit indices are not above their thresholds indicating that the latent factor is misspecified.

| **Antecedent** | **CFI** | **TLI** | **SRMR** | **RMSEA** | | | **Chi²/dF** | **AIC** |
| --- | --- | --- | --- | --- | --- | --- | --- | --- |
|  |  |  |  |  | 90% CI | |  |  |
|  | *>0.90* | *>0.90* | *<0.08* | *<0.07* | *Lower* | *Upper* | *<5* |  |
| Complacency | 0.6354 | 0.5544 | 0.05016 | 0.069 | 0.0659 | 0.07215 | 25.93 | 175944 |

When fitting the theoretical model, low loading indicators should be considered for removal.

### Exploration of the theoretical model

#### Definition of the seven latent factor structure

##### Initial input (model 1)

All indicators are significantly participating in the definition of their respective latent factor. Indicators with loading (standard estimate) higher than 0.30 are of enough contribution. Indicators with loading under .30 should be removed in a Model 2 (standard estimate in bold).

| **Factor** | **Indicator** | **Estimate** | **SE** | **Z** | **p** | **Stand. Estimate** |
| --- | --- | --- | --- | --- | --- | --- |
| Confidence system | ZKemployer | 0.45237 | 0.01617 | 27.971 | <.001 | 0.45241 |
|  | Zconfiance | 0.60287 | 0.01778 | 33.898 | < .001 | 0.60292 |
| Confidence vaccine | ZAEfear | 0.74346 | 0.01323 | 56.192 | < .001 | 0.74353 |
|  | ZKgentech | -0.48086 | 0.01437 | -33.474 | <.001 | -0.48091 |
|  | **ZKAEreport** | **-0.07154** | **0.01521** | **-4.704** | **<.001** | **-0.07154** |
|  | ZKAEsurveyEU | -0.40085 | 0.01470 | -27.278 | <.001 | -0.40089 |
|  | ZKAEsixmonth | -0.38780 | 0.01465 | -26.474 | <.001 | -0.38784 |
|  | Zconsideco | 0.44447 | 0.01457 | 30.514 | <.001 | 0.44451 |
|  | ZKskipcontrol | -0.62605 | 0.01375 | -45.537 | <.001 | -0.62611 |
| Convenience | **Zdiffaccess** | **0.23337** | **0.03120** | **7.479** | **<.001** | **0.23340** |
|  | **ZKtwodoses** | **-0.23102** | **0.03095** | **-7.464** | **<.001** | **-0.23105** |
| Complacency | **Zseriousform** | **0.27564** | **0.01789** | **15.404** | **<.001** | **0.27567** |
|  | ZKepisevere | 0.59404 | 0.01511 | 39.305 | <.001 | 0.59410 |
|  | ZKlunchrisk | 0.33041 | 0.01633 | 20.236 | <.001 | 0.33045 |
|  | **ZKtouchrisk** | **0.04768** | **0.01730** | **2.756** | **0.006** | **0.04768** |
|  | **ZKbloodrisk** | **0.09116** | **0.01678** | **5.432** | **<.001** | **0.09117** |
|  | ZKeventrisk | 0.37070 | 0.01642 | 22.581 | <.001 | 0.37074 |
|  | **ZKobeserisk** | **0.14548** | **0.01610** | **9.033** | **< .001** | **0.14549** |
|  | **ZKagerisk** | **0.11268** | **0.01612** | **6.989** | **< .001** | **0.11269** |
|  | **ZKlongsymp** | **0.05930** | **0.01628** | **3.643** | **<.001** | **0.05930** |
|  | **ZKfiftyrisk** | **0.13747** | **0.01623** | **8.470** | **<.001** | **0.13748** |
|  | ZKVEflu | 0.34421 | 0.01668 | 20.642 | <.001 | 0.34424 |
|  | Zworry | 0.52152 | 0.01680 | 31.049 | <.001 | 0.52157 |
| Calculation | Zmorebenef | 0.74759 | 0.01389 | 53.813 | <.001 | 0.74766 |
|  | ZKBRifRF | 0.52822 | 0.01386 | 38.120 | <.001 | 0.52827 |
| Coll Responsibility | Zcollectstop | 1.06328 | 0.09797 | 10.853 | <.001 | 1.06339 |
|  | **ZKblocktrans** | **0.07257** | **0.01532** | **4.736** | **<.001** | **0.07258** |
| Social conformism | Zprofopinion | 0.63436 | 0.01466 | 43.264 | <.001 | 0.63442 |
|  | Zfamopinion | 0.76109 | 0.01436 | 53.013 | <.001 | 0.76116 |
|  | ZKVCHCW | -0.35535 | 0.01558 | -22.801 | <.001 | -0.35538 |

For the latent factor “Convenience”, we will keep the highest loaded indicator: “Zdiffaccess” which has a higher loading than “ZKtwodose”.

All factors covary, which suggests that they could refer to a second-order latent factor.

| **Factor** | | **Estimate** |  | **SE** | **Z** | **p** | **Stand. Estimate** |
| --- | --- | --- | --- | --- | --- | --- | --- |
| Confidence vaccine | Confidence vaccine | 1.0000 | ᵃ |  |  |  |  |
|  | Confidence system | -0.7387 |  | 0.02157 | -34.247 | <.001 | -0.7387 |
|  | Complacency | -0.6909 |  | 0.02146 | -32.199 | <.001 | -0.6909 |
|  | Convenience | 1.1787 |  | 0.14299 | 8.243 | <.001 | 1.1787 |
|  | Calculation | -0.9543 |  | 0.01291 | -73.900 | <.001 | -0.9543 |
|  | Coll. responsibility | -0.5762 |  | 0.05374 | -10.721 | <.001 | -0.5762 |
|  | Social conformism | 0.7268 |  | 0.01331 | 54.622 | <.001 | 0.7268 |
| Confidence system | Confidence system | 1.0000 | ᵃ |  |  |  |  |
|  | Complacency | 0.8172 |  | 0.02658 | 30.748 | <.001 | 0.8172 |
|  | Convenience | -0.9739 |  | 0.12828 | -7.592 | <.001 | -0.9739 |
|  | Calculation | 0.9203 |  | 0.02397 | 38.391 | <.001 | 0.9203 |
|  | Coll. responsibility | 0.6851 |  | 0.06594 | 10.389 | <.001 | 0.6851 |
|  | Social conformism | -0.6860 |  | 0.02279 | -30.108 | <.001 | -0.6860 |
| Complacency | Complacency | 1.0000 | ᵃ |  |  |  |  |
|  | Convenience | -1.1599 |  | 0.14568 | -7.962 | <.001 | -1.1599 |
|  | Calculation | 0.9213 |  | 0.02074 | 44.422 | <.001 | 0.9213 |
|  | Coll. responsibility | 0.6996 |  | 0.06577 | 10.637 | <.001 | 0.6996 |
|  | Social conformism | -0.6101 |  | 0.02106 | -28.966 | <.001 | -0.6101 |
| Convenience | Convenience | 1.0000 | ᵃ |  |  |  |  |
|  | Calculation | -1.2486 |  | 0.15243 | -8.191 | <.001 | -1.2486 |
|  | Coll. responsibility | -0.8041 |  | 0.12307 | -6.533 | <.001 | -0.8041 |
|  | Social conformism | 0.8806 |  | 0.11230 | 7.841 | <.001 | 0.8806 |
| Calculation | Calculation | 1.0000 | ᵃ |  |  |  |  |
|  | Coll. responsibility | 0.8363 |  | 0.07744 | 10.799 | <.001 | 0.8363 |
|  | Social conformism | -0.8075 |  | 0.01530 | -52.791 | <.001 | -0.8075 |
| Coll. responsibility | Coll. responsibility | 1.0000 | ᵃ |  |  |  |  |
|  | Social conformism | -0.5279 |  | 0.04995 | -10.569 | <.001 | -0.5279 |
| ᵃ fixed parameter |  |  |  |  |  |  |  |

Goodness of fit indices do not reach the thresholds to indicate a good fit of the theoretical model in our population.

|  | **CFI** | **TLI** | **SRMR** | **RMSEA** | | | **Chi²/dF** | **AIC** |
| --- | --- | --- | --- | --- | --- | --- | --- | --- |
|  |  |  |  |  | 90% CI | |  |  |
|  | *>0.90* | *>0.90* | *<0.08* | *<0.07* | *Lower* | *Upper* | *<5* |  |
| Initial model | **0.8245** | **0.8012** | 0.04876 | 0.04889 | 0.04771 | 0.05007 | 13.5078 | 423174 |

To improve the fit, low loading indicators were removed in a second model.

##### Removal of non-contributory indicators (model 2)

All indicators contribute to the definition of their respective latent factors and loading are all above 0.30.

| **Factor** | **Indicator** | **Estimate** | **SE** | **Z** | **p** | **Stand. Estimate** |
| --- | --- | --- | --- | --- | --- | --- |
| Confidence system | ZKemployer | 0.4530 | 0.016184 | 27.99 | <.001 | 0.4530 |
|  | Zconfiance | 0.6020 | 0.017783 | 33.85 | < .001 | 0.6021 |
| Confidence vaccine | ZAEfear | 0.7388 | 0.013217 | 55.90 | <.001 | 0.7389 |
|  | ZKgentech | -0.4821 | 0.014353 | -33.59 | <.001 | -0.4822 |
|  | ZKAEsurveyEU | -0.4032 | 0.014671 | -27.48 | <.001 | -0.4032 |
|  | ZKAEsixmonth | -0.3879 | 0.014654 | -26.47 | <.001 | -0.3880 |
|  | Zconsideco | 0.4458 | 0.014570 | 30.60 | <.001 | 0.4458 |
|  | ZKskipcontrol | -0.6286 | 0.013730 | -45.78 | <.001 | -0.6287 |
| Convenience | Zdiffaccess | 0.9999 | 0.009773 | 102.31 | <.001 | 1.0000 |
| Complacency | ZKepisevere | 0.6016 | 0.015061 | 39.95 | <.001 | 0.6017 |
|  | ZKlunchrisk | 0.3049 | 0.016279 | 18.73 | <.001 | 0.3049 |
|  | ZKeventrisk | 0.3411 | 0.016443 | 20.74 | <.001 | 0.3411 |
|  | ZKVEflu | 0.3704 | 0.015947 | 23.22 | <.001 | 0.3704 |
|  | Zworry | 0.4568 | 0.015888 | 28.75 | <.001 | 0.4568 |
| Calculation | Zmorebenef | 0.7504 | 0.013903 | 53.97 | <.001 | 0.7505 |
|  | ZKBRifRF | 0.5263 | 0.013861 | 37.97 | <.001 | 0.5263 |
| Coll Responsibility | Zcollectstop | 0.9999 | 0.009773 | 102.31 | <.001 | 1.0000 |
| Social conformism | Zprofopinion | 0.6344 | 0.014667 | 43.25 | <.001 | 0.6344 |
|  | Zfamopinion | 0.7610 | 0.014363 | 52.98 | <.001 | 0.7611 |
|  | ZKVCHCW | -0.3556 | 0.015590 | -22.81 | <.001 | -0.3556 |

As for the first model, all factors covary suggesting that they refer to a latent second order.

| **Factor** | | **Estimate** |  | **SE** | **Z** | **p** | **Stand. Estimate** |
| --- | --- | --- | --- | --- | --- | --- | --- |
| Confidence vaccine | Confidence vaccine | 1.0000 | ᵃ |  |  |  |  |
|  | Confidence system | -0.7330 |  | 0.02156 | -34.00 | <.001 | -0.7330 |
|  | Complacency | -0.5962 |  | 0.01949 | -30.59 | <.001 | -0.5962 |
|  | Convenience | 0.3097 |  | 0.01483 | 20.88 | <.001 | 0.3097 |
|  | Calculation | -0.9490 |  | 0.01286 | -73.78 | <.001 | -0.9490 |
|  | Coll. responsibility | -0.6081 |  | 0.01132 | -53.74 | <.001 | -0.6081 |
|  | Social conformism | 0.7264 |  | 0.01327 | 54.74 | <.001 | 0.7264 |
| Confidence system | Confidence system | 1.0000 | ᵃ |  |  |  |  |
|  | Complacency | 0.8474 |  | 0.02484 | 34.12 | <.001 | 0.8474 |
|  | Convenience | -0.2630 |  | 0.02009 | -13.09 | <.001 | -0.2630 |
|  | Calculation | 0.9130 |  | 0.02388 | 38.24 | <.001 | 0.9130 |
|  | Coll. responsibility | 0.7235 |  | 0.01829 | 39.55 | <.001 | 0.7235 |
|  | Social conformism | -0.6827 |  | 0.02275 | -30.00 | <.001 | -0.6827 |
| Complacency | Complacency | 1.0000 | ᵃ |  |  |  |  |
|  | Convenience | -0.1911 |  | 0.01825 | -10.47 | <.001 | -0.1911 |
|  | Calculation | 0.8704 |  | 0.01879 | 46.31 | <.001 | 0.8704 |
|  | Coll. responsibility | 0.7334 |  | 0.01297 | 56.53 | <.001 | 0.7334 |
|  | Social conformism | -0.5623 |  | 0.01975 | -28.47 | <.001 | -0.5623 |
| Convenience | Convenience | 1.0000 | ᵃ |  |  |  |  |
|  | Calculation | -0.2731 |  | 0.01667 | -16.38 | <.001 | -0.2731 |
|  | Coll. responsibility | -0.1911 |  | 0.01332 | -14.35 | <.001 | -0.1911 |
|  | Social conformism | 0.1897 |  | 0.01621 | 11.70 | <.001 | 0.1897 |
| Calculation | Calculation | 1.0000 | ᵃ |  |  |  |  |
|  | Coll. responsibility | 0.8825 |  | 0.01042 | 84.67 | <.001 | 0.8825 |
|  | Social conformism | -0.8041 |  | 0.01524 | -52.75 | <.001 | -0.8041 |
| Coll. responsibility | Coll. responsibility | 1.0000 | ᵃ |  |  |  |  |
|  | Social conformism | -0.5591 |  | 0.01272 | -43.96 | <.001 | -0.5591 |
| ᵃ fixed parameter |  |  |  |  |  |  |  |

TLI does not reach the threshold to indicate a good fit of the theoretical model in our population.

|  | **CFI** | **TLI** | **SRMR** | **RMSEA** | | | **Chi²/dF** | **AIC** |
| --- | --- | --- | --- | --- | --- | --- | --- | --- |
|  |  |  |  |  | 90% CI | |  |  |
|  | *>0.90* | *>0.90* | *<0.08* | *<0.07* | *Lower* | *Upper* | *<5* |  |
| Model 2 | 0.9015 | **0.8760** | 0.04375 | 0.05479 | 0.05292 | 0.05668 | 16.71 | 275468 |

Modification indices suggest adding covariance between indicators from the same latent factors which was done in a Model 3:

- **ZKeventrisk** (‘For the end of the year 2020, indicate for the next situations the level of risk of infection with the SARS-COV-2 virus: “Assisting in a festive event of 30 people.”’ [Complacency]) and **ZKlunchrisk** (‘For the end of the year 2020, indicate for the next situations the level of risk of infection with the SARS-COV-2 virus: “Taking a lunch break or an apéro with colleagues in the service.”’ [Complacency]) suggesting a link between enclosed space, presence of people and risk of contamination in HCW (MI = 424.4534);
- **ZAEfear** (‘I am afraid of having a severe side effect’ [Confidence vaccine]) and **Zworry** (‘On a scale from 0 to 10: Globally, the coronavirus (COVID-19) epidemic in France worries you?’ [Complacency]) suggesting a cognitive process between fear of a severe side effect and worry about the COVID-19 pandemic (MI = 188.770).

These two additions were made in a third model.

##### Addition of covariance between indicators (model 3)

*Script:*

| Model = ‘*factor* =~ *variable of interest*  *indicator~~indicator*’  Model.fit = cfa(Model, data = *data*, missing = “ML”)  summary(Model.fit, fit.measures = TRUE)  modindices(Model.fit, sort = TRUE, maximum.number = 5)  Model.fit.predict = lavpredict(Model.fit, type = “lv”, method = “Bartlett”, label = TRUE, std.lv = TRUE) |
| --- |
| jmv::cfa(data = data,  factors = list(  list(label="*factor name*", vars=c("*variables of interest*")),  resCov = list (  list (i_n_ = "*variables of interest*")),  stdEst = TRUE,  fitMeasures = c("cfi", "tli", "rmsea", "srmr", "aic")  mi = TRUE) |

All indicators significantly contribute to the definition of their respective latent factor, however, with the addition of covariance, two indicators show loading under 0.30. They should be removed in a model 4. After removal, no covariance between indicators will be kept.

| **Factor** | **Indicator** | **Estimate** | **SE** | **Z** | **p** | **Stand. Estimate** |
| --- | --- | --- | --- | --- | --- | --- |
| Confidence system | ZKemployer | 0.4548 | 0.016191 | 28.09 | <.001 | 0.4548 |
|  | Zconfiance | 0.5997 | 0.01774 | 33.8 | < .001 | 0.5997 |
| Confidence vaccine | ZAEfear | 0.7243 | 0.013116 | 55.22 | <.001 | 0.7286 |
|  | ZKgentech | -0.4829 | 0.014304 | -33.76 | <.001 | -0.4829 |
|  | ZKAEsurveyEU | -0.4077 | 0.014601 | -27.92 | <.001 | -0.4077 |
|  | ZKAEsixmonth | -0.3871 | 0.014638 | -26.45 | <.001 | -0.3871 |
|  | Zconsideco | 0.4413 | 0.01458 | 30.27 | <.001 | 0.4414 |
|  | ZKskipcontrol | -0.6265 | 0.013708 | -45.7 | <.001 | -0.6266 |
| Convenience | Zdiffaccess | 0.9999 | 0.009773 | 102.31 | <.001 | 1.000 |
| Complacency | ZKepisevere | 0.5778 | 0.015598 | 37.04 | <.001 | 0.5779 |
|  | **ZKlunchrisk** | **0.2556** | **0.015713** | **16.26** | **<.001** | **0.2556** |
|  | **ZKeventrisk** | **0.2869** | **0.015991** | **17.94** | **<.001** | **0.2869** |
|  | ZKVEflu | 0.3891 | 0.015357 | 25.34 | <.001 | 0.3891 |
|  | Zworry | 0.4214 | 0.015711 | 26.82 | <.001 | 0.4224 |
| Calculation | Zmorebenef | 0.752 | 0.013892 | 54.13 | <.001 | 0.752 |
|  | ZKBRifRF | 0.5251 | 0.013856 | 37.9 | <.001 | 0.5252 |
| Coll Responsibility | Zcollectstop | 0.9999 | 0.009773 | 102.31 | <.001 | 1.000 |
| Social conformism | Zprofopinion | 0.6334 | 0.01467 | 43.18 | <.001 | 0.6335 |
|  | Zfamopinion | 0.762 | 0.014369 | 53.03 | <.001 | 0.7621 |
|  | ZKVCHCW | -0.3552 | 0.015594 | -22.78 | <.001 | -0.3552 |

Again, all factors covary suggesting the existence of a second-order latent factor.

| **Factor** | | **Estimate** |  | **SE** | **Z** | **p** | **Stand. Estimate** |
| --- | --- | --- | --- | --- | --- | --- | --- |
| Confidence vaccine | Confidence vaccine | 1 | ᵃ |  |  |  |  |
|  | Confidence system | -0.7492 |  | 0.02152 | -34.82 | <.001 | -0.7492 |
|  | Complacency | -0.7012 |  | 0.02074 | -33.8 | <.001 | -0.7012 |
|  | Convenience | 0.3077 |  | 0.01487 | 20.68 | <.001 | 0.3077 |
|  | Calculation | -0.9543 |  | 0.01281 | -74.49 | <.001 | -0.9543 |
|  | Coll. responsibility | -0.6143 |  | 0.01125 | -54.58 | <.001 | -0.6143 |
|  | Social conformism | 0.727 |  | 0.01328 | 54.74 | <.001 | 0.727 |
| Confidence system | Confidence system | 1 | ᵃ |  |  |  |  |
|  | Complacency | 0.8905 |  | 0.02634 | 33.8 | <.001 | 0.8905 |
|  | Convenience | -0.2633 |  | 0.02012 | -13.09 | <.001 | -0.2633 |
|  | Calculation | 0.9135 |  | 0.02384 | 38.32 | <.001 | 0.9135 |
|  | Coll. responsibility | 0.7246 |  | 0.01828 | 39.63 | <.001 | 0.7246 |
|  | Social conformism | -0.6839 |  | 0.02274 | -30.08 | <.001 | -0.6839 |
| Complacency | Complacency | 1 | ᵃ |  |  |  |  |
|  | Convenience | -0.204 |  | 0.01901 | -10.73 | <.001 | -0.204 |
|  | Calculation | 0.9369 |  | 0.01989 | 47.11 | <.001 | 0.9369 |
|  | Coll. responsibility | 0.7778 |  | 0.01383 | 56.24 | <.001 | 0.7778 |
|  | Social conformism | -0.6192 |  | 0.02044 | -30.29 | <.001 | -0.6192 |
| Convenience | Convenience | 1 | ᵃ |  |  |  |  |
|  | Calculation | -0.2727 |  | 0.01665 | -16.37 | <.001 | -0.2727 |
|  | Coll. responsibility | -0.1911 |  | 0.01332 | -14.35 | <.001 | -0.1911 |
|  | Social conformism | 0.1897 |  | 0.0162 | 11.71 | <.001 | 0.1897 |
| Calculation | Calculation | 1 | ᵃ |  |  |  |  |
|  | Coll. responsibility | 0.8815 |  | 0.01042 | 84.62 | <.001 | 0.8815 |
|  | Social conformism | -0.8031 |  | 0.01523 | -52.74 | <.001 | -0.8031 |
| Coll. responsibility | Coll. responsibility | 1 | ᵃ |  |  |  |  |
|  | Social conformism | -0.559 |  | 0.01272 | -43.96 | <.001 | -0.559 |
| ᵃ fixed parameter |  |  |  |  |  |  |  |

The goodness of fit indices do reach the thresholds to indicate a good fit of the theoretical model in our population.

|  | **CFI** | **TLI** | **SRMR** | **RMSEA** | | | **Chi²/dF** | **AIC** |
| --- | --- | --- | --- | --- | --- | --- | --- | --- |
|  |  |  |  |  | 90% CI | |  |  |
|  | *>0.90* | *>0.90* | *<0.08* | *<0.07* | *Lower* | *Upper* | *<5* |  |
| Model 3 | 0.9278 | 0.9079 | 0.03632 | 0.04722 | 0.04533 | 0.04914 | 12.67 | 274837 |

Although Wheaton *et al*’s relative/normed chi-square (Chi2/dF) minimises the impact of sample size on the model, in the CappVaCov database sample size is too large (n> 5000). However, despite goodness of fit indices indicating a good fit of the theoretical model to the CappVaCov database, low loading indicators should be removed in a final model.

##### Final model (model 4)

All indicators significantly contribute to the definition of their respective latent factor and no indicator show loading under 0.30.

| **Factor** | **Indicator** | **Estimate** | **SE** | **Z** | **p** | **Stand. Estimate** |
| --- | --- | --- | --- | --- | --- | --- |
| Confidence system | ZKemployer | 0.458 | 0.016224 | 28.23 | <.001 | 0.4581 |
|  | Zconfiance | 0.5954 | 0.017693 | 33.65 | < .001 | 0.5955 |
| Confidence vaccine | ZAEfear | 0.7249 | 0.013091 | 55.37 | <.001 | 0.7291 |
|  | ZKgentech | -0.4832 | 0.014288 | -33.82 | <.001 | -0.4833 |
|  | ZKAEsurveyEU | -0.4081 | 0.014578 | -27.99 | <.001 | -0.4081 |
|  | ZKAEsixmonth | -0.3868 | 0.014627 | -26.45 | <.001 | -0.3869 |
|  | Zconsideco | 0.4403 | 0.014578 | 30.2 | <.001 | 0.4403 |
|  | ZKskipcontrol | -0.6256 | 0.013706 | -45.64 | <.001 | -0.6257 |
| Convenience | Zdiffaccess | 0.9999 | 0.009773 | 102.31 | <.001 | 1 |
| Complacency | ZKepisevere | 0.5411 | 0.016653 | 32.49 | <.001 | 0.5412 |
|  | ZKVEflu | 0.3879 | 0.015237 | 25.46 | <.001 | 0.3879 |
|  | Zworry | 0.3857 | 0.015925 | 24.22 | <.001 | 0.3862 |
| Calculation | Zmorebenef | 0.7528 | 0.013882 | 54.23 | <.001 | 0.7529 |
|  | ZKBRifRF | 0.5246 | 0.013851 | 37.87 | <.001 | 0.5246 |
| Coll Responsibility | Zcollectstop | 0.9999 | 0.009773 | 102.31 | <.001 | 1 |
| Social conformism | Zprofopinion | 0.6336 | 0.014663 | 43.21 | <.001 | 0.6337 |
|  | Zfamopinion | 0.7615 | 0.014362 | 53.02 | <.001 | 0.7616 |
|  | ZKVCHCW | -0.3559 | 0.015592 | -22.83 | <.001 | -0.356 |

Again, all factors covary suggesting the existence of a second-order latent factor.

| **Factor** | | **Estimate** |  | **SE** | **Z** | **p** | **Stand. Estimate** |
| --- | --- | --- | --- | --- | --- | --- | --- |
| Confidence vaccine | Confidence vaccine | 1 | ᵃ |  |  |  |  |
|  | Confidence system | -0.7525 |  | 0.0215 | -34.99 | <.001 | -0.7525 |
|  | Complacency | -0.7752 |  | 0.02364 | -32.78 | <.001 | -0.7752 |
|  | Convenience | 0.308 |  | 0.01487 | 20.71 | <.001 | 0.308 |
|  | Calculation | -0.9543 |  | 0.0128 | -74.56 | <.001 | -0.9543 |
|  | Coll. responsibility | -0.6154 |  | 0.01124 | -54.77 | <.001 | -0.6154 |
|  | Social conformism | 0.7269 |  | 0.01328 | 54.72 | <.001 | 0.7269 |
| Confidence system | Confidence system | 1 | ᵃ |  |  |  |  |
|  | Complacency | 0.9424 |  | 0.03004 | 31.37 | <.001 | 0.9424 |
|  | Convenience | -0.2639 |  | 0.02016 | -13.09 | <.001 | -0.2639 |
|  | Calculation | 0.9155 |  | 0.02382 | 38.44 | <.001 | 0.9155 |
|  | Coll. responsibility | 0.7265 |  | 0.01827 | 39.77 | <.001 | 0.7265 |
|  | Social conformism | -0.6862 |  | 0.02274 | -30.18 | <.001 | -0.6862 |
| Complacency | Complacency | 1 | ᵃ |  |  |  |  |
|  | Convenience | -0.2127 |  | 0.02059 | -10.33 | <.001 | -0.2127 |
|  | Calculation | 1.0123 |  | 0.02352 | 43.05 | <.001 | 1.0123 |
|  | Coll. responsibility | 0.825 |  | 0.0169 | 48.81 | <.001 | 0.825 |
|  | Social conformism | -0.6853 |  | 0.02276 | -30.12 | <.001 | -0.6853 |
| Convenience | Convenience | 1 | ᵃ |  |  |  |  |
|  | Calculation | -0.2724 |  | 0.01664 | -16.37 | <.001 | -0.2724 |
|  | Coll. responsibility | -0.1911 |  | 0.01332 | -14.35 | <.001 | -0.1911 |
|  | Social conformism | 0.1897 |  | 0.01621 | 11.7 | <.001 | 0.1897 |
| Calculation | Calculation | 1 | ᵃ |  |  |  |  |
|  | Coll. responsibility | 0.881 |  | 0.01041 | 84.61 | <.001 | 0.881 |
|  | Social conformism | -0.8027 |  | 0.01522 | -52.74 | <.001 | -0.8027 |
| Coll. responsibility | Coll. responsibility | 1 | ᵃ |  |  |  |  |
|  | Social conformism | -0.5591 |  | 0.01272 | -43.96 | <.001 | -0.5591 |
| ᵃ fixed parameter |  |  |  |  |  |  |  |

The goodness of fit indices do reach the thresholds to indicate a good fit of the theoretical model in our population.

|  | **CFI** | **TLI** | **SRMR** | **RMSEA** | | | **Chi²/dF** | **AIC** |
| --- | --- | --- | --- | --- | --- | --- | --- | --- |
|  |  |  |  |  | 90% CI | |  |  |
|  | *>0.90* | *>0.90* | *<0.08* | *<0.07* | *Lower* | *Upper* | *<5* |  |
| Model 4 | 0.9340 | 0.9122 | 0.03451 | 0.04988 | 0.04774 | 0.05206 | 14.02 | 246244 |

Although Wheaton *et al*’s relative/normed chi-square (Chi2/dF) minimises the impact of sample size on the model, in the CappVaCov database sample size is too large (n> 5000). In French Healthcare workers, a latent organisation on seven first-order factors is demonstrated.

##### CFA synthesis

Definition of the seven latent factor structure shows constant improvement of the fit to the data.

|  | **French healthcare workers (CappVaCov database)** | | | | | | | |
| --- | --- | --- | --- | --- | --- | --- | --- | --- |
| **Model** | **CFI** | **TLI** | **SRMR** | **RMSEA** | | | **Chi²/dF** | **AIC** |
|  |  |  |  |  | 90% CI | |  |  |
|  | *>0.90* | *>0.90* | *<0.08* | *<0.07* | *Lower* | *Upper* | *<5* |  |
| Initial | 0.8245 | 0.8012 | 0.04876 | 0.04889 | 0.04771 | 0.05007 | 13.5078 | 423174 |
| #2 | 0.9015 | 0.8760 | 0.04375 | 0.05479 | 0.05292 | 0.05668 | 16.71 | 275468 |
| #3 | 0.9278 | 0.9079 | 0.03632 | 0.04722 | 0.04533 | 0.04914 | 12.67 | 274837 |
| final | 0.9340 | 0.9122 | 0.03451 | 0.04988 | 0.04774 | 0.05206 | 14.02 | 246244 |

#### First-order latent structure internal consistency

*Script:*

| jmv::reliability(data = data,  vars = vars(*variables of interest*),  alphaScale = FALSE,  omegaScale = TRUE) |
| --- |

| **Internal consistency – 1^st^ order** | **McDonald’s ώ** |
| --- | --- |
| French healthcare workers | 0.8530 |

Reliability analysis demonstrates a good internal consistency of the first-order latent structure.

### Latent factors scores estimation

How to read the scores according to the item meaning:

| **Indicator** | **A high score to the indicator indicates:** | **A high score to the factor indicates:** |
| --- | --- | --- |
| ZAEfear | Fear of having serious side effects | Lack of confidence in the vaccine |
| ZKgentech (R) | With new genetic technology, new vaccines cannot easily be developed. |  |
| ZKAEsurveyEU (R) | Vaccines are not monitored |  |
| ZKAEsixmonth (R) | Severe side effects could appear past 6 months |  |
| Zconsideco | Economic reasons for insufficiently evaluated vaccines |  |
| ZKskipcontrol (R) | Stages of vaccine development have not been skip |  |
| ZKemployer | Employers incitation encourage getting vaccinated | Confidence in the system |
| Zconfiance | Confidence in authorities to manage the crisis |  |
| ZKepisevere | The gravity of the epidemic requires vaccines quickly | Low complacency |
| ZKVEflu | Higher efficacy than flu vaccine |  |
| Zworry | Worries about the epidemic in France |  |
| Zdiffaccess | Getting vaccinated will be difficult | Perceived barriers to get vaccinated |
| Zmorebenef | Perception of the benefice of getting vaccinated | perceived benefice over risk of getting vaccinated |
| ZKBRifRF | Perception of the benefice of the vaccine for person with risk factors |  |
| Zcollectstop | Perception of the potential herd immunity | Perception of the collective benefice of getting vaccinated |
| Zprofopinion | The perception that the professional environment is sceptic toward vaccination | The social norm is to be a sceptic toward vaccination |
| Zfamopinion | The perception that family and/or friends are sceptic toward vaccination |  |
| ZKVCHCW (R) | Perception of low adhesion to vaccination |  |
| (R): reverse loading meaning inverse interpretation. | |  |

In order to respect the meaning of the vaccine readiness concept, reverse coding must be applied:

| High VR when | High | Confidence vaccine | Reverse | Confidence vaccine |
| --- | --- | --- | --- | --- |
|  | High | Confidence system |  | Confidence system |
|  | Low | Complacency |  | Low complacency |
|  | Low | Convenience | Reverse | High convenience |
|  | High | Calculation |  | High balance benefice risk (BRB) |
|  | High | Collective responsibility |  | Collective responsibility |
|  | Low | Social conformism | Reverse | Favourable environment |

Mean score per subpopulation:

| **HCW** | **Confidence system** | **Confidence vaccine** | **High convenience** | **Low complacency** | **High BRB** | **Collective responsibility** | **Favourable environment** |
| --- | --- | --- | --- | --- | --- | --- | --- |
| Overall | 0.000 | 0.000 | 0.000 | 0.000 | 0.000 | 0.000 | 0.000 |
| MD | 0.358 | 0.780 | 0.218 | 0.625 | 0.591 | 0.380 | 0.731 |
| Other biomed.^2^ | 0.246 | 0.466 | 0.073 | 0.410 | 0.479 | 0.319 | 0.656 |
| Nurse | -0.315 | -0.130 | -0.078 | -0.159 | -0.169 | -0.109 | -0.382 |
| Nurse assistant | -0.567 | -0.783 | -0.285 | -0.672 | -0.754 | -0.609 | -0.682 |
| Other HCW^1^ | -0.030 | -0.311 | -0.043 | -0.327 | -0.193 | -0.115 | -0.055 |
| ^1,^ for example radio manipulator, laboratory technician, psychologist, other scientific support functions  ^2^ include pharmacist, midwife, biologist, dentist | | | | | | | |

Kiviat diagram suggests the existence of three groups that seems to follow a gradient of literacy.

Per subpopulations mean comparison show significant differences for all the factors.

*Script:*

| jmv::ANOVA(formula = `*factor of interest*` ~ Profession_group,  data = data,  postHoc = ~ Profession_group,  postHocCorr = "bonf",  emMeans = ~ Profession_group,  emmTables = TRUE) |
| --- |

| **French HCW** | **ANOVA** | | | **P-value** | **ή²** |
| --- | --- | --- | --- | --- | --- |
|  | Df1 | Df2 | F test |  |  |
| Confidence vaccine | 4 | 3865 | 48.67 | <0.001 | 0.0480^*^ |
| Confidence system | 4 | 3865 | 232.5 | <0.001 | 0.1940^***^ |
| Low complacency | 4 | 3865 | 23.00 | <0.001 | 0.0233^*^ |
| High convenience | 4 | 3865 | 85.34 | <0.001 | 0.0812^**^ |
| High BRB | 4 | 3865 | 133.2 | <0.001 | 0.1211^**^ |
| Collective responsibility | 4 | 3865 | 102.6 | <0.001 | 0.0960^**^ |
| Favourable environment | 4 | 3865 | 216.6 | <0.001 | 0.1831^***^ |
| Effect size, ή²: ^***^large - ^**^medium - ^*^small | | | | | |

Post-hoc analysis with a Bonferroni correction tends to show three groups that seems to be related to the level of education. First group “MD-Biomedical” with lower VR, the second group “Nurse-Other paramedics” with a mild VR and the third group “nurse assistant” with high VR.

| **French HCW** | | **Mean Difference** | **SE** | **dF** | **t** | **p_bonferroni_** |
| --- | --- | --- | --- | --- | --- | --- |
| **Confidence system** | | | | | | |
| MD | Biomedical | 0.1117 | 0.08134 | 3865 | -1.373 | 1.000 |
|  | Nurse | 0.6727 | 0.06399 | 3865 | -10.514 | <.001 |
|  | Nurse assistant | 0.9248 | 0.08113 | 3865 | -11.399 | <.001 |
|  | Other HCW | 0.3882 | 0.06995 | 3865 | -5.55 | <.001 |
| Biomedical | Nurse | 0.561 | 0.07735 | 3865 | -7.254 | <.001 |
|  | Nurse assistant | 0.8131 | 0.09203 | 3865 | -8.835 | <.001 |
|  | Other HCW | 0.2765 | 0.08235 | 3865 | -3.358 | 0.008 |
| Nurse | Nurse assistant | 0.2521 | 0.07712 | 3865 | -3.268 | 0.011 |
|  | Other HCW | -0.2845 | 0.06526 | 3865 | 4.36 | <.001 |
| Nurse assistant | Other HCW | -0.5366 | 0.08214 | 3865 | 6.533 | <.001 |
| **Confidence vaccine** | | | | | | |
| MD | Biomedical | 0.3132 | 0.05964 | 3865 | -5.252 | <.001 |
|  | Nurse | 0.9094 | 0.04691 | 3865 | -19.385 | <.001 |
|  | Nurse assistant | 1.5629 | 0.05948 | 3865 | -26.275 | <.001 |
|  | Other HCW | 1.0905 | 0.05129 | 3865 | -21.264 | <.001 |
| Biomedical | Nurse | 0.5962 | 0.05671 | 3865 | -10.514 | <.001 |
|  | Nurse assistant | 1.2497 | 0.06748 | 3865 | -18.52 | <.001 |
|  | Other HCW | 0.7773 | 0.06038 | 3865 | -12.874 | <.001 |
| Nurse | Nurse assistant | 0.6535 | 0.05655 | 3865 | -11.557 | <.001 |
|  | Other HCW | 0.1811 | 0.04785 | 3865 | -3.784 | 0.002 |
| Nurse assistant | Other HCW | -0.4724 | 0.06022 | 3865 | 7.844 | <.001 |
| **High convenience** | | | | | | |
| MD | Biomedical | 0.14492 | 0.05672 | 3865 | -2.5551 | 0.107 |
|  | Nurse | 0.29655 | 0.04461 | 3865 | -6.6469 | <.001 |
|  | Nurse assistant | 0.50321 | 0.05657 | 3865 | -8.8957 | <.001 |
|  | Other HCW | 0.26167 | 0.04877 | 3865 | -5.3651 | <.001 |
| Biomedical | Nurse | 0.15163 | 0.05393 | 3865 | -2.8116 | 0.05 |
|  | Nurse assistant | 0.35829 | 0.06417 | 3865 | -5.5834 | <.001 |
|  | Other HCW | 0.11675 | 0.05742 | 3865 | -2.0333 | 0.421 |
| Nurse | Nurse assistant | 0.20666 | 0.05377 | 3865 | -3.8431 | 0.001 |
|  | Other HCW | -0.03488 | 0.0455 | 3865 | 0.7667 | 1.000 |
| Nurse assistant | Other HCW | -0.24154 | 0.05727 | 3865 | 4.2176 | <.001 |
| **Low complacency** | | | | | | |
| MD | Biomedical | 0.2151 | 0.0845 | 3865 | -2.545 | 0.11 |
|  | Nurse | 0.7842 | 0.06647 | 3865 | -11.798 | <.001 |
|  | Nurse assistant | 1.2968 | 0.08427 | 3865 | -15.388 | <.001 |
|  | Other HCW | 0.9515 | 0.07266 | 3865 | -13.095 | <.001 |
| Biomedical | Nurse | 0.5691 | 0.08035 | 3865 | -7.083 | <.001 |
|  | Nurse assistant | 1.0818 | 0.0956 | 3865 | -11.316 | <.001 |
|  | Other HCW | 0.7364 | 0.08554 | 3865 | -8.609 | <.001 |
| Nurse | Nurse assistant | 0.5127 | 0.08011 | 3865 | -6.4 | <.001 |
|  | Other HCW | 0.1673 | 0.06779 | 3865 | -2.468 | 0.136 |
| Nurse assistant | Other HCW | -0.3454 | 0.08532 | 3865 | 4.048 | <.001 |
| **High benefit-risk balance** | | | | | | |
| MD | Biomedical | 0.11226 | 0.06758 | 3865 | -1.6613 | 0.967 |
|  | Nurse | 0.75983 | 0.05316 | 3865 | -14.2937 | <.001 |
|  | Nurse assistant | 1.34536 | 0.0674 | 3865 | -19.9607 | <.001 |
|  | Other HCW | 0.78392 | 0.05811 | 3865 | -13.49 | <.001 |
| Biomedical | Nurse | 0.64756 | 0.06426 | 3865 | -10.0775 | <.001 |
|  | Nurse assistant | 1.23309 | 0.07646 | 3865 | -16.1272 | <.001 |
|  | Other HCW | 0.67166 | 0.06841 | 3865 | -9.8178 | <.001 |
| Nurse | Nurse assistant | 0.58553 | 0.06407 | 3865 | -9.1386 | <.001 |
|  | Other HCW | 0.0241 | 0.05422 | 3865 | -0.4444 | 1.000 |
| Nurse assistant | Other HCW | -0.56143 | 0.06824 | 3865 | 8.2276 | <.001 |
| **Collective responsibility** | | | | | | |
| MD | Biomedical | 0.061128 | 0.05457 | 3865 | -1.1202 | 1.000 |
|  | Nurse | 0.48839 | 0.04293 | 3865 | -11.3774 | <.001 |
|  | Nurse assistant | 0.988582 | 0.05443 | 3865 | -18.1634 | <.001 |
|  | Other HCW | 0.495216 | 0.04693 | 3865 | -10.5531 | <.001 |
| Biomedical | Nurse | 0.427262 | 0.05189 | 3865 | -8.234 | <.001 |
|  | Nurse assistant | 0.927454 | 0.06174 | 3865 | -15.0212 | <.001 |
|  | Other HCW | 0.434088 | 0.05524 | 3865 | -7.8576 | <.001 |
| Nurse | Nurse assistant | 0.500192 | 0.05174 | 3865 | -9.6675 | <.001 |
|  | Other HCW | 0.006827 | 0.04378 | 3865 | -0.1559 | 1.000 |
| Nurse assistant | Other HCW | -0.493365 | 0.0551 | 3865 | 8.9535 | <.001 |
| **Favourable environment** | | | | | | |
| MD | Biomedical | 0.07476 | 0.0631 | 3865 | -1.185 | 1.000 |
|  | Nurse | 1.11324 | 0.04964 | 3865 | -22.427 | <.001 |
|  | Nurse assistant | 1.41308 | 0.06294 | 3865 | -22.452 | <.001 |
|  | Other HCW | 0.78628 | 0.05426 | 3865 | -14.49 | <.001 |
| Biomedical | Nurse | 1.03849 | 0.06 | 3865 | -17.307 | <.001 |
|  | Nurse assistant | 1.33833 | 0.0714 | 3865 | -18.744 | <.001 |
|  | Other HCW | 0.71153 | 0.06388 | 3865 | -11.138 | <.001 |
| Nurse | Nurse assistant | 0.29984 | 0.05983 | 3865 | -5.011 | <.001 |
|  | Other HCW | -0.32696 | 0.05063 | 3865 | 6.458 | <.001 |
| Nurse assistant | Other HCW | -0.6268 | 0.06372 | 3865 | 9.837 | <.001 |

## Second-order latent structure

### Definition of the latent factor

#### Confirmatory factor analysis

All indicators significantly contribute to the definition of the latent factor but Convenience show a loading under 0.30 indicating that it participate poorly to the definition of the second order latent factor.

| **Factor** | **Indicator** | **Estimate** | **SE** | **Z** | **p** | **Stand. Estimate** |
| --- | --- | --- | --- | --- | --- | --- |
| VR | Confidence system | 0.8808 | 0.01968 | 44.75 | < .001 | 0.5902 |
|  | Confidence vaccine | 0.8841 | 0.01476 | 59.92 | < .001 | 0.7571 |
|  | High convenience | 0.2687 | 0.01431 | 18.78 | < .001 | **0.2687** |
|  | Low complacency | 0.9403 | 0.01977 | 47.56 | < .001 | 0.6208 |
|  | High BRB | 1.0742 | 0.01468 | 73.16 | < .001 | 0.8512 |
|  | Coll. responsibility | 0.8173 | 0.01213 | 67.39 | < .001 | 0.8173 |
|  | Favourable environment | 0.7447 | 0.01575 | 47.29 | < .001 | 0.6174 |

The goodness of fit indices do reach the thresholds to indicate a good fit of the theoretical model in our population.

|  | **CFI** | **TLI** | **SRMR** | **RMSEA** | | | **Chi²/dF** | **AIC** |
| --- | --- | --- | --- | --- | --- | --- | --- | --- |
|  |  |  |  |  | 90% CI | |  |  |
|  | *>0.90* | *>0.90* | *<0.08* | *<0.07* | *Lower* | *Upper* | *<5* |  |
| Second order | 0.980 | 0.968 | 0.023 | 0.063 | 0.056 | 0.069 | 21.485 | 105589 |

Although Wheaton *et al*’s relative/normed chi-square (Chi2/dF) minimises the impact of sample size on the model, in the CappVaCov database sample size is too large (n> 5000). In French Healthcare workers, a second order latent variable is demonstrated.

#### Second-order latent structure internal consistency

| **Internal consistency – 2^nd^ order** | **McDonald’s ώ** |
| --- | --- |
| French healthcare workers | 0.8391 |

Reliability analysis demonstrates a good internal consistency of the second-order latent structure.

#### Second-order latent score estimation

Mean score per subpopulation:

| **French HCW** | **Vaccine readiness** |
| --- | --- |
| Overall | 0.00 |
| MD | 0.626 |
| Other biomed.^2^ | 0.465 |
| Nurse | -0.187 |
| Nurse assistant | -0.768 |
| Other HCW^1^ | -0.200 |
| ^1,^ for example radio manipulator, laboratory technician, psychologist, other scientific support functions  ^2^ include pharmacist, midwife, biologist, dentist | |


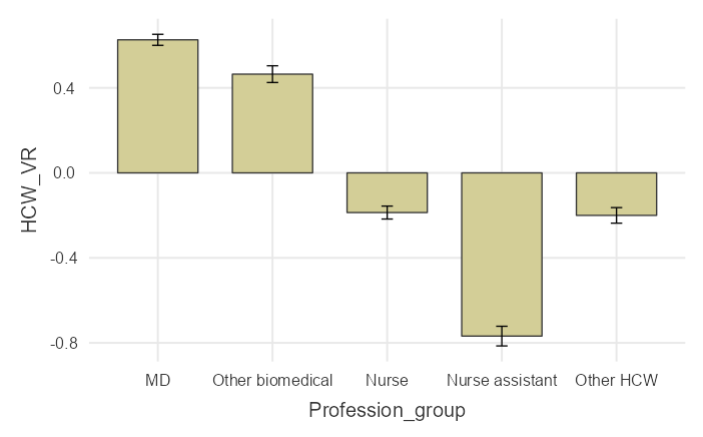


Per groups mean comparison show significant differences (F(4, 3865) = 216.5; p<0.001; ή² = 0.1831) and post-hoc analysis demonstrate significant differences between the professional.

| **French HCW** | | **Mean Difference** | **SE** | **dF** | **t** | **p_bonferroni_** |
| --- | --- | --- | --- | --- | --- | --- |
| MD | Biomedical | 0.162 | 0.055 | 3865.000 | -2.951 | 0.026 |
|  | Nurse | 0.813 | 0.043 | 3865.000 | -18.873 | < .001 |
|  | Nurse assistant | 1.394 | 0.055 | 3865.000 | -25.530 | < .001 |
|  | Other HCW | 0.826 | 0.047 | 3865.000 | -17.547 | < .001 |
| Biomedical | Nurse | 0.651 | 0.052 | 3865.000 | -12.510 | < .001 |
|  | Nurse assistant | 1.233 | 0.062 | 3865.000 | -19.897 | < .001 |
|  | Other HCW | 0.665 | 0.055 | 3865.000 | -11.990 | < .001 |
| Nurse | Nurse assistant | 0.581 | 0.052 | 3865.000 | -11.199 | < .001 |
|  | Other HCW | 0.013 | 0.044 | 3865.000 | -0.303 | 0.998 |
| Nurse assistant | Other HCW | -0.568 | 0.055 | 3865.000 | 10.274 | < .001 |

### Comparison with a 5 C theoretical model

#### Definition of a non-nested 5 C model

We followed the same procedure as for the seven latent factors antecedents to obtain a fitted model with five latent factors antecedents. This 5 C concurrent model (CM) is not nested to the 7 C. The aim is to propose an unconstrained 5 C model to give it every chance of outperforming our proposed 7 C model.

*Script:*

| HCW.5c.1 = '  HCW_confidence_vaccine =~ ZAEfear + ZKgentech + ZKAEreport + ZKAEsurveyEU + ZKAEsixmonth + Zconsideco + ZKskipcontrol  HCW_convenience =~ Zdiffaccess + ZKtwodoses  HCW_complacency =~ Zseriousform + ZKepisevere + ZKlunchrisk + ZKtouchrisk + ZKbloodrisk + ZKeventrisk + ZKobeserisk + ZKagerisk + ZKlongsymp + ZKfiftyrisk + ZKVEflu + Zworry  HCW_calculation =~ Zmorebenef + ZKBRifRF  HCW_coll_responsibility =~ Zcollectstop + ZKblocktrans  '  HCW.5c.2 = '  HCW_confidence_vaccine =~ ZAEfear + ZKgentech + ZKAEsurveyEU + ZKAEsixmonth + Zconsideco + ZKskipcontrol  HCW_convenience =~ Zdiffaccess  HCW_complacency =~ ZKepisevere + ZKlunchrisk + ZKeventrisk + ZKVEflu + Zworry  HCW_calculation =~ Zmorebenef + ZKBRifRF  HCW_coll_responsibility =~ Zcollectstop  '  HCW.5c.3 = '  HCW_confidence_vaccine =~ ZAEfear + ZKgentech + ZKAEsurveyEU + ZKAEsixmonth + Zconsideco + ZKskipcontrol  HCW_convenience =~ Zdiffaccess  HCW_complacency =~ ZKepisevere + ZKlunchrisk + ZKeventrisk + ZKVEflu + Zworry  HCW_calculation =~ Zmorebenef + ZKBRifRF  HCW_coll_responsibility =~ Zcollectstop  ZKlunchrisk ~~ ZKeventrisk  '  HCW.5c.4 = '  HCW_confidence_vaccine =~ ZAEfear + ZKgentech + ZKAEsurveyEU + ZKAEsixmonth + Zconsideco + ZKskipcontrol  HCW_convenience =~ Zdiffaccess  HCW_complacency =~ ZKepisevere + ZKVEflu + Zworry  HCW_calculation =~ Zmorebenef + ZKBRifRF  HCW_coll_responsibility =~ Zcollectstop' |
| --- |

The goodness of fit indices do reach the thresholds to indicate a good fit of the theoretical model in our population.

| **HCW** | **CFI** | **TLI** | **SRMR** | **RMSEA** | | | **Chi²/dF** | **AIC** |
| --- | --- | --- | --- | --- | --- | --- | --- | --- |
|  |  |  |  |  | 90% CI | |  |  |
|  | *>0.90* | *>0.90* | *<0.08* | *<0.07* | *Lower* | *Upper* | *<5* |  |
| Initial model | 0.793 | 0.766 | 0.053 | 0.055 | 0.054 | 0.056 | 16.85 | 355357 |
| #2 | 0.893 | 0.864 | 0.049 | 0.065 | 0.063 | 0.068 | 23.15 | 207640 |
| #3 | 0.919 | 0.895 | 0.042 | 0.057 | 0.055 | 0.060 | 18.06 | 207206 |
| 5 C model | 0.925 | 0.900 | 0.041 | 0.063 | 0.060 | 0.066 | 21.60 | 178620 |

Although Wheaton *et al*’s relative/normed chi-square (Chi2/dF) minimises the impact of sample size on the model, in the CappVaCov database sample size is too large (n> 5000). A five latent factor structure is defined in our population.

#### Vuong’s test for model selection

We performed Vuong’s test (Vuong, 1989) with package Performance (Lüdecke et al., 2021) run with R Studio, version 3.6, run under R (R Core Team, 2019), version 4.1.

*Script:*

| fit1=lm(Mean_VR ~ HCW_distrust_system +  HCW_distrust_vaccine +  HCW_convenience +  HCW_high_complacency +  HCW_low_BRB +  HCW_selfcentred_vision +  HCW_social_conformism, data = HCW_v2)  fit2=lm(Mean_VR~ CM_confidence_vaccine +  CM_convenience +  CM_high_complacency +  CM_low_BRB +  CM_selfcentred, data = HCW_v2)  test_vuong(fit2, fit1) |
| --- |

The Vuong’s test consist of two tests:

1. The Test of Distinguishability (the Omega2 [*Ώ²*] column and its associated p-value) indicates whether or not the models can possibly be distinguished on the basis of the observed data. If its p-value is significant, it means the models are distinguishable;
2. The Robust Likelihood Test (the LR column and its associated p-value) indicates whether each model fits better than the reference model.

| **Model** | **Distinguishability** | | **Robust Likelihood Test** | |
| --- | --- | --- | --- | --- |
|  | *Ώ²* | *p-value* | *LR value* | *p-value* |
| 5 C |  |  |  |  |
| 7 C | 0.71 | <0.001 | -520.35 | <0.001 |

The two models are distinguishable and the 7 C model fits better than the 5 C model to our data.

### Comparison within the 7 C model

**Objective**: based on the 7 C model, to demonstrate that addition of antecedents of vaccine readiness improve the definition of the dependent variable.

*Script:*

| fit7C.HCW=lm(HCW_VR ~ HCW_distrust_system +  HCW_distrust_vaccine +  HCW_convenience +  HCW_high_complacency +  HCW_low_BRB +  HCW_selfcentred_vision +  HCW_social_conformism, data = HCW_v2)  fit6C.HCW=lm(HCW_VR ~ HCW_distrust_system +  HCW_distrust_vaccine +  HCW_convenience +  HCW_high_complacency +  HCW_low_BRB +  HCW_selfcentred_vision, data = HCW_v2)  fit6.2C.HCW=lm(HCW_VR ~ HCW_distrust_vaccine +  HCW_convenience +  HCW_high_complacency +  HCW_low_BRB +  HCW_selfcentred_vision +  HCW_social_conformism, data = HCW_v2)  fit5C.HCW=lm(HCW_VR ~ HCW_distrust_vaccine +  HCW_convenience +  HCW_high_complacency +  HCW_low_BRB +  HCW_selfcentred_vision, data = HCW_v2)  test_wald(fit5C.HCW, fit6C.HCW, fit7C.HCW)  test_wald(fit5C.HCW, fit6.2C.HCW, fit7C.HCW) |
| --- |

| **Model** | **Df** | **Δdf** | **Wald’s test** | **p-value** | **Performance score** |
| --- | --- | --- | --- | --- | --- |
| 5 C | 5228 |  |  |  | 0.00% |
| 6 C.1: with Confidence system | 5227 | 1 | 5.89e+08 | <0.001 | 35.67% |
| 6 C.2: with favourable environment | 5227 | 1 | 3.91e+08 | <0.001 | 22.78% |
| 7 C compared to 6 C.1 | 5226 | 1 | 3.09e+08 | <0.001 | 100% |
| 7 C compared to 6 C.2 | 5226 | 1 | 5.08e+08 | <0.001 |  |

The final model with seven antecedents of vaccine readiness (7 C) is of better performance than a 5 C or a 6 C model.

## Influence of VR and its antecedent on intention to be vaccinated against SARS-COV-2

The statistics were carried out using Jamovi software (The Jamovi Project, 2020), version 2.3 or R Studio, version 3.6, both run under R (R Core Team, 2019), version 4.1, and the libraries Nnet (Ripley & Venables, 2021) and Car (Fox & Weisberg, 2019; fox & Weisberg, 2021).

*Script:*

|  |
| --- |

### MLR with the antecedents of VR

We conducted a multinomial logistic regression on the intention to get vaccinated against SARS-COV-2 with all the antecedents of VR.

#### MLR unadjusted for sociodemographic data

| **Model** | **Components** | **Deviance** | **BIC** | **R²_N_** | **Overall model test** | | |
| --- | --- | --- | --- | --- | --- | --- | --- |
|  |  |  |  |  | *χ²* | *dF* | *p-value* |
| 1 | Confidence vaccine  High convenience  Low complacency  High BRB  Coll. Responsibility | 5460 | 5563 | 0.5422 | 4688 | 10 | < .001 |
| 2 | Confidence system  Favourable env. | 5162 | 5299 | 0.5715 | 4985 | 14 | < .001 |

In comparison with the 5 C model, the addition of the two new antecedents significantly improve the overall model.

| **Comparison** | | **Δχ²** | **Δdf** | **p-value** |
| --- | --- | --- | --- | --- |
| *Model* | *Model* |  |  |  |
| 1 | 2 | 297.8 | 4 | <.001 |

Omnibus likelihood ratio tests were performed on each variable.

| **Predictor** | **χ²** | **dF** | **p** |
| --- | --- | --- | --- |
| Confidence vaccine | 368.654 | 2 | < .001 |
| High convenience | 2.305 | 2 | 0.316 |
| Low complacency | 20.922 | 2 | < .001 |
| High balance benefice risk | 410.219 | 2 | < .001 |
| Collective responsibility | 165.019 | 2 | < .001 |
| Confidence system | 49.006 | 2 | < .001 |
| Favourable environment | 216.588 | 2 | < .001 |

Convenience have no influence on the intention to get vaccinated. These findings are consistent with the period when vaccination was not mandatory and offered to all HCW directly in the hospital.

|  | **Predictor** | **Estimate** | **SE** | **Z** | **p** | **OR** |
| --- | --- | --- | --- | --- | --- | --- |
| Refusal vs. Deliberation | Intercept | -1.481 | 0.093 | -15.886 | < .001 | 0.227 |
|  | **Confidence vaccine** | **-0.370** | **0.064** | **-5.781** | **< .001** | **0.691** |
|  | High convenience | -0.062 | 0.050 | -1.259 | 0.208 | 0.940 |
|  | **Low complacency** | **-0.136** | **0.037** | **-3.673** | **< .001** | **0.873** |
|  | **High balance benefice risk** | **-0.476** | **0.061** | **-7.853** | **< .001** | **0.621** |
|  | **Collective responsibility** | **-0.499** | **0.059** | **-8.405** | **< .001** | **0.607** |
|  | **Confidence system** | **-0.154** | **0.044** | **-3.499** | **< .001** | **0.857** |
|  | **Favourable environment** | **-0.341** | **0.056** | **-6.088** | **< .001** | **0.711** |
| Acceptance vs. deliberation | Intercept | 0.753 | 0.053 | 14.277 | < .001 | 2.124 |
|  | **Confidence vaccine** | **0.906** | **0.059** | **15.372** | **< .001** | **2.474** |
|  | High convenience | 0.031 | 0.054 | 0.568 | 0.570 | 1.031 |
|  | Low complacency | 0.081 | 0.044 | 1.842 | 0.065 | 1.084 |
|  | **High balance benefice risk** | **0.946** | **0.061** | **15.578** | **< .001** | **2.576** |
|  | **Collective responsibility** | **0.522** | **0.073** | **7.176** | **< .001** | **1.686** |
|  | **Confidence system** | **0.198** | **0.039** | **5.025** | **< .001** | **1.219** |
|  | **Favourable environment** | **0.579** | **0.052** | **11.199** | **< .001** | **1.785** |

#### MLR adjusted for sociodemographic variables

| **Model** | **Components** | **Deviance** | **BIC** | **R²_N_** | **Overall model test** | | |
| --- | --- | --- | --- | --- | --- | --- | --- |
|  |  |  |  |  | *χ²* | *dF* | *p-value* |
| 1 | Confidence vaccine  High convenience  Low complacency  High BRB  Coll. Responsibility | 3964 | 4063 | 0.5507 | 3522 | 10 | <0.001 |
| 2 | Confidence system  Favourable env. | 3740 | 3872 | 0.5803 | 3746 | 14 | <0.001 |
| 3 | Gender  Age  Profession | 3708 | 3956 | 0.5845 | 3778 | 28 | <0.001 |

In comparison with the 5 C model, the addition of the two new antecedents significantly improve the overall model.

| **Comparison** | | **Δχ²** | **Δdf** | **p-value** |
| --- | --- | --- | --- | --- |
| *Model* | *Model* |  |  |  |
| 1 | 2 | 223.96 | 4 | <0.001 |
| 2 | 3 | 31.63 | 14 | <0.001 |

Omnibus likelihood ratio tests were performed on each variable.

| **Predictor** | **χ²** | **dF** | **p** |
| --- | --- | --- | --- |
| Confidence vaccine | 238.619 | 2 | <.001 |
| High convenience | 1.836 | 2 | 0.399 |
| Low complacency | 9.373 | 2 | 0.009 |
| High balance benefice risk | 288.032 | 2 | < .001 |
| Collective responsibility | 157.236 | 2 | < .001 |
| Confidence system | 26.529 | 2 | < .001 |
| Favourable environment | 141.849 | 2 | <.001 |
| Gender | 7.973 | 2 | 0.019 |
| Age | 18.125 | 4 | 0.001 |
| Profession | 5.049 | 8 | 0.752 |

Convenience and Profession have no influence on the intention to get vaccinated. These findings are consistent with the period when vaccination was not mandatory and offered to all HCW directly in the hospital.

|  | **Predictor** | **Estimate** | **SE** | **Z** | **p** | **OR** |
| --- | --- | --- | --- | --- | --- | --- |
| Refusal vs. Deliberation | Intercept | -1.634 | 0.249 | -6.564 | <.001 | 0.195 |
|  | **Confidence vaccine** | **0.421** | **0.077** | **5.472** | **< .001** | **1.523** |
|  | High convenience | 0.078 | 0.059 | 1.334 | 0.182 | 1.082 |
|  | **Low complacency** | **0.111** | **0.043** | **2.562** | **0.010** | **1.117** |
|  | **High balance benefice risk** | **0.436** | **0.072** | **6.066** | **< .001** | **1.547** |
|  | **Collective responsibility** | **0.616** | **0.072** | **8.550** | **< .001** | **1.852** |
|  | **Confidence system** | **0.136** | **0.053** | **2.576** | **0.010** | **1.145** |
|  | **Favourable environment** | **0.364** | **0.067** | **5.421** | **< .001** | **1.439** |
|  | Gender: |  |  |  |  |  |
|  | **Male – Female** | **0.389** | **0.180** | **2.160** | **0.031** | **1.475** |
|  | Age: |  |  |  |  |  |
|  | 35/49 – 18-34 | -0.210 | 0.141 | -1.487 | 0.137 | 0.811 |
|  | 50+ – 18-34 | -0.453 | 0.164 | -2.766 | 0.006 | 0.636 |
|  | Profession: |  |  |  |  |  |
|  | 2 – 1 | 0.382 | 0.306 | 1.247 | 0.212 | 1.466 |
|  | 3 – 1 | 0.316 | 0.236 | 1.339 | 0.181 | 1.371 |
|  | 4 – 1 | 0.299 | 0.256 | 1.166 | 0.244 | 1.348 |
|  | 5 – 1 | 0.241 | 0.244 | 0.989 | 0.323 | 1.273 |
| Acceptance vs. Deliberation | Intercept | 0.605 | 0.182 | 3.330 | <.001 | 1.831 |
|  | **Confidence vaccine** | **-0.863** | **0.072** | **-11.991** | **<.001** | **0.422** |
|  | High convenience | 0.032 | 0.064 | 0.507 | 0.612 | 1.033 |
|  | Low complacency | -0.054 | 0.051 | -1.066 | 0.287 | 0.948 |
|  | **High balance benefice risk** | **-0.963** | **0.072** | **-13.467** | **< .001** | **0.382** |
|  | **Collective responsibility** | **-0.556** | **0.085** | **-6.504** | **< .001** | **0.574** |
|  | **Confidence system** | **-0.174** | **0.047** | **-3.710** | **< .001** | **0.841** |
|  | **Favourable environment** | **-0.546** | **0.062** | **-8.794** | **< .001** | **0.579** |
|  | Gender: |  |  |  |  |  |
|  | **Male – Female** | **0.353** | **0.155** | **2.275** | **0.023** | **1.423** |
|  | Age: |  |  |  |  |  |
|  | 35/49 – 18-34 | 0.006 | 0.145 | 0.044 | 0.965 | 1.006 |
|  | **50+ – 18-34** | **0.332** | **0.157** | **2.116** | **0.034** | **1.394** |
|  | Profession: |  |  |  |  |  |
|  | 2 – 1 | -0.118 | 0.222 | -0.531 | 0.595 | 0.889 |
|  | 3 – 1 | -0.136 | 0.179 | -0.763 | 0.446 | 0.873 |
|  | 4 – 1 | 0.132 | 0.232 | 0.567 | 0.571 | 1.141 |
|  | 5 – 1 | -0.084 | 0.190 | -0.439 | 0.661 | 0.920 |

### MLR with VR

We conducted a multinomial logistic regression on the intention to get vaccinated against SARS-COV-2 with VR.

#### MLR unadjusted for sociodemographic variables

| **Model** | **Components** | **Deviance** | **BIC** | **R²_N_** | **Overall model test** | | |
| --- | --- | --- | --- | --- | --- | --- | --- |
|  |  |  |  |  | *χ²* | *dF* | *p-value* |
| 1 | Vaccine readiness | 5324 | 5358 | 0.5557 | 4824 | 2 | < .001 |

Omnibus likelihood ratio tests were performed on each variable.

| **Predictor** | **χ²** | **dF** | **p** |
| --- | --- | --- | --- |
| Vaccine readiness score | 4824 | 2 | < .001 |

|  | **Predictor** | **Estimate** | **SE** | **Z** | **p** | **OR** |
| --- | --- | --- | --- | --- | --- | --- |
| Refusal vs. Deliberation | Intercept | -1.3838 | 0.0833 | -16.61 | < .001 | 0.2506 |
|  | Vaccine readiness score | -1.6847 | 0.07862 | -21.43 | < .001 | 0.1855 |
| Acceptance vs. Deliberation | Intercept | 0.595 | 0.04918 | 12.1 | < .001 | 1.813 |
|  | Vaccine readiness score | 2.9327 | 0.09316 | 31.48 | < .001 | 18.7782 |

#### MLR adjusted for sociodemographic variables

| **Model** | **Components** | **Deviance** | **BIC** | **R²_N_** | **Overall model test** | | |
| --- | --- | --- | --- | --- | --- | --- | --- |
|  |  |  |  |  | *χ²* | *dF* | *p-value* |
| 1 | Vaccine readiness | 3868 | 3901 | 0.5634 | 3618 | 2 | < .001 |
| 2 | Gender  Age  Profession | 3811 | 3960 | 0.571 | 3675 | 16 | < .001 |

| **Comparison** | | **Δχ²** | **Δdf** | **p-value** |
| --- | --- | --- | --- | --- |
| *Model* | *Model* |  |  |  |
| 1 | 2 | 56.82 | 14 | < .001 |

Omnibus likelihood ratio tests were performed on each variable.

| **Predictor** | **χ²** | **dF** | **p** |
| --- | --- | --- | --- |
| Vaccine readiness score | 2956.453 | 2 | < .001 |
| Gender | 11.672 | 2 | 0.003 |
| Age | 30.94 | 4 | < .001 |
| Profession | 9.854 | 8 | 0.275 |

Profession has no influence on the intention to get vaccinated.

|  | **Predictor** | **Estimate** | **SE** | **Z** | **p** | **OR** |
| --- | --- | --- | --- | --- | --- | --- |
| Refusal vs. Deliberation | Intercept | -1.599 | 0.245 | -6.536 | < .001 | 0.202 |
|  | **Vaccine readiness score** | **-1.768** | **0.097** | **-18.182** | **< .001** | **0.171** |
|  | Gender: |  |  |  |  |  |
|  | **Male – Female** | **0.390** | **0.177** | **2.198** | **0.028** | **1.476** |
|  | Age: |  |  |  |  |  |
|  | 35/49 – 18-34 | -0.238 | 0.140 | -1.703 | 0.089 | 0.788 |
|  | **50+ – 18-34** | **-0.500** | **0.161** | **-3.101** | **0.002** | **0.606** |
|  | Profession: |  |  |  |  |  |
|  | 2 – 1 | 0.406 | 0.306 | 1.329 | 0.184 | 1.501 |
|  | 3 – 1 | 0.447 | 0.232 | 1.927 | 0.054 | 1.563 |
|  | 4 – 1 | 0.414 | 0.252 | 1.645 | 0.100 | 1.512 |
|  | 5 – 1 | 0.281 | 0.241 | 1.164 | 0.244 | 1.324 |
| Acceptance vs. Deliberation | Intercept | 0.477 | 0.174 | 2.732 | 0.006 | 1.610 |
|  | **Vaccine readiness score** | **2.817** | **0.111** | **25.438** | **< .001** | **16.727** |
|  | Gender: |  |  |  |  |  |
|  | **Male – Female** | **0.458** | **0.150** | **3.044** | **0.002** | **1.580** |
|  | Age: |  |  |  |  |  |
|  | 35/49 – 18-34 | 0.060 | 0.141 | 0.426 | 0.670 | 1.062 |
|  | **50+ – 18-34** | **0.505** | **0.152** | **3.321** | **< .001** | **1.656** |
|  | Profession: |  |  |  |  |  |
|  | 2 – 1 | -0.142 | 0.214 | -0.662 | 0.508 | 0.868 |
|  | 3 – 1 | -0.279 | 0.170 | -1.636 | 0.102 | 0.757 |
|  | 4 – 1 | -0.023 | 0.224 | -0.101 | 0.919 | 0.978 |
|  | 5 – 1 | -0.181 | 0.182 | -0.992 | 0.321 | 0.835 |

## References

| Estabrook, R., & Neale, M. (2013). A Comparison of Factor Score Estimation Methods in the Presence of Missing Data: Reliability and an Application to Nicotine Dependence. *Multivariate Behavioral Research*, *48*(1), 1–27. https://doi.org/10.1080/00273171.2012.730072  Fox, J., & Weisberg, S. (2019). *An R companion to applied regression* (Third edition). SAGE.  fox, J., & Weisberg, S. (2021). *car: Companion to Applied Regression* (3.0-12) [R]. https://CRAN.R-project.org/package=car  Hooper, D., Coughlan, J., & Mullen, M. R. (2008). Structural Equation Modelling: Guidelines for Determining Model Fit. *Electronic Journal of Business Research Methods*, *6*(1), 53–60.  Hoyle, R. H. (Ed.). (2012). *Handbook of structural equation modeling*. Guilford Press.  Hu, L., & Bentler, P. M. (1999). Cutoff criteria for fit indexes in covariance structure analysis: Conventional criteria versus new alternatives. *Structural Equation Modeling: A Multidisciplinary Journal*, *6*(1), 1–55. https://doi.org/10.1080/10705519909540118  Lenth, R. V., Buerkner, P., Herve, M., Love, J., Miguez, F., Riebl, H., & Singmann, H. (2022). *emMeans: Estimated Marginal Means, aka Least-Squares Means* (1.7.4-1) [Computer software]. https://CRAN.R-project.org/package=emmeans  Lüdecke, D., Ben-Shachar, M., Patil, I., Waggoner, P., & Makowski, D. (2021). performance: An R Package for Assessment, Comparison and Testing of Statistical Models. *Journal of Open Source Software*, *6*(60), 3139. https://doi.org/10.21105/joss.03139  Oudin Doglioni, D., Pham-Hung D’Alexandry D’Orengiani, A.-L., Galactéros, F., & Gay, M.-C. (2021). Psychometric characteristics of the Revised Illness Perception Questionnaire (IPQ-R) in adults with sickle cell disease. *Health Psychology and Behavioral Medicine*, *10*(1), 60–80. https://doi.org/10.1080/21642850.2021.2016411  R Core Team. (2019). *R: A Language and environment for statistical computing.* (3.6) [Computer software].  Revelle, W. (2022). *Psych: Procedures for Psychological, Psychometric, and Personality Research* (2.2.5) [R package]. Northwestern University. https://CRAN.R-project.org/package=psych  Ripley, B., & Venables, W. (2021). *nnet: Feed-Forward Neural Networks and Multinomial Log-Linear Models* (7.3-17) [R]. https://cran.r-project.org/web/packages/nnet/index.html  Rosseel, Y. (2012). **lavaan**: An *R* Package for Structural Equation Modeling. *Journal of Statistical Software*, *48*(2). https://doi.org/10.18637/jss.v048.i02  Rosseel, Y., & Jorgensen, T. D. (2019). *Package ‘lavaan’* (0.6-10) [R]. https://cran.r-project.org/web/packages/lavaan/lavaan.pdf  Steiger, J. H. (2002). When constraints interact: A caution about reference variables, identification constraints, and scale dependencies in structural equation modeling. *Psychological Methods*, *7*(2), 210–227. https://doi.org/10.1037/1082-989X.7.2.210  Steiger, J. H. (2007). Understanding the limitations of global fit assessment in structural equation modeling. *Personality and Individual Differences*, *42*(5), 893–898. https://doi.org/10.1016/j.paid.2006.09.017  The Jamovi Project. (2020). *Jamovi* (1.2) [Computer software].  Vuong, Q. H. (1989). Likelihood Ratio Tests for Model Selection and Non-Nested Hypotheses. *Econometrica*, *57*(2), 307. https://doi.org/10.2307/1912557  Wheaton, B., Muthen, B., Alwin, D. F., & Summers, G. F. (1977). Assessing Reliability and Stability in Panel Models. *Sociological Methodology*, *8*, 84–136. https://doi.org/10.2307/270754 |
| --- |

# Supplemental material 3: Parents – Human Papilloma Virus (HPV)

## First-order latent structure

**Objective**: to identify a latent factorial organisation referring to the antecedents of vaccine readiness (VR) with a covariance relationship indicating the possible existence of a common formative second-order factor. As assessing the statistical significance of the loading for each indicator is important, we fixed the variance of the factor (Hoyle, 2012; Steiger, 2002) and freed all indicators. To estimate the factor score, we used the regression or exact method with Bartlett’s correction for bias in factor means (Estabrook & Neale, 2013). The score produced have a mean of zero and a free standard deviation. It is understood that each antecedent should be defined by, at least, one item as the objective is not to validate a questionnaire but a theoretical model.

We used the two-index presentation strategy (Hu & Bentler, 1999) including: the maximum likelihood (ML)‐based standardised root mean squared residual (SRMR) with a cut-off value under .08; and, the root mean square of approximation (RMSEA) with 90% confidence intervals and cut-off value close to .07 (Steiger, 2007). We added 3 indicators of goodness of fit (Hooper et al., 2008): the Wheaton *et al.*’s relative/normed chi square with a range from 2 to 5 (Wheaton et al., 1977); the comparative fit index (CFI) with a value greater than .90; and, the non-normed fit index (NNFI) also known as the Tucker-Lewis index (TLI) with a .90 threshold (Oudin Doglioni et al., 2021).

| **Indices** | **Presentation** | **Cut off** | **Reference** |
| --- | --- | --- | --- |
| SRMR | Absolute fit indice which determine how well an *a priori* model fits the sample data. The SRMR are the square root of the difference between the residuals of the sample covariance matrix and the hypothesised covariance model. | <0.08 | Hu & Bentler, 1999 |
| RMSEA | The RMSEA tells us how well the model, with unknown but optimally chosen parameter estimates would fit the population covariance matrix. The RMSEA favours parsimony in that it will choose the model with the lesser number of parameters. | <0.07 | Steiger, 2007 |
| relative/normed chi square | The Chi-Square value is the traditional measure for evaluating overall model fit and, assesses the magnitude of the discrepancy between the sample and fitted covariances matrices. Because the Chi-Square statistic is in essence a statistical significance test, it is sensitive to sample size which means that the Chi-Square statistic nearly always rejects the model when large samples are used. One example of a statistic that minimises the impact of sample size on the model Chi-Square is Wheaton *et al*’s relative/normed chi-square (χ2/dF). | [2;5] | Wheaton et al., 1977 |
| CFI | The Comparative Fit Index is a revised form of the NFI which takes into account sample size that performs well even when sample size is small. | >0.90 | Hu & Bentler, 1999 |
| NNFI/TLI | The Non-Normed Fit Index assesses the model by comparing the χ2 value of the model to the χ2 of the null model. | >0.90 | Hooper et al.; 2008 |

The statistics were carried out using Jamovi software (The Jamovi Project, 2020), version 2.3 or R Studio, version 3.6, both run under R (R Core Team, 2021), version 4.1, and the Laavan (Rosseel, 2012; Rosseel & Jorgensen, 2019), the Psych (Revelle, 2022), the Car libraries (Fox & Weisberg, 2019; fox & Weisberg, 2021) and the Emmeans library (Lenth et al., 2022).

The script used in Jamovi and R Studio are indicative. In particular, word written in italic refers to generic terms that must be adapted to the specific context.

*Script:*

| Model = ‘*factor* =~ *variable of interest*’  Model.fit = cfa(Model, data = *data*, missing = “ML”)  summary(Model.fit, fit.measures = TRUE)  Model.fit.predict = lavpredict(Model.fit, type = “lv”, method = “Bartlett”, label = TRUE, std.lv = TRUE) |
| --- |
| jmv::cfa(data = data,  factors = list(  list(label="*factor name*", vars=c("*variables of interest*")),  resCov = NULL,  stdEst = TRUE,  fitMeasures = c("cfi", "tli", "rmsea", "srmr", "aic")  mi = TRUE) |

### Exploration of each antecedent

#### Convenience

All indicators are significantly participating in the definition of the latent factor. But almost half of the indicators loaded poorly to the factor (in bold).

| **Factor** | **Indicator** | **Estimate** | **SE** | **Z** | **p** | **Stand. Estimate** |
| --- | --- | --- | --- | --- | --- | --- |
| Convenience | **Zc2_k1_T** | **0.2422** | **0.02808** | **8.627** | **<.001** | **0.2423** |
|  | **Zc2_k2_T** | **0.2148** | **0.02832** | **7.586** | **<.001** | **0.2149** |
|  | **Zc2_k3_T** | **0.2531** | **0.02789** | **9.072** | **<.001** | **0.2531** |
|  | Zc2_a1_T | -0.5007 | 0.02742 | -18.259 | <.001 | -0.5008 |
|  | Zc2_a2 | -0.6413 | 0.02583 | -24.827 | <.001 | -0.6414 |
|  | Zc2_a3 | -0.7193 | 0.02642 | -27.222 | <.001 | -0.7193 |
|  | Zc2_a4 | -0.4983 | 0.02678 | -18.609 | <.001 | -0.4977 |

As a consequence, the goodness of fit indices are not indicating that the latent factor is well defined.

| **Antecedent** | **CFI** | **TLI** | **SRMR** | **RMSEA** | | | **Chi²/dF** | **AIC** |
| --- | --- | --- | --- | --- | --- | --- | --- | --- |
|  |  |  |  |  | 90% CI | |  |  |
|  | *>0.90* | *>0.90* | *<0.08* | *<0.07* | *Lower* | *Upper* | *<5* |  |
| Convenience | **0.8719** | **0.8078** | 0.04965 | 0.08626 | 0.07589 | **0.09707** | 14.28 | 34098 |

#### Complacency

All indicators are significantly participating in the definition of the latent factor, but two are loading poorly on the latent factor (in bold).

| **Factor** | **Indicator** | **Estimate** | **SE** | **Z** | **p** | **Stand. Estimate** |
| --- | --- | --- | --- | --- | --- | --- |
| Complacency | **Zc3_k1_T** | **0.1855** | **0.02748** | **6.751** | **<.001** | **0.1856** |
|  | Zc3_k2_T | 0.331 | 0.02712 | 12.207 | <.001 | 0.3311 |
|  | **Zc3_k3_T** | **0.2858** | **0.0272** | **10.506** | **<.001** | **0.2859** |
|  | Zc3_a1_T | -0.7669 | 0.02958 | -25.924 | <.001 | -0.7671 |
|  | Zc3_a2 | -0.7382 | 0.02913 | -25.343 | <.001 | -0.7383 |

As a result, the goodness of fit indices are not above their thresholds, indicating that the latent factor is misspecified.

| **Antecedent** | **CFI** | **TLI** | **SRMR** | **RMSEA** | | | **Chi²/dF** | **AIC** |
| --- | --- | --- | --- | --- | --- | --- | --- | --- |
|  |  |  |  |  | 90% CI | |  |  |
|  | *>0.90* | *>0.90* | *<0.08* | *<0.07* | *Lower* | *Upper* | *<5* |  |
| Complacency | 0.9388 | **0.8775** | 0.03950 | 0.08481 | 0.06773 | **0.1031** | 13.83 | 24296 |

### Exploration of the theoretical 7 C model

#### Definition of the seven latent factors structure

##### Initial input (model 1)

All indicators are significantly participating in the definition of their respective latent factor. Indicators with loading (standard estimate) higher than 0.30 are of enough contribution. Indicators with loading under .30 should be removed in a Model 2 (in bold).

| **Factor** | **Indicator** | **Estimate** | **SE** | **Z** | **p** | **Stand. Estimate** |
| --- | --- | --- | --- | --- | --- | --- |
| Confidence system | Zvaccination_college | 1.000 | 0.016 | 62.881 | <.001 | 1.000 |
| Confidence vaccine | **Zc1_k1_T** | **0.164** | **0.028** | **5.755** | **< .001** | **0.164** |
|  | Zc1_a1 | 0.588 | 0.061 | 9.573 | < .001 | 0.588 |
| Convenience | Zc2_k1_T | 0.306 | 0.025 | 12.286 | < .001 | 0.306 |
|  | **Zc2_k2_T** | **0.297** | **0.025** | **11.921** | **< .001** | **0.297** |
|  | **Zc2_k3_T** | **0.276** | **0.025** | **11.087** | **< .001** | **0.276** |
|  | Zc2_a1_T | -0.867 | 0.021 | -41.642 | < .001 | -0.866 |
|  | Zc2_a2 | -0.384 | 0.025 | -15.544 | < .001 | -0.384 |
|  | Zc2_a3 | -0.396 | 0.025 | -16.009 | < .001 | -0.396 |
|  | **Zc2_a4** | **-0.287** | **0.025** | **-11.331** | **<.001** | **-0.288** |
| Complacency | **Zc3_k1_T** | **0.174** | **0.025** | **7.059** | **<.001** | **0.175** |
|  | Zc3_k2_T | 0.316 | 0.024 | 13.032 | <.001 | 0.316 |
|  | **Zc3_k3_T** | **0.268** | **0.024** | **10.973** | **<.001** | **0.269** |
|  | Zc3_a1_T | -0.829 | 0.021 | -40.114 | <.001 | -0.829 |
|  | Zc3_a2 | -0.689 | 0.022 | -31.486 | <.001 | -0.689 |
| Calculation | Zc4_k1_T | 0.624 | 0.024 | 26.478 | <.001 | 0.624 |
|  | Zc4_a1 | 0.792 | 0.024 | 33.630 | <.001 | 0.792 |
| Coll. responsibility | Zc5_k1_T | 0.384 | 0.026 | 15.036 | <.001 | 0.384 |
|  | Zc5_a1 | 0.712 | 0.030 | 24.043 | <.001 | 0.712 |
| Social conformism | **Zc6_k1_T** | **0.081** | **0.077** | **1.059** | **0.290** | **0.081** |
|  | **Zc6_a1** | **0.158** | **0.144** | **1.098** | **0.272** | **0.158** |

As objective is to validate a theoretical model, not a questionnaire, antecedents should contain at least one item. When all items are of low loading, the higher must be kept. Accordingly, Social conformism will be defined solely by 1 item.

All factors do not covary. In particular, Social conformism that is misspecified covary with no other factor.

| **Factor** | | **Estimate** |  | **SE** | **Z** | **p** | **Stand. Estimate** |
| --- | --- | --- | --- | --- | --- | --- | --- |
| Confidence system | Confidence system | 1.000 | ᵃ |  |  |  |  |
|  | Confidence vaccine | 0.290 |  | 0.046 | 6.314 | <.001 | 0.290 |
|  | Complacency | 0.169 |  | 0.026 | 6.464 | <.001 | 0.169 |
|  | Convenience | 0.291 |  | 0.025 | 11.633 | <.001 | 0.291 |
|  | Calculation | 0.311 |  | 0.026 | 11.906 | <.001 | 0.311 |
|  | Coll. responsibility | 0.348 |  | 0.031 | 11.331 | <.001 | 0.348 |
|  | **Social conformism** | **0.681** |  | **0.625** | **1.090** | **0.276** | **0.681** |
| Confidence vaccine | Confidence vaccine | 1.000 | ᵃ |  |  |  |  |
|  | Complacency | 1.030 |  | 0.102 | 10.072 | <.001 | 1.030 |
|  | Convenience | 1.035 |  | 0.102 | 10.098 | <.001 | 1.035 |
|  | Calculation | 0.970 |  | 0.099 | 9.754 | <.001 | 0.970 |
|  | Coll. responsibility | 1.023 |  | 0.108 | 9.442 | <.001 | 1.023 |
|  | **Social conformism** | **2.871** |  | **2.604** | **1.103** | **0.270** | **2.871** |
| Complacency | Complacency | 1.000 | ᵃ |  |  |  |  |
|  | Convenience | 0.946 |  | 0.014 | 67.543 | <.001 | 0.946 |
|  | Calculation | 0.773 |  | 0.022 | 35.809 | <.001 | 0.773 |
|  | Coll. responsibility | 0.889 |  | 0.031 | 28.525 | <.001 | 0.889 |
|  | **Social conformism** | **2.023** |  | **1.821** | **1.111** | **0.267** | **2.023** |
| Convenience | Convenience | 1.000 | ᵃ |  |  |  |  |
|  | Calculation | 0.885 |  | 0.019 | 47.554 | <.001 | 0.885 |
|  | Coll. responsibility | 1.015 |  | 0.032 | 31.689 | <.001 | 1.015 |
|  | **Social conformism** | **2.259** |  | **2.033** | **1.111** | **0.267** | **2.259** |
| Calculation | Calculation | 1.000 | ᵃ |  |  |  |  |
|  | Coll. responsibility | 0.923 |  | 0.035 | 26.103 | <.001 | 0.923 |
|  | **Social conformism** | **2.168** |  | **1.952** | **1.111** | **0.267** | **2.168** |
| Coll. responsibility | Coll. responsibility | 1.000 | ᵃ |  |  |  |  |
|  | **Social conformism** | **2.604** |  | **2.345** | **1.110** | **0.267** | **2.604** |
| ᵃ fixed parameter |  |  |  |  |  |  |  |

The goodness of fit indices do not reach the thresholds to indicate a good fit of the theoretical model in our population.

|  | **CFI** | **TLI** | **SRMR** | **RMSEA** | | | **Chi²/dF** | **AIC** |
| --- | --- | --- | --- | --- | --- | --- | --- | --- |
|  |  |  |  |  | 90% CI | |  |  |
|  | *>0.90* | *>0.90* | *<0.08* | *<0.07* | *Lower* | *Upper* | *<5* |  |
| Initial model | **0.8306** | **0.7896** | 0.05563 | 0.06717 | 0.06427 | **0.07011** | 9.917 | 99312 |

To improve the fit, low loading indicators were removed in a second model.

##### Removal of non-contributory indicators (model 2)

All indicators contribute to the definition of their respective latent factors and loading are all above 0.30.

| **Factor** | **Indicator** | **Estimate** | **SE** | **Z** | **p** | **Stand. Estimate** |
| --- | --- | --- | --- | --- | --- | --- |
| Confidence system | Zvaccination_college | 1.000 | 0.016 | 62.880 | <.001 | 1.000 |
| Confidence vaccine | Zc1_a1 | 1.000 | 0.017 | 59.590 | < .001 | 1.000 |
| Convenience | Zc2_a1_T | 0.886 | 0.022 | 39.470 | < .001 | 0.886 |
|  | Zc2_a2 | 0.364 | 0.025 | 14.730 | < .001 | 0.364 |
|  | Zc2_a3 | 0.372 | 0.025 | 15.080 | < .001 | 0.372 |
| Complacency | Zc3_a1_T | -0.839 | 0.021 | -40.560 | <.001 | -0.839 |
|  | Zc3_a2 | -0.683 | 0.022 | -31.180 | <.001 | -0.683 |
| Calculation | Zc4_k1_T | 0.616 | 0.024 | 26.030 | <.001 | 0.616 |
|  | Zc4_a1 | 0.802 | 0.024 | 33.900 | <.001 | 0.803 |
| Coll. Responsibility | Zc5_k1_T | 0.376 | 0.026 | 14.740 | <.001 | 0.376 |
|  | Zc5_a1 | 0.726 | 0.030 | 24.210 | <.001 | 0.726 |
| Social conformism | Zc6_a1 | 1.000 | 0.017 | 59.580 | <.001 | 1.000 |

All factors covary suggesting that they refer to a common latent second order.

| **Factor** | | **Estimate** |  | **SE** | **Z** | **p** | **Stand. Estimate** |
| --- | --- | --- | --- | --- | --- | --- | --- |
| Confidence system | Confidence system | 1.000 | ᵃ |  |  |  |  |
|  | Confidence vaccine | 0.183 |  | 0.023 | 7.987 | <.001 | 0.183 |
|  | Complacency | -0.191 |  | 0.026 | -7.435 | <.001 | -0.191 |
|  | Convenience | 0.295 |  | 0.025 | 11.830 | <.001 | 0.295 |
|  | Calculation | 0.311 |  | 0.026 | 11.945 | <.001 | 0.311 |
|  | Coll. responsibility | 0.344 |  | 0.030 | 11.311 | <.001 | 0.344 |
|  | Social conformism | 0.118 |  | 0.023 | 5.071 | <.001 | 0.118 |
| Confidence vaccine | Confidence vaccine | 1.000 | ᵃ |  |  |  |  |
|  | Complacency | -0.607 |  | 0.019 | -31.682 | <.001 | -0.607 |
|  | Convenience | 0.612 |  | 0.019 | 32.810 | <.001 | 0.612 |
|  | Calculation | 0.560 |  | 0.022 | 25.690 | <.001 | 0.560 |
|  | Coll. responsibility | 0.594 |  | 0.029 | 20.406 | <.001 | 0.594 |
|  | Social conformism | 0.270 |  | 0.022 | 12.243 | <.001 | 0.270 |
| Complacency | Complacency | 1.000 | ᵃ |  |  |  |  |
|  | Convenience | -0.940 |  | 0.017 | -55.702 | <.001 | -0.940 |
|  | Calculation | -0.755 |  | 0.023 | -33.239 | <.001 | -0.755 |
|  | Coll. responsibility | -0.872 |  | 0.032 | -26.976 | <.001 | -0.872 |
|  | Social conformism | -0.319 |  | 0.025 | -12.952 | <.001 | -0.319 |
| Convenience | Convenience | 1.000 | ᵃ |  |  |  |  |
|  | Calculation | 0.875 |  | 0.019 | 46.287 | <.001 | 0.875 |
|  | Coll. responsibility | 0.994 |  | 0.032 | 31.332 | <.001 | 0.994 |
|  | Social conformism | 0.357 |  | 0.024 | 14.792 | <.001 | 0.357 |
| Calculation | Calculation | 1.000 | ᵃ |  |  |  |  |
|  | Coll. responsibility | 0.902 |  | 0.035 | 25.488 | <.001 | 0.902 |
|  | Social conformism | 0.348 |  | 0.026 | 13.618 | <.001 | 0.348 |
| Coll. responsibility | Coll. responsibility | 1.000 | ᵃ |  |  |  |  |
|  | Social conformism | 0.417 |  | 0.030 | 13.982 | <.001 | 0.417 |
| ᵃ fixed parameter |  |  |  |  |  |  |  |

TLI does not reach the threshold to indicate a good fit of the theoretical model in our population.

|  | **CFI** | **TLI** | **SRMR** | **RMSEA** | | | **Chi²/dF** | **AIC** |
| --- | --- | --- | --- | --- | --- | --- | --- | --- |
|  |  |  |  |  | 90% CI | |  |  |
|  | *>0.90* | *>0.90* | *<0.08* | *<0.07* | *Lower* | *Upper* | *<5* |  |
| Model 2 | 0.9227 | **0.8717** | 0.04540 | 0.07724 | 0.07180 | **0.08281** | 12.79 | 59583 |

Modification indices suggest adding covariance between indicators from the same latent factors which was done in a Model 3:

- **Zc2_a3** (‘I find it difficult to discuss HPV vaccination with a health professional (doctor, nurse, etc.).’) | **Zc2_a2** (‘I find it difficult to talk about HPV vaccination with my relative.’) which refers to the difficulties to speak about HPV vaccination (MI = 334.701).

This addition was made in a third model.

##### Addition of covariance between indicators (model 3)

*Script:*

| Model = ‘*factor* =~ *variable of interest*  *indicator~~indicator*’  Model.fit = cfa(Model, data = *data*, missing = “ML”)  summary(Model.fit, fit.measures = TRUE)  modindices(Model.fit, sort = TRUE, maximum.number = 5)  Model.fit.predict = lavpredict(Model.fit, type = “lv”, method = “Bartlett”, label = TRUE, std.lv = TRUE) |
| --- |
| jmv::cfa(data = data,  factors = list(  list(label="*factor name*", vars=c("*variables of interest*")),  resCov = list (  list (i_n_ = "*variables of interest*")),  stdEst = TRUE,  fitMeasures = c("cfi", "tli", "rmsea", "srmr", "aic")  mi = TRUE) |

All indicators significantly contribute to the definition of their respective latent factor

| **Factor** | **Indicator** | **Estimate** | **SE** | **Z** | **p** | **Stand. Estimate** |
| --- | --- | --- | --- | --- | --- | --- |
| Confidence system | Zvaccination_college | 1.000 | 0.016 | 62.880 | <.001 | 1.000 |
| Confidence vaccine | Zc1_a1 | 1.000 | 0.017 | 59.600 | < .001 | 1.000 |
| Convenience | Zc2_a1_T | 0.902 | 0.027 | 33.140 | < .001 | 0.902 |
|  | Zc2_a2 | 0.336 | 0.025 | 13.560 | < .001 | 0.336 |
|  | Zc2_a3 | 0.345 | 0.025 | 14.050 | <.001 | 0.346 |
| Complacency | Zc3_a1_T | 0.843 | 0.021 | 40.360 | <.001 | 0.844 |
|  | Zc3_a2 | 0.679 | 0.022 | 30.950 | <.001 | 0.679 |
| Calculation | Zc4_k1_T | 0.613 | 0.024 | 25.870 | <.001 | 0.613 |
|  | Zc4_a1 | 0.806 | 0.024 | 34.020 | <.001 | 0.806 |
| Coll responsibility | Zc5_k1_T | 0.373 | 0.026 | 14.630 | <.001 | 0.373 |
|  | Zc5_a1 | 0.731 | 0.030 | 24.230 | <.001 | 0.731 |
| Social conformism | Zc6_a1 | 1.000 | 0.017 | 59.590 | <.001 | 1.000 |

All factors covary suggesting the existence of a second-order latent factor.

| **Factor** | | **Estimate** |  | **SE** | **Z** | **p** | **Stand. Estimate** |
| --- | --- | --- | --- | --- | --- | --- | --- |
| Confidence system | Confidence system | 1 | ᵃ |  |  |  |  |
|  | Confidence vaccine | 0.1825 |  | 0.02285 | 7.985 | <.001 | 0.1825 |
|  | Complacency | -0.1944 |  | 0.02535 | -7.669 | <.001 | -0.1944 |
|  | Convenience | -0.2993 |  | 0.02491 | -12.015 | <.001 | -0.2993 |
|  | Calculation | 0.31 |  | 0.02594 | 11.951 | <.001 | 0.3100 |
|  | Coll. responsibility | 0.3417 |  | 0.03026 | 11.291 | <.001 | 0.3417 |
|  | Social conformism | 0.1183 |  | 0.02332 | 5.07 | <.001 | 0.1183 |
| Confidence vaccine | Confidence vaccine | 1 | ᵃ |  |  |  |  |
|  | Complacency | -0.6013 |  | 0.02168 | -27.743 | <.001 | -0.6013 |
|  | Convenience | -0.6109 |  | 0.01883 | -32.446 | <.001 | -0.6109 |
|  | Calculation | 0.5588 |  | 0.02182 | 25.611 | <.001 | 0.5588 |
|  | Coll. responsibility | 0.5905 |  | 0.0291 | 20.287 | <.001 | 0.5905 |
|  | Social conformism | 0.2694 |  | 0.02201 | 12.24 | <.001 | 0.2694 |
| Complacency | Complacency | 1 | ᵃ |  |  |  |  |
|  | Convenience | 0.9288 |  | 0.02363 | 39.307 | <.001 | 0.9288 |
|  | Calculation | -0.7452 |  | 0.02599 | -28.668 | <.001 | -0.7452 |
|  | Coll. responsibility | -0.8556 |  | 0.03552 | -24.087 | <.001 | -0.8556 |
|  | Social conformism | -0.3115 |  | 0.02513 | -12.398 | <.001 | -0.3115 |
| Convenience | Convenience | 1 | ᵃ |  |  |  |  |
|  | Calculation | -0.8731 |  | 0.0191 | -45.702 | <.001 | -0.8731 |
|  | Coll. responsibility | -0.9858 |  | 0.03167 | -31.127 | <.001 | -0.9858 |
|  | Social conformism | -0.3541 |  | 0.02422 | -14.618 | <.001 | -0.3541 |
| Calculation | Calculation | 1 | ᵃ |  |  |  |  |
|  | Coll. responsibility | 0.8943 |  | 0.03546 | 25.222 | <.001 | 0.8943 |
|  | Social conformism | 0.3478 |  | 0.02553 | 13.625 | <.001 | 0.3478 |
| Coll. responsibility | Coll. responsibility | 1 | ᵃ |  |  |  |  |
|  | Social conformism | 0.4145 |  | 0.02969 | 13.961 | <.001 | 0.4145 |
| ᵃ fixed parameter |  |  |  |  |  |  |  |

The goodness of fit indices do reach the thresholds to indicate a good fit of the theoretical model in our population.

|  | **CFI** | **TLI** | **SRMR** | **RMSEA** | | | **Chi²/dF** | **AIC** |
| --- | --- | --- | --- | --- | --- | --- | --- | --- |
|  |  |  |  |  | 90% CI | |  |  |
|  | *>0.90* | *>0.90* | *<0.08* | *<0.07* | *Lower* | *Upper* | *<5* |  |
| Model 3 | 0.9802 | 0.9627 | 0.02463 | 0.04463 | 0.03811 | 0.05138 | 4.937 | 54306 |

In adults from the general population, a latent organisation on seven first-order factors is demonstrated.

##### CFA synthesis

Definition of the seven latent factors structure shows constant improvement of the fit to the data.

|  | **Adults (PrevHPV database)** | | | | | | | |
| --- | --- | --- | --- | --- | --- | --- | --- | --- |
| **Model** | **CFI** | **TLI** | **SRMR** | **RMSEA** | | | **Chi²/dF** | **AIC** |
|  |  |  |  |  | 90% CI | |  |  |
|  | *>0.90* | *>0.90* | *<0.08* | *<0.07* | *Lower* | *Upper* | *<5* |  |
| Initial | 0.8306 | 0.7896 | 0.05563 | 0.06717 | 0.06427 | 0.07011 | 9.917 | 99312 |
| #2 | 0.9227 | 0.8717 | 0.04540 | 0.07724 | 0.07180 | 0.08281 | 12.79 | 59583 |
| final | 0.9802 | 0.9627 | 0.02463 | 0.04463 | 0.03811 | 0.05138 | 4.937 | 54306 |

#### First-order latent structure internal consistency

*Script:*

| jmv::reliability(data = data,  vars = vars(*variables of interest*),  alphaScale = FALSE,  omegaScale = TRUE) |
| --- |

| **Internal consistency – 1^st^ order** | **McDonald’s ώ** |
| --- | --- |
| General population – Adults | 0.8512 |

Reliability analysis demonstrates a good internal consistency of the first-order latent structure.

### Latent factors scores estimation

In order to respect the meaning of vaccine readiness, reverse coding must be applied:

| High VR when | High | Confidence system |  | Confidence system |
| --- | --- | --- | --- | --- |
|  | High | Confidence vaccine |  | Confidence vaccine |
|  | High | Convenience | Reverse | High convenience |
|  | Low | Complacency | Reverse | Low complacency |
|  | High | Calculation |  | High benefice risk balance (BRB) |
|  | High | Collective responsibility |  | Collective responsibility |
|  | High | Social conformism |  | Favourable environment |

Mean score per subpopulation:

| **Adults** | **Confidence system** | **Confidence vaccine** | **High convenience** | **Low complacency** | **High BRB** | **Collective responsibility** | **Favourable environment** |
| --- | --- | --- | --- | --- | --- | --- | --- |
| Overall | -1.72e−4 | 0.005 | 0.006 | 0.008 | 0.002 | -0.001 | 0.003 |
| Independent | -0.040 | 0.016 | -0.038 | 0.036 | 0.034 | -0.060 | 0.020 |
| Executive | 0.191 | 0.194 | 0.233 | 0.290 | 0.439 | 0.328 | 0.042 |
| Technician | 0.055 | 0.103 | 0.138 | 0.175 | 0.228 | 0.213 | 0.010 |
| Employee | -0.085 | -0.080 | -0.061 | -0.156 | -0.238 | -0.140 | 0.024 |
| Worker | -0.121 | -0.227 | -0.461 | -0.552 | -0.522 | -0.849 | -0.194 |
| Other | -0.118 | -0.209 | -0.282 | -0.222 | -0.403 | -0.327 | -0.067 |

Kiviat diagram figure specific patterns of vaccine readiness with larger circle expressing higher overall readiness.

Per subpopulations mean comparison show significant differences for all the factors.

*Script:*

| jmv::ANOVA(formula = `*factor of interest*` ~ Profession_group,  data = data,  postHoc = ~ Profession_group,  postHocCorr = "bonf",  emMeans = ~ Profession_group,  emmTables = TRUE) |
| --- |

| **Adults** | **ANOVA** | | | **P-value** | **ή²** |
| --- | --- | --- | --- | --- | --- |
|  | Df1 | Df2 | F test |  |  |
| Confidence system | 5 | 1971 | 5.464 | <0.001 | 0.0137^*^ |
| Confidence vaccine | 5 | 1778 | 7.816 | <0.001 | 0.0215^*^ |
| High convenience | 5 | 1769 | 11.26 | <0.001 | 0.0308^*^ |
| Low complacency | 5 | 1769 | 14.76 | <0.001 | 0.0400^*^ |
| High benefice risk balance | 5 | 1778 | 29.22 | <0.001 | 0.0759^**^ |
| Collective responsibility | 5 | 1778 | 17.43 | <0.001 | 0.0467^*^ |
| Favourable environment | 5 | 1769 | 0.9385 | 0.455 | 0.0026^*^ |
| Effect size, ή²: ^***^large - ^**^medium - ^*^small | | | | | |

Post-hoc analysis with a Bonferroni correction tends to show differences related to the level of education.

| **Adults** | | **Mean Difference** | **SE** | **dF** | **t** | **p_bonferroni_** |
| --- | --- | --- | --- | --- | --- | --- |
| **Confidence system** | | | | | | |
| Independent | Executive | 0.231 | 0.109 | 1971 | -2.124 | 0.508 |
|  | Technician | 0.096 | 0.108 | 1971 | -0.884 | 1.000 |
|  | Employee | -0.045 | 0.106 | 1971 | 0.426 | 1.000 |
|  | Worker | -0.081 | 0.149 | 1971 | 0.543 | 1.000 |
|  | Other | -0.078 | 0.112 | 1971 | 0.697 | 1.000 |
| Executive | Technician | -0.135 | 0.068 | 1971 | 1.994 | 0.694 |
|  | Employee | -0.276 | 0.063 | 1971 | 4.366 | <.001 |
|  | Worker | -0.312 | 0.123 | 1971 | 2.546 | 0.165 |
|  | Other | -0.309 | 0.073 | 1971 | 4.245 | <.001 |
| Technician | Employee | -0.141 | 0.062 | 1971 | 2.256 | 0.362 |
|  | Worker | -0.177 | 0.122 | 1971 | 1.446 | 1.000 |
|  | Other | -0.174 | 0.072 | 1971 | 2.410 | 0.241 |
| Employee | Worker | -0.036 | 0.120 | 1971 | 0.300 | 1.000 |
|  | Other | -0.033 | 0.068 | 1971 | 0.484 | 1.000 |
| Worker | Other | 0.003 | 0.125 | 1971 | -0.024 | 1.000 |
| **Confidence vaccine** | | | | | | |
| Independent | Executive | 0.178 | 0.119 | 1778 | -1.492 | 1.000 |
|  | Technician | 0.087 | 0.119 | 1778 | -0.732 | 1.000 |
|  | Employee | -0.096 | 0.117 | 1778 | 0.824 | 1.000 |
|  | Worker | -0.243 | 0.164 | 1778 | 1.479 | 1.000 |
|  | Other | -0.225 | 0.124 | 1778 | 1.814 | 1.000 |
| Executive | Technician | -0.091 | 0.069 | 1778 | 1.321 | 1.000 |
|  | Employee | -0.274 | 0.065 | 1778 | 4.201 | <.001 |
|  | Worker | -0.421 | 0.133 | 1778 | 3.164 | 0.024 |
|  | Other | -0.402 | 0.078 | 1778 | 5.191 | <.001 |
| Technician | Employee | -0.183 | 0.064 | 1778 | 2.850 | 0.066 |
|  | Worker | -0.330 | 0.133 | 1778 | 2.490 | 0.193 |
|  | Other | -0.312 | 0.077 | 1778 | 4.063 | <.001 |
| Employee | Worker | -0.147 | 0.131 | 1778 | 1.126 | 1.000 |
|  | Other | -0.129 | 0.073 | 1778 | 1.754 | 1.000 |
| Worker | Other | 0.018 | 0.137 | 1778 | -0.134 | 1.000 |
| **High convenience** | | | | | | |
| Independent | Executive | 0.270 | 0.131 | 1769 | -2.061 | 0.592 |
|  | Technician | 0.175 | 0.131 | 1769 | -1.343 | 1.000 |
|  | Employee | -0.022 | 0.128 | 1769 | 0.174 | 1.000 |
|  | Worker | -0.418 | 0.180 | 1769 | 2.315 | 0.311 |
|  | Other | -0.241 | 0.136 | 1769 | 1.767 | 1.000 |
| Executive | Technician | -0.095 | 0.075 | 1769 | 1.259 | 1.000 |
|  | Employee | -0.293 | 0.071 | 1769 | 4.095 | <.001 |
|  | Worker | -0.688 | 0.146 | 1769 | 4.724 | <.001 |
|  | Other | -0.511 | 0.085 | 1769 | 6.003 | <.001 |
| Technician | Employee | -0.198 | 0.070 | 1769 | 2.808 | 0.076 |
|  | Worker | -0.593 | 0.145 | 1769 | 4.087 | <.001 |
|  | Other | -0.416 | 0.084 | 1769 | 4.940 | <.001 |
| Employee | Worker | -0.395 | 0.143 | 1769 | 2.763 | 0.087 |
|  | Other | -0.219 | 0.081 | 1769 | 2.707 | 0.103 |
| Worker | Other | 0.177 | 0.150 | 1769 | -1.175 | 1.000 |
| **Low complacency** | | | | | | |
| Independent | Executive | 0.254 | 0.136 | 1769 | -1.873 | 0.918 |
|  | Technician | 0.139 | 0.135 | 1769 | -1.026 | 1.000 |
|  | Employee | -0.192 | 0.133 | 1769 | 1.448 | 1.000 |
|  | Worker | -0.588 | 0.187 | 1769 | 3.147 | 0.025 |
|  | Other | -0.258 | 0.141 | 1769 | 1.826 | 1.000 |
| Executive | Technician | -0.116 | 0.078 | 1769 | 1.481 | 1.000 |
|  | Employee | -0.447 | 0.074 | 1769 | 6.038 | <.001 |
|  | Worker | -0.842 | 0.151 | 1769 | 5.587 | <.001 |
|  | Other | -0.512 | 0.088 | 1769 | 5.808 | <.001 |
| Technician | Employee | -0.331 | 0.073 | 1769 | 4.543 | <.001 |
|  | Worker | -0.727 | 0.150 | 1769 | 4.837 | <.001 |
|  | Other | -0.397 | 0.087 | 1769 | 4.545 | <.001 |
| Employee | Worker | -0.396 | 0.148 | 1769 | 2.670 | 0.115 |
|  | Other | -0.065 | 0.084 | 1769 | 0.783 | 1.000 |
| Worker | Other | 0.330 | 0.156 | 1769 | -2.120 | 0.512 |
| **High benefit-risk balance** | | | | | | |
| Independent | Executive | 0.406 | 0.138 | 1778 | -2.938 | 0.050 |
|  | Technician | 0.195 | 0.138 | 1778 | -1.419 | 1.000 |
|  | Employee | -0.271 | 0.135 | 1778 | 2.005 | 0.677 |
|  | Worker | -0.556 | 0.191 | 1778 | 2.918 | 0.054 |
|  | Other | -0.437 | 0.144 | 1778 | 3.038 | 0.036 |
| Executive | Technician | -0.211 | 0.080 | 1778 | 2.641 | 0.125 |
|  | Employee | -0.677 | 0.076 | 1778 | 8.956 | <.001 |
|  | Worker | -0.962 | 0.154 | 1778 | 6.238 | <.001 |
|  | Other | -0.843 | 0.090 | 1778 | 9.367 | <.001 |
| Technician | Employee | -0.466 | 0.074 | 1778 | 6.265 | <.001 |
|  | Worker | -0.752 | 0.154 | 1778 | 4.889 | <.001 |
|  | Other | -0.632 | 0.089 | 1778 | 7.101 | <.001 |
| Employee | Worker | -0.285 | 0.152 | 1778 | 1.883 | 0.898 |
|  | Other | -0.166 | 0.085 | 1778 | 1.943 | 0.782 |
| Worker | Other | 0.120 | 0.159 | 1778 | -0.753 | 1.000 |
| **Collective responsibility** | | | | | | |
| Independent | Executive | 0.388 | 0.157 | 1778 | -2.466 | 0.206 |
|  | Technician | 0.273 | 0.156 | 1778 | -1.746 | 1.000 |
|  | Employee | -0.080 | 0.154 | 1778 | 0.518 | 1.000 |
|  | Worker | -0.788 | 0.217 | 1778 | 3.632 | 0.004 |
|  | Other | -0.266 | 0.163 | 1778 | 1.627 | 1.000 |
| Executive | Technician | -0.114 | 0.091 | 1778 | 1.261 | 1.000 |
|  | Employee | -0.467 | 0.086 | 1778 | 5.437 | <.001 |
|  | Worker | -1.175 | 0.175 | 1778 | 6.698 | <.001 |
|  | Other | -0.653 | 0.102 | 1778 | 6.389 | <.001 |
| Technician | Employee | -0.353 | 0.085 | 1778 | 4.171 | <.001 |
|  | Worker | -1.061 | 0.175 | 1778 | 6.069 | <.001 |
|  | Other | -0.539 | 0.101 | 1778 | 5.329 | <.001 |
| Employee | Worker | -0.708 | 0.172 | 1778 | 4.108 | <.001 |
|  | Other | -0.186 | 0.097 | 1778 | 1.922 | 0.822 |
| Worker | Other | 0.522 | 0.181 | 1778 | -2.882 | 0.060 |
| **Favourable environment** | | | | | | |
| Independent | Executive | 0.022 | 0.121 | 1769 | -0.178 | 1.000 |
|  | Technician | -0.010 | 0.121 | 1769 | 0.082 | 1.000 |
|  | Employee | 0.004 | 0.119 | 1769 | -0.035 | 1.000 |
|  | Worker | -0.214 | 0.167 | 1769 | 1.282 | 1.000 |
|  | Other | -0.087 | 0.126 | 1769 | 0.692 | 1.000 |
| Executive | Technician | -0.032 | 0.070 | 1769 | 0.452 | 1.000 |
|  | Employee | -0.017 | 0.066 | 1769 | 0.264 | 1.000 |
|  | Worker | -0.235 | 0.135 | 1769 | 1.749 | 1.000 |
|  | Other | -0.109 | 0.079 | 1769 | 1.382 | 1.000 |
| Technician | Employee | 0.014 | 0.065 | 1769 | -0.217 | 1.000 |
|  | Worker | -0.204 | 0.134 | 1769 | 1.520 | 1.000 |
|  | Other | -0.077 | 0.078 | 1769 | 0.993 | 1.000 |
| Employee | Worker | -0.218 | 0.132 | 1769 | 1.648 | 1.000 |
|  | Other | -0.091 | 0.075 | 1769 | 1.224 | 1.000 |
| Worker | Other | 0.127 | 0.139 | 1769 | -0.910 | 1.000 |

## Second-order latent structure

*Script:*

| Model = ‘*factor* =~ *variable of interest*’  Model.fit = cfa(Model, data = *data*, missing = “ML”)  summary(Model.fit, fit.measures = TRUE)  Model.fit.predict = lavpredict(Model.fit, type = “lv”, method = “Bartlett”, label = TRUE, std.lv = TRUE) |
| --- |
| jmv::cfa(data = data,  factors = list(  list(label="*factor name*", vars=c("*variables of interest*")),  resCov = NULL,  stdEst = TRUE,  fitMeasures = c("cfi", "tli", "rmsea", "srmr", "aic")  mi = TRUE) |

### Definition of the latent factor

#### Confirmatory factor analysis

All indicators significantly contribute to the definition of the latent factor but Confidence in the system show a loading under 0.30 indicating that it participates poorly to the definition of the second order latent factor.

| **Factor** | **Indicator** | **Estimate** | **SE** | **Z** | **p** | **Stand. Estimate** |
| --- | --- | --- | --- | --- | --- | --- |
|  | *Confidence system (****item A****)* | *0.101* | *0.025* | *4.022* | *<.001* | ***0.101*** |
| VR | Confidence system (**item B**) | 0.291 | 0.024 | 12.04 | < .001 | **0.291** |
|  | Confidence vaccine | 0.622 | 0.022 | 27.98 | < .001 | 0.624 |
|  | High convenience | 0.900 | 0.022 | 40.20 | < .001 | 0.814 |
|  | Low complacency | 1.012 | 0.022 | 45.73 | < .001 | 0.886 |
|  | High BRB | 0.873 | 0.025 | 34.64 | < .001 | 0.734 |
|  | Collective responsibility | 0.986 | 0.028 | 34.99 | < .001 | 0.739 |
|  | Favourable environment | 0.373 | 0.024 | 15.43 | < .001 | 0.373 |

The goodness of fit indices do reach the thresholds to indicate a good fit of the theoretical model in our population.

|  | **CFI** | **TLI** | **SRMR** | **RMSEA** | | | **Chi²/dF** | **AIC** |
| --- | --- | --- | --- | --- | --- | --- | --- | --- |
|  |  |  |  |  | 90% CI | |  |  |
|  | *>0.90* | *>0.90* | *<0.08* | *<0.07* | *Lower* | *Upper* | *<5* |  |
| Second order | 0.982 | 0.974 | 0.021 | 0.055 | 0.045 | 0.066 | 6.988 | 33746 |

Although Wheaton *et al*’s relative/normed chi-square (Chi2/dF) minimises the impact of sample size on the model, in the PrevHPV database sample size is too large (n> 2000). In adults from the general population, a second order latent variable is demonstrated.

#### Second-order latent structure internal consistency

| **Internal consistency – 2^nd^ order** | **McDonald’s ώ** |
| --- | --- |
| General population – Adults | 0.8396 |

Reliability analysis demonstrates a good internal consistency of the second-order latent structure.

#### Second-order latent score estimation

Mean score per subpopulation:

| **Adults** | **Vaccine readiness** |
| --- | --- |
| Overall | 0.004 |
| Independent | -0.004 |
| Executive | 0.321 |
| Technician | 0.183 |
| Employee | -0.147 |
| Worker | -0.568 |
| Other | -0.303 |


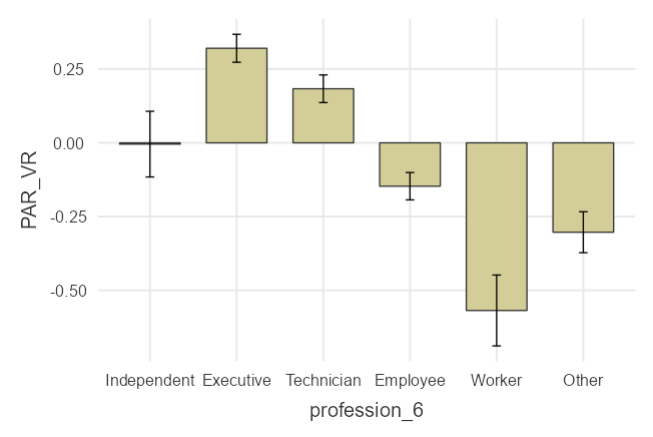


Per groups mean comparison show significant differences (F(5, 1778) = 21.37; p<0.001; ή² = 0.057) and post-hoc analysis demonstrate few significant differences between the professional.

| **Adults** | | **Mean Difference** | **SE** | **dF** | **t** | **p_bonferroni_** |
| --- | --- | --- | --- | --- | --- | --- |
| Independent | Executive | 0.325 | 0 | 1778 | 2.622 | 0.132 |
|  | Technician | 0.188 | 0 | 1778 | 1.522 | 1.000 |
|  | Employee | -0.143 | 0 | 1778 | -1.177 | 1.000 |
|  | Worker | -0.564 | 0 | 1778 | -3.299 | 0.015 |
|  | Other | -0.299 | 0 | 1778 | -2.319 | 0.308 |
| Executive | Technician | -0.137 | 0 | 1778 | -1.917 | 0.83 |
|  | Employee | -0.468 | 0 | 1778 | -6.9 | < .001 |
|  | Worker | -0.889 | 0 | 1778 | -6.426 | < .001 |
|  | Other | -0.624 | 0 | 1778 | -7.735 | < .001 |
| Technician | Employee | -0.330 | 0 | 1778 | -4.952 | < .001 |
|  | Worker | -0.752 | 0 | 1778 | -5.454 | < .001 |
|  | Other | -0.486 | 0 | 1778 | -6.1 | < .001 |
| Employee | Worker | -0.421 | 0 | 1778 | -3.101 | 0.029 |
|  | Other | -0.156 | 0 | 1778 | -2.044 | 0.616 |
| Worker | Other | 0.265 | 0 | 1778 | 1.859 | 0.949 |

### Comparison with a 5 C theoretical model

#### Definition of a non-nested 5C model

We followed the same procedure as for the seven latent factors antecedents to obtain a fitted model with five latent factors antecedents. This 5 C concurrent model (CM) is not nested to the 7 C. The aim is to propose an unconstrained 5 C model to give it every chance of outperforming our proposed 7 C model.

*Script:*

| PAR.5C.1='  CM_confidence_vaccine =~ Zc1_k1_T + Zc1_a1  CM_convenience =~ Zc2_k1_T + Zc2_k2_T + Zc2_k3_T + Zc2_a1_T + Zc2_a2 + Zc2_a3 + Zc2_a4  CM_compalcency =~ Zc3_k1_T + Zc3_k2_T + Zc3_k3_T + Zc3_a1_T + Zc3_a2  CM_calculation =~ Zc4_k1_T + Zc4_a1  CM_coll_responsibility =~ Zc5_k1_T + Zc5_a1  '  PAR.5C.2='  CM_confidence_vaccine =~ Zc1_a1  CM_convenience =~ Zc2_a1_T + Zc2_a2 + Zc2_a3  CM_compalcency =~ Zc3_k2_T + Zc3_a1_T + Zc3_a2  CM_calculation =~ Zc4_k1_T + Zc4_a1  CM_coll_responsibility =~ Zc5_k1_T + Zc5_a1  '  PAR.5C.3='  CM_confidence_vaccine =~ Zc1_a1  CM_convenience =~ Zc2_a1_T + Zc2_a2 + Zc2_a3  CM_compalcency =~ Zc3_k2_T + Zc3_a1_T + Zc3_a2  CM_calculation =~ Zc4_k1_T + Zc4_a1  CM_coll_responsibility =~ Zc5_k1_T + Zc5_a1  Zc2_a2 ~~ Zc2_a3' |
| --- |

The goodness of fit indices do reach the thresholds to indicate a good fit of the theoretical model in our population.

| **Adults** | **CFI** | **TLI** | **SRMR** | **RMSEA** | | | **Chi²/dF** | **AIC** |
| --- | --- | --- | --- | --- | --- | --- | --- | --- |
|  |  |  |  |  | 90% CI | |  |  |
|  | *>0.90* | *>0.90* | *<0.08* | *<0.07* | *Lower* | *Upper* | *<5* |  |
| Initial model | 0.835 | 0.798 | 0.059 | 0.078 | 0.075 | 0.082 | 11.93 | 84068 |
| #2 | 0.921 | 0.877 | 0.050 | 0.092 | 0.085 | 0.099 | 16.08 | 49342 |
| 5 C model | 0.975 | 0.960 | 0.028 | 0.052 | 0.046 | 0.060 | 4.90 | 48928 |

A five latent factors structure is defined in our population in a non-nested concurrent model.

#### Vuong’s test for model selection

We performed Vuong’s test (Vuong, 1989) with package Performance (Lüdecke et al., 2021) run with R Studio, version 3.6, run under R (R Core Team, 2021), version 4.1.

*Script:*

| fit.1.par = lm(Mean_VR ~  CM_Distrust_vaccine +  CM_low_convenience +  CM_high_compalcency +  CM_low_BRB +  CM_selfcentred, data = PAR_v2)  fit.2.par = lm(PAR_VR ~  PAR_distrust_system_2 +  PAR_distrust_vaccine_2 +  PAR_low_convenience_2 +  PAR_high_complacency_2 +  PAR_low_BRB_2 +  PAR_Selfcentred_2 +  PAR_sceptic_environment_2, data = PAR_v2)  test_vuong(fit.1.par, fit.2.par) |
| --- |

The Vuong’s test consist of two tests:

1. The Test of Distinguishability (the Omega2 [*Ώ²*] column and its associated p-value) indicates whether or not the models can possibly be distinguished on the basis of the observed data. If its p-value is significant, it means the models are distinguishable;
2. The Robust Likelihood Test (the LR column and its associated p-value) indicates whether each model fits better than the reference model, set as the 5 C model.

| **Model** | **Distinguishability** | | **Robust Likelihood Test** | |
| --- | --- | --- | --- | --- |
|  | *Ώ²* | *p-value* | *LR value* | *p-value* |
| 5 C |  |  |  |  |
| 7 C | 0.95 | <0.001 | -82.14 | <0.001 |

The two models are distinguishable and the 7 C model fits better than the 5 C model to our data.

### Comparison within the 7 C model

**Objective**: based on the 7 C model, to demonstrate that addition of antecedents of vaccine readiness improve the definition of the dependent variable.

*Script:*

| fit7C.PAR=lm(PAR_VR_2 ~  PAR_distrust_system_2 +  PAR_distrust_vaccine_2 +  PAR_low_convenience_2 +  PAR_high_complacency_2 +  PAR_low_BRB_2 +  PAR_selfcentred_vision +  PAR_sceptic_environment_2, data = PAR_v2)  PAR_sceptic_environment_2, data = PAR_v2)  fit6C.PAR=lm(PAR_VR_2 ~  PAR_distrust_system_2 +  PAR_distrust_vaccine_2 +  PAR_low_convenience_2 +  PAR_high_complacency_2 +  PAR_low_BRB_2 +  PAR_selfcentred_vision, data = PAR_v2)  fit6.2C.PAR=lm(PAR_VR_2 ~  PAR_distrust_vaccine_2 +  PAR_low_convenience_2 +  PAR_high_complacency_2 +  PAR_low_BRB_2 +  PAR_selfcentred_vision +  PAR_sceptic_environment_2, data = PAR_v2)  fit5C.PAR=lm(PAR_VR_2 ~  PAR_distrust_vaccine_2 +  PAR_low_convenience_2 +  PAR_high_complacency_2 +  PAR_low_BRB_2 +  PAR_selfcentred_vision, data = PAR_v2)  test_wald(fit5C.PAR, fit6C.PAR, fit7C.PAR)  test_wald(fit5C.PAR, fit6.2C.PAR, fit7C.PAR) |
| --- |

| **Nested models** | **Df** | **Δdf** | **Wald’s test** | **p-value** | **Performance score** |
| --- | --- | --- | --- | --- | --- |
| 5 C | 1769 |  |  |  | 0.00% |
| 6 C.1: with Confidence system | 1768 | 1 | 7.78e+29 | <0.001 | 0.06% |
| 6 C.2: with favourable environment | 1768 | 1 | 1.15e+30 | <0.001 | 68.98% |
| 7 C compared to 6 C.1 | 1767 | 1 | 1.12e+30 | <0.001 | 100% |
| 7 C compared to 6 C.2 | 1767 | 1 | 7.53e+29 | <0.001 |  |

The final model with seven antecedents of vaccine readiness (7 C) is of better performance than a nested 5 C or a nested 6 C model.

## Influence of VR and its antecedent on intention to be vaccinated against HPV

*Data management:*

| **Name** | **Formula** | **Comments** |
| --- | --- | --- |
| HPV_knowledge | IF(connaissance_HPV = 2  AND (mg_parle_t0_v2 = 2 OR mg_parle_t0_v2 = 3)  AND (mg_vaccin_t0_v2 = 2 OR mg_vaccin_t0_v2 = 3)  THEN (HPV_knowledge = 2)  OTHERWISE (HPV_knowledge = 1) | 1 = knowledge of HPV  2 = no knowledge of HPV |
| PAR_intentionality | IF HPV_knowledge = 2  THEN PAR_intentionality = 1  IF (prochsaska_1 OR Prochaska_2 = 1)  THEN PAR_intentionality = 2  IF (prochsaska_1 OR Prochaska_2 = 2)  THE PAR_intentionality = 3  IF (prochsaska_1 OR Prochaska_2 = 3)  THEN PAR_intentionality = 4  IF (prochsaska_1 OR Prochaska_2 = 4)  THEN PAR_intentionality = 5  IF (statut_vaccinal = 2 OR statut_vaccinal = 3 OR statut_vaccinal = 4)  THEN PAR_intentionality = 6 | 1 = ‘ignorance’  2 = ‘precontemplation’  3 = ‘contemplation’  4 = ‘intention’  5 = ‘preparation’  6 = ‘action’ |
| PAR_acceptance | IF (PAR_intentionality = 1 OR PAR_intentionality = 2)  THEN PAR_acceptance = 1  IF PAR_intentionality = 3  THEN PAR_acceptance = 2  OTHERWISE PAR_acceptance = 3 | 1 = ‘refusal’  2 = ‘deliberation’  3 = ‘acceptance’ |

The statistics were carried out using Jamovi software (The Jamovi Project, 2020), version 2.3 or R Studio, version 3.6, both run under R (R Core Team, 2021), version 4.1, and the libraries Nnet (Ripley & Venables, 2021) and Car (Fox & Weisberg, 2019; fox & Weisberg, 2021).

*Script:*

|  |
| --- |

### MLR with the antecedents of VR

We conducted a multinomial logistic regression on the intention to vaccinate their children against HPV with all the antecedents of VR.

#### MLR unadjusted for sociodemographic variables

| **Model** | **Components** | **Deviance** | **BIC** | **R²_N_** | **Overall model test** | | |
| --- | --- | --- | --- | --- | --- | --- | --- |
|  |  |  |  |  | *χ²* | *dF* | *p-value* |
| 1 | Confidence vaccine  High convenience  Low complacency  High BRB  Coll. Responsibility | 1653 | 1743 | 0.4421 | 1020 | 10 | < .001 |
| 2 | Confidence system  Favourable env. | 1636 | 1756 | 0.4487 | 1037 | 14 | < .001 |

In comparison with the 5 C model, the addition of the two new antecedents significantly improve the overall model.

| **Comparison** | | **Δχ²** | **Δdf** | **p-value** |
| --- | --- | --- | --- | --- |
| *Model* | *Model* |  |  |  |
| 1 | 2 | 16.91 | 4 | 0.002 |

Omnibus likelihood ratio tests were performed on each variable.

| **Predictor** | **χ²** | **dF** | **p** |
| --- | --- | --- | --- |
| Confidence vaccine | 3.106 | 2 | 0.212 |
| High convenience | 48.436 | 2 | < .001 |
| Low complacency | 132.956 | 2 | < .001 |
| High BRB | 19.733 | 2 | < .001 |
| Collective responsibility | 4.118 | 2 | 0.128 |
| Confidence system | 0.292 | 2 | 0.864 |
| Favourable environment | 16.652 | 2 | < .001 |

|  | **Predictor** | **Estimate** | **SE** | **Z** | **p** | **OR** |
| --- | --- | --- | --- | --- | --- | --- |
| Refusal vs. Deliberating | Intercept | -1.083 | 0.176 | -6.165 | < .001 | 0.339 |
|  | Confidence vaccine | 0.187 | 0.154 | 1.211 | 0.226 | 1.206 |
|  | High convenience | -0.014 | 0.121 | -0.113 | 0.910 | 0.986 |
|  | Low complacency | -0.164 | 0.128 | -1.274 | 0.203 | 0.849 |
|  | High BRB | 0.149 | 0.126 | 1.181 | 0.237 | 1.161 |
|  | **Collective responsibility** | **-0.175** | **0.087** | **-2.008** | **0.045** | **0.840** |
|  | Confidence system | 0.048 | 0.107 | 0.444 | 0.657 | 1.049 |
|  | Favourable environment | -0.073 | 0.117 | -0.620 | 0.535 | 0.930 |
| Acceptance vs. Deliberating | Intercept | 2.046 | 0.106 | 19.320 | < .001 | **7.735** |
|  | Confidence vaccine | 0.180 | 0.111 | 1.629 | 0.103 | 1.198 |
|  | **High convenience** | **0.731** | **0.111** | **6.555** | **< .001** | **2.076** |
|  | **Low complacency** | **1.185** | **0.121** | **9.756** | **< .001** | **3.270** |
|  | **High BRB** | **0.428** | **0.094** | **4.561** | **< .001** | **1.534** |
|  | Collective responsibility | -0.041 | 0.079 | -0.520 | 0.603 | 0.960 |
|  | Confidence system | 0.039 | 0.091 | 0.430 | 0.667 | 1.040 |
|  | **Favourable environment** | **0.352** | **0.097** | **3.648** | **< .001** | **1.422** |

#### MLR adjusted for sociodemographic variables

| **Model** | **Components** | **Deviance** | **BIC** | **R²_N_** | **Overall model test** | | |
| --- | --- | --- | --- | --- | --- | --- | --- |
|  |  |  |  |  | *χ²* | *dF* | *p-value* |
| 1 | Confidence vaccine  High convenience  Low complacency  High BRB  Coll. Responsibility | 1653 | 1743 | 0.4421 | 1020 | 10 | < .001 |
| 2 | Confidence system  Favourable env. | 1636 | 1756 | 0.4487 | 1037 | 14 | < .001 |
| 3 | Gender  Age  Profession | 1607 | 1861 | 0.4602 | 1067 | 32 | < .001 |

In comparison with the 5 C model, the addition of the two new antecedents significantly improve the overall model.

| **Comparison** | | **Δχ²** | **Δdf** | **p-value** |
| --- | --- | --- | --- | --- |
| *Model* | *Model* |  |  |  |
| 1 | 2 | 16.91 | 4 | 0.002 |
| 2 | 3 | 29.55 | 18 | 0.042 |

Omnibus likelihood ratio tests were performed on each variable.

| **Predictor** | **χ²** | **dF** | **p** |
| --- | --- | --- | --- |
| Confidence vaccine | 3.384 | 2 | 0.184 |
| High convenience | 51.562 | 2 | < .001 |
| Low complacency | 128.657 | 2 | < .001 |
| High BRB | 23.846 | 2 | < .001 |
| Collective responsibility | 3.991 | 2 | 0.136 |
| Confidence system | 0.090 | 2 | 0.956 |
| Favourable environment | 17.041 | 2 | < .001 |
| Gender | 4.023 | 2 | 0.134 |
| Age | 3.480 | 6 | 0.747 |
| Profession | 21.224 | 10 | 0.02 |

|  | **Predictor** | **Estimate** | **SE** | **Z** | **p** | **OR** |
| --- | --- | --- | --- | --- | --- | --- |
| Refusal vs. Deliberating | Intercept | -1.121 | 0.716 | -1.564 | 0.118 | 0.326 |
|  | Confidence vaccine | 0.211 | 0.157 | 1.340 | 0.180 | 1.235 |
|  | High convenience | 0.018 | 0.125 | 0.140 | 0.888 | 1.018 |
|  | Low complacency | -0.157 | 0.130 | -1.202 | 0.230 | 0.855 |
|  | High BRB | 0.189 | 0.129 | 1.464 | 0.143 | 1.208 |
|  | Collective responsibility | -0.169 | 0.088 | -1.919 | 0.055 | 0.844 |
|  | Confidence system | 0.019 | 0.109 | 0.176 | 0.860 | 1.019 |
|  | Favourable environment | -0.081 | 0.120 | -0.681 | 0.496 | 0.922 |
|  | Gender: |  |  |  |  |  |
|  | Male – Female | 0.466 | 0.382 | 1.219 | 0.223 | 1.593 |
|  | Age: |  |  |  |  |  |
|  | 35/44 – under 35 | 0.075 | 0.458 | 0.163 | 0.871 | 1.077 |
|  | 45/54 – under 35 | -0.056 | 0.474 | -0.119 | 0.905 | 0.945 |
|  | 55 and more – under 35 | 0.397 | 0.990 | 0.401 | 0.689 | 1.487 |
|  | Profession: |  |  |  |  |  |
|  | Executive – Independent | -0.087 | 0.589 | -0.148 | 0.882 | 0.917 |
|  | Technician – Independent | -0.317 | 0.586 | -0.541 | 0.588 | 0.728 |
|  | Employee – Independent | 0.072 | 0.561 | 0.128 | 0.898 | 1.075 |
|  | Worker – Independent | -0.516 | 0.718 | -0.718 | 0.473 | 0.597 |
|  | Other – Independent | 0.708 | 0.580 | 1.221 | 0.222 | 2.029 |
| Acceptance vs. Deliberating | Intercept | 2.951 | 0.596 | 4.956 | < .001 | 19.131 |
|  | Confidence vaccine | 0.186 | 0.113 | 1.655 | 0.098 | 1.205 |
|  | **High convenience** | **0.773** | **0.114** | **6.808** | **< .001** | **2.167** |
|  | **Low complacency** | **1.176** | **0.122** | **9.631** | **< .001** | **3.240** |
|  | **High BRB** | **0.486** | **0.097** | **5.026** | **< .001** | **1.626** |
|  | Collective responsibility | -0.008 | 0.081 | -0.105 | 0.916 | 0.992 |
|  | Confidence system | 0.027 | 0.093 | 0.287 | 0.774 | 1.027 |
|  | **Favourable environment** | **0.360** | **0.099** | **3.653** | **< .001** | **1.433** |
|  | Gender: |  |  |  |  |  |
|  | Male – Female | 0.611 | 0.323 | 1.889 | 0.059 | 1.842 |
|  | Age: |  |  |  |  |  |
|  | 35/44 – under 35 | -0.390 | 0.425 | -0.918 | 0.359 | 0.677 |
|  | 45/54 – under 35 | -0.392 | 0.437 | -0.899 | 0.369 | 0.675 |
|  | 55 and more – under 35 | 0.754 | 0.877 | 0.860 | 0.390 | 2.125 |
|  | Profession: |  |  |  |  |  |
|  | Executive – Independent | -0.808 | 0.457 | -1.767 | 0.077 | 0.446 |
|  | Technician – Independent | -0.850 | 0.451 | -1.886 | 0.059 | 0.427 |
|  | Employee – Independent | -0.487 | 0.442 | -1.103 | 0.270 | 0.615 |
|  | Worker – Independent | -0.643 | 0.559 | -1.151 | 0.250 | 0.526 |
|  | Other – Independent | 0.101 | 0.476 | 0.213 | 0.831 | 1.107 |

### MLR with VR

We conducted a multinomial logistic regression on the intention to get vaccinated against HPV with VR alone.

#### MLR unadjusted for sociodemographic variables

| **Model** | **Components** | **Deviance** | **BIC** | **R²_N_** | **Overall model test** | | |
| --- | --- | --- | --- | --- | --- | --- | --- |
|  |  |  |  |  | *χ²* | *dF* | *p-value* |
| 1 | Vaccine readiness | 1674 | 1704 | 0.4338 | 999.4 | 2 | < .001 |

Omnibus likelihood ratio tests were performed on each variable.

| **Predictor** | **χ²** | **dF** | **p** |
| --- | --- | --- | --- |
| Vaccine readiness score | 999.4 | 2 | < .001 |

|  | **Predictor** | **Estimate** | **SE** | **Z** | **p** | **OR** |
| --- | --- | --- | --- | --- | --- | --- |
| Refusal vs. Deliberating | Intercept | -1.112 | 0.172 | -6.461 | < .001 | 0.329 |
|  | Vaccine readiness score | -0.156 | 0.117 | -1.335 | 0.182 | 0.856 |
| Acceptance vs. Deliberating | Intercept | 1.996 | 0.103 | 19.466 | < .001 | 7.363 |
|  | Vaccine readiness score | 2.408 | 0.123 | 19.603 | < .001 | 11.112 |

#### MLR adjusted for sociodemographic variables

| **Model** | **Components** | **Deviance** | **BIC** | **R²_N_** | **Overall model test** | | |
| --- | --- | --- | --- | --- | --- | --- | --- |
|  |  |  |  |  | *χ²* | *dF* | *p-value* |
| 1 | Vaccine readiness | 1674 | 1704 | 0.4338 | 999.4 | 2 | < .001 |
| 2 | Gender  Age  Profession | 1643 | 1807 | 0.4461 | 1030.7 | 20 | < .001 |

| **Comparison** | | **Δχ²** | **Δdf** | **p-value** |
| --- | --- | --- | --- | --- |
| *Model* | *Model* |  |  |  |
| 1 | 2 | 31.29 | 18 | 0.027 |

The final model is statistically valid and explain 44% of the variance of the vaccination behaviour.

Omnibus likelihood ratio tests were performed on each variable.

| **Predictor** | **χ²** | **dF** | **p** |
| --- | --- | --- | --- |
| Vaccine readiness score | 989.395 | 2 | < .001 |
| Age | 4.013 | 2 | 0.134 |
| Gender | 3.632 | 6 | 0.726 |
| Profession | 22.126 | 10 | 0.014 |

|  | **Predictor** | **Estimate** | **SE** | **Z** | **p** | **OR** |
| --- | --- | --- | --- | --- | --- | --- |
| Refusal vs. Deliberating | Intercept | -1.146 | 0.714 | -1.604 | 0.109 | 0.318 |
|  | Vaccine readiness score | -0.087 | 0.123 | -0.711 | 0.477 | 0.917 |
|  | Age: |  |  |  |  |  |
|  | 35/44 – under 35 | 0.472 | 0.377 | 1.251 | 0.211 | 1.603 |
|  | 45/54 – under 35 |  |  |  |  |  |
|  | 55 and more – under 35 | 0.088 | 0.457 | 0.193 | 0.847 | 1.092 |
|  | Gender: | -0.028 | 0.473 | -0.059 | 0.953 | 0.973 |
|  | Male – Female | 0.245 | 0.980 | 0.250 | 0.803 | 1.277 |
|  | Profession: |  |  |  |  |  |
|  | Executive – Independent | -0.087 | 0.585 | -0.149 | 0.881 | 0.917 |
|  | Technician – Independent | -0.327 | 0.581 | -0.563 | 0.573 | 0.721 |
|  | Employee – Independent | 0.011 | 0.556 | 0.020 | 0.984 | 1.011 |
|  | Worker – Independent | -0.417 | 0.710 | -0.587 | 0.557 | 0.659 |
|  | Other – Independent | 0.655 | 0.575 | 1.140 | 0.254 | 1.925 |
| Acceptance vs. Deliberating | Intercept | 3.010 | 0.587 | 5.133 | < .001 | 20.294 |
|  | **Vaccine readiness score** | **2.526** | **0.130** | **19.468** | **< .001** | 12.500 |
|  | Age: |  |  |  |  |  |
|  | 35/44 – under 35 | 0.593 | 0.317 | 1.872 | 0.061 | 1.809 |
|  | 45/54 – under 35 |  |  |  |  |  |
|  | 55 and more – under 35 | -0.454 | 0.419 | -1.085 | 0.278 | 0.635 |
|  | Gender: | -0.481 | 0.431 | -1.117 | 0.264 | 0.618 |
|  | Male – Female | 0.561 | 0.857 | 0.655 | 0.513 | 1.753 |
|  | Profession: |  |  |  |  |  |
|  | Executive – Independent | -0.856 | 0.450 | -1.905 | 0.057 | 0.425 |
|  | Technician – Independent | -0.912 | 0.444 | -2.054 | 0.040 | 0.402 |
|  | Employee – Independent | -0.529 | 0.434 | -1.218 | 0.223 | 0.589 |
|  | Worker – Independent | -0.577 | 0.551 | -1.047 | 0.295 | 0.562 |
|  | Other – Independent | 0.080 | 0.468 | 0.171 | 0.865 | 1.083 |

## References

| Estabrook, R., & Neale, M. (2013). A Comparison of Factor Score Estimation Methods in the Presence of Missing Data: Reliability and an Application to Nicotine Dependence. *Multivariate Behavioral Research*, *48*(1), 1–27. https://doi.org/10.1080/00273171.2012.730072  Fox, J., & Weisberg, S. (2019). *An R companion to applied regression* (Third edition). SAGE.  fox, J., & Weisberg, S. (2021). *car: Companion to Applied Regression* (3.0-12) [R]. https://CRAN.R-project.org/package=car  Hooper, D., Coughlan, J., & Mullen, M. R. (2008). Structural Equation Modelling: Guidelines for Determining Model Fit. *Electronic Journal of Business Research Methods*, *6*(1), 53–60.  Hoyle, R. H. (Ed.). (2012). *Handbook of structural equation modeling*. Guilford Press.  Hu, L., & Bentler, P. M. (1999). Cutoff criteria for fit indexes in covariance structure analysis: Conventional criteria versus new alternatives. *Structural Equation Modeling: A Multidisciplinary Journal*, *6*(1), 1–55. https://doi.org/10.1080/10705519909540118  Lenth, R. V., Buerkner, P., Herve, M., Love, J., Miguez, F., Riebl, H., & Singmann, H. (2022). *emMeans: Estimated Marginal Means, aka Least-Squares Means* (1.7.4-1). https://CRAN.R-project.org/package=emmeans  Lüdecke, D., Ben-Shachar, M., Patil, I., Waggoner, P., & Makowski, D. (2021). performance: An R Package for Assessment, Comparison and Testing of Statistical Models. *Journal of Open Source Software*, *6*(60), 3139. https://doi.org/10.21105/joss.03139  Oudin Doglioni, D., Pham-Hung D’Alexandry D’Orengiani, A.-L., Galactéros, F., & Gay, M.-C. (2021). Psychometric characteristics of the Revised Illness Perception Questionnaire (IPQ-R) in adults with sickle cell disease. *Health Psychology and Behavioral Medicine*, *10*(1), 60–80. https://doi.org/10.1080/21642850.2021.2016411  R Core Team. (2021). *R: A Language and environment for statistical computing.* (4.1).  Revelle, W. (2022). *Psych: Procedures for Psychological, Psychometric, and Personality Research* (2.2.5) [R package]. Northwestern University. https://CRAN.R-project.org/package=psych  Ripley, B., & Venables, W. (2021). *nnet: Feed-Forward Neural Networks and Multinomial Log-Linear Models* (7.3-17) [R]. https://cran.r-project.org/web/packages/nnet/index.html  Rosseel, Y. (2012). **lavaan**: An *R* Package for Structural Equation Modeling. *Journal of Statistical Software*, *48*(2). https://doi.org/10.18637/jss.v048.i02  Rosseel, Y., & Jorgensen, T. D. (2019). *Package ‘lavaan’* (0.6-10) [R]. https://cran.r-project.org/web/packages/lavaan/lavaan.pdf  Steiger, J. H. (2002). When constraints interact: A caution about reference variables, identification constraints, and scale dependencies in structural equation modeling. *Psychological Methods*, *7*(2), 210–227. https://doi.org/10.1037/1082-989X.7.2.210  Steiger, J. H. (2007). Understanding the limitations of global fit assessment in structural equation modeling. *Personality and Individual Differences*, *42*(5), 893–898. https://doi.org/10.1016/j.paid.2006.09.017  The Jamovi Project. (2020). *Jamovi* (1.2).  Vuong, Q. H. (1989). Likelihood Ratio Tests for Model Selection and Non-Nested Hypotheses. *Econometrica*, *57*(2), 307. https://doi.org/10.2307/1912557  Wheaton, B., Muthen, B., Alwin, D. F., & Summers, G. F. (1977). Assessing Reliability and Stability in Panel Models. *Sociological Methodology*, *8*, 84–136. https://doi.org/10.2307/270754 |
| --- |

# Supplemental material 4: Adolescents – Human Papilloma Virus (HPV)

## First-order latent structure

**Objective**: to identify a latent factorial organisation referring to the antecedents of VR with a covariance relationship indicating the possible existence of a common formative second-order factor representing vaccine deliberating process or vaccine readiness (VR). As assessing the statistical significance of the loading for each indicators is important, we fixed the variance of the factor (Hoyle, 2012; Steiger, 2002) and freed all indicators. To estimate the factor score, we used the regression or exact method with the Bartlett’s correction for bias in factor means (Estabrook & Neale, 2013). The score produced have a mean of zero and a free standard deviation. It is understood that each antecedent should be defined by, at least, one item as the objective is not to validate a questionnaire but a theoretical model.

We used the two-index presentation strategy (Hu & Bentler, 1999) including: the maximum likelihood (ML)‐based standardised root mean squared residual (SRMR) with a cut-off value under .08; and, the root mean square of approximation (RMSEA) with 90% confidence intervals and cut-off value close to .07 (Steiger, 2007). We added 3 indicators of goodness of fit (Hooper et al., 2008): the Wheaton *et al.*’s relative/normed chi square with a range from 2 to 5 (Wheaton et al., 1977); the comparative fit index (CFI) with a value greater than .90; and, the non-normed fit index (NNFI) also known as the Tucker-Lewis index (TLI) with a .90 threshold (Oudin Doglioni et al., 2021).

| **Indices** | **Presentation** | **Cut off** | **Reference** |
| --- | --- | --- | --- |
| SRMR | Absolute fit indice which determine how well an *a priori* model fits the sample data. The SRMR are the square root of the difference between the residuals of the sample covariance matrix and the hypothesised covariance model. | <0.08 | Hu & Bentler, 1999 |
| RMSEA | The RMSEA tells us how well the model, with unknown but optimally chosen parameter estimates would fit the populations covariance matrix. the RMSEA favours parsimony in that it will choose the model with the lesser number of parameters. | <0.07 | Steiger, 2007 |
| relative/normed chi square | The Chi-Square value is the traditional measure for evaluating overall model fit and, assesses the magnitude of discrepancy between the sample and fitted covariances matrices. Because the Chi-Square statistic is in essence a statistical significance test it is sensitive to sample size which means that the Chi-Square statistic nearly always rejects the model when large samples are used. One example of a statistic that minimises the impact of sample size on the model Chi-Square is Wheaton *et al*’s relative/normed chi-square (χ2/df). | [2;5] | Wheaton et al., 1977 |
| CFI | The Comparative Fit Index is a revised form of the NFI which takes into account sample size that performs well even when sample size is small. | >0.90 | Hu & Bentler, 1999 |
| NNFI/TLI | the Non-Normed Fit Index assesses the model by comparing the χ2 value of the model to the χ2 of the null model. | >0.90 | Hooper et al.; 2008 |

The statistics were carried out using Jamovi software (The Jamovi Project, 2020), version 2.3 or R Studio, version 3.6, both run under R (R Core Team, 2021), version 4.1, and the Laavan (Rosseel, 2012; Rosseel & Jorgensen, 2019), the Psych (Revelle, 2022), the Car libraries (Fox & Weisberg, 2019; fox & Weisberg, 2021) and the Emmeans library (Lenth et al., 2022).

Script used in Jamovi and R Studio are indicative. In particular, word written in italic refers to generic terms that must be adapted to the specific context.

*Script:*

| Model = ‘*factor* =~ *variable of interest*’  Model.fit = cfa(Model, data = *data*, missing = “ML”)  summary(Model.fit, fit.measures = TRUE)  Model.fit.predict = lavpredict(Model.fit, type = “lv”, method = “Bartlett”, label = TRUE, std.lv = TRUE) |
| --- |
| jmv::cfa(data = data,  factors = list(  list(label="*factor name*", vars=c("*variables of interest*")),  resCov = NULL,  stdEst = TRUE,  fitMeasures = c("cfi", "tli", "rmsea", "srmr", "aic")  mi = TRUE) |

### Exploration of each antecedents

#### Convenience

All indicators are significantly participate to the definition of the latent factor. But some loaded poorly to the factor (in bold).

| **Factor** | **Indicator** | **Estimate** | **SE** | **Z** | **p** | **Stand. Estimate** |
| --- | --- | --- | --- | --- | --- | --- |
| Convenience | **Zc2_k1_T** | **0.172** | **0.018** | **9.663** | **< .001** | **0.172** |
|  | **Zc2_k2_T** | **0.232** | **0.018** | **12.973** | **< .001** | **0.232** |
|  | Zc2_a1_T | -0.348 | 0.018 | -19.637 | < .001 | -0.348 |
|  | Zc2_a2 | -0.703 | 0.019 | -36.470 | < .001 | -0.703 |
|  | Zc2_a3 | -0.681 | 0.019 | -35.991 | < .001 | -0.682 |

As a consequence, goodness of fit indices are not indicating that the latent factor is well defined.

| **Antecedent** | **CFI** | **TLI** | **SRMR** | **RMSEA** | | | **Chi²/dF** | **AIC** |
| --- | --- | --- | --- | --- | --- | --- | --- | --- |
|  |  |  |  |  | 90% CI | |  |  |
|  | *>0.90* | *>0.90* | *<0.08* | *<0.07* | *Lower* | *Upper* | *<5* |  |
| Convenience | 0.8519 | 0.7038 | 0.05529 | 0.1172 | 0.1068 | 0.1281 | 66.86 | 65977 |

#### Complacency

All indicators are significantly participate to the definition of the latent factor, and loading are higher than 0.30.

| **Factor** | **Indicator** | **Estimate** | **SE** | **Z** | **p** | **Stand. Estimate** |
| --- | --- | --- | --- | --- | --- | --- |
| Complacency | Zc3_k1_T | 0.351 | 0.018 | 19.610 | < .001 | 0.351 |
|  | Zc3_k2_T | 0.371 | 0.018 | 20.790 | < .001 | 0.371 |
|  | Zc3_a1_T | -0.638 | 0.019 | -33.130 | < .001 | -0.638 |
|  | Zc3_a2 | -0.674 | 0.020 | -34.120 | < .001 | -0.675 |

Goodness of fit indices above their thresholds.

| **Antecedent** | **CFI** | **TLI** | **SRMR** | **RMSEA** | | | **Chi²/dF** | **AIC** |
| --- | --- | --- | --- | --- | --- | --- | --- | --- |
|  |  |  |  |  | 90% CI | |  |  |
|  | *>0.90* | *>0.90* | *<0.08* | *<0.07* | *Lower* | *Upper* | *<5* |  |
| Complacency | 0.9787 | 0.9361 | 0.02058 | 0.06337 | 0.04725 | 0.08108 | 20.24 | 52555 |

### Exploration of the theoretical model

#### Definition of the seven latent factors structure

##### Initial input (model 1)

All indicators are significantly participate to the definition of their respective latent factor. Indicators with loading (standard estimate) higher than 0.30 are of enough contribution. Indicators with loading under .30 should be removed in a Model 2 (standard estimate in bold).

| **Factor** | **Indicator** | **Estimate** | **SE** | **Z** | **p** | **Stand. Estimate** |
| --- | --- | --- | --- | --- | --- | --- |
| Confidence system | Zconfiance_systeme | 1.000 | 0.008 | 119.315 | < .001 | 1.000 |
| Confidence vaccine | **Zc1_k1_T** | **0.011** | **0.038** | **0.282** | **0.778** | **0.011** |
|  | **Zc1_a1** | **-0.192** | **0.634** | **-0.303** | **0.762** | **-0.192** |
| Convenience | Zc2_k1_T | 0.305 | 0.016 | 19.365 | < .001 | 0.305 |
|  | Zc2_k2_T | 0.372 | 0.016 | 23.763 | < .001 | 0.372 |
|  | Zc2_a1_T | -0.727 | 0.015 | -48.651 | < .001 | -0.727 |
|  | Zc2_a2 | -0.317 | 0.016 | -19.304 | < .001 | -0.317 |
|  | Zc2_a3 | -0.326 | 0.016 | -20.042 | < .001 | -0.326 |
| Complacency | Zc3_k1_T | 0.322 | 0.015 | 21.308 | < .001 | 0.322 |
|  | Zc3_k2_T | 0.341 | 0.015 | 22.688 | < .001 | 0.341 |
|  | Zc3_a1_T | -0.739 | 0.014 | -53.222 | < .001 | -0.739 |
|  | Zc3_a2 | -0.597 | 0.014 | -41.418 | < .001 | -0.597 |
| Calculation | **Zc4_k1_T** | **0.189** | **0.019** | **9.886** | **< .001** | **0.189** |
|  | Zc4_a1 | 0.481 | 0.035 | 13.801 | < .001 | 0.481 |
| Coll. responsibility | **Zc5_k1_T** | **0.187** | **0.018** | **10.210** | **< .001** | **0.187** |
|  | Zc5_a1 | 0.524 | 0.035 | 15.081 | < .001 | 0.523 |
| Social conformism | Zc6_a1 | 1.001 | 0.010 | 97.524 | < .001 | 1.000 |

As all factor should be defined, for confidence vaccine, the higher loading indicator (Zc1_a1) should be kept. All factors do not covary. In particular, Confidence in vaccine that is mispecified covary with no other latent factors.

| **Factor** | | **Estimate** |  | **SE** | **Z** | **p** | **Stand. Estimate** |
| --- | --- | --- | --- | --- | --- | --- | --- |
| Confidence system | Confidence system | 1.000 | ᵃ |  |  |  |  |
|  | **Confidence vaccine** | **-0.616** |  | **2.030** | **-0.303** | **0.762** | **-0.616** |
|  | Complacency | 0.099 |  | 0.019 | 5.355 | < .001 | 0.099 |
|  | Convenience | 0.108 |  | 0.018 | 6.047 | < .001 | 0.108 |
|  | Calculation | 0.177 |  | 0.031 | 5.716 | < .001 | 0.177 |
|  | Coll. responsibility | 0.198 |  | 0.029 | 6.777 | < .001 | 0.198 |
|  | Social conformism | 0.134 |  | 0.014 | 9.314 | < .001 | 0.134 |
| Confidence vaccine | Confidence vaccine | 1.000 | ᵃ |  |  |  |  |
|  | **Complacency** | **-2.998** |  | **9.881** | **-0.303** | **0.762** | **-2.998** |
|  | **Convenience** | **-3.141** |  | **10.352** | **-0.303** | **0.762** | **-3.141** |
|  | **Calculation** | **-4.266** |  | **14.064** | **-0.303** | **0.762** | **-4.266** |
|  | **Coll. responsibility** | **-3.469** |  | **11.437** | **-0.303** | **0.762** | **-3.469** |
|  | **Social conformism** | **-1.505** |  | **4.960** | **-0.303** | **0.762** | **-1.505** |
| Complacency | Complacency | 1.000 | ᵃ |  |  |  |  |
|  | Convenience | 1.007 |  | 0.015 | 67.709 | < .001 | 1.007 |
|  | Calculation | 1.071 |  | 0.076 | 14.096 | < .001 | 1.071 |
|  | Coll. responsibility | 0.912 |  | 0.060 | 15.260 | < .001 | 0.912 |
|  | Social conformism | 0.384 |  | 0.017 | 23.039 | < .001 | 0.384 |
| Convenience | Convenience | 1.000 | ᵃ |  |  |  |  |
|  | Calculation | 1.188 |  | 0.081 | 14.659 | < .001 | 1.188 |
|  | Coll. responsibility | 1.202 |  | 0.074 | 16.308 | < .001 | 1.202 |
|  | Social conformism | 0.499 |  | 0.014 | 34.542 | < .001 | 0.499 |
| Calculation | Calculation | 1.000 | ᵃ |  |  |  |  |
|  | Coll. responsibility | 1.417 |  | 0.132 | 10.752 | < .001 | 1.417 |
|  | Social conformism | 0.681 |  | 0.050 | 13.651 | < .001 | 0.681 |
| Coll. responsibility | Coll. responsibility | 1.000 | ᵃ |  |  |  |  |
|  | Social conformism | 0.573 |  | 0.042 | 13.751 | < .001 | 0.573 |
| ᵃ fixed parameter |  | 1.000 | ᵃ |  |  |  |  |

Goodness of fit indices do not reach the thresholds to indicate a good fit of the theoretical model in our population.

|  | **CFI** | **TLI** | **SRMR** | **RMSEA** | | | **Chi²/dF** | **AIC** |
| --- | --- | --- | --- | --- | --- | --- | --- | --- |
|  |  |  |  |  | 90% CI | |  |  |
|  | *>0.90* | *>0.90* | *<0.08* | *<0.07* | *Lower* | *Upper* | *<5* |  |
| Initial model | 0.8264 | 0.7640 | 0.04958 | 0.05874 | 0.05678 | 0.06072 | 25.56 | 225430 |

To improve the fit, low loading indicators were removed in a second model.

##### Removal of non-contributory indicators (model 2)

All indicators contributes to the definition of their respective latent factors and loading are all above 0.30. For Confidence vaccine, we kept the higher loaded indicator.

| **Factor** | | **Indicator** | | **Estimate** | | **SE** | | **Z** | **p** | **Stand. Estimate** |
| --- | --- | --- | --- | --- | --- | --- | --- | --- | --- | --- |
| Confidence system | Zconfiance_systeme | | 1.000 | | 0.008 | | 119.310 | | < .001 | 1.000 |
| Confidence vaccine | Zc1_a1 | | 1.000 | | 0.010 | | 97.560 | | < .001 | 1.000 |
| Convenience | Zc2_k1_T | | 0.304 | | 0.016 | | 19.230 | | < .001 | 0.304 |
|  | Zc2_k2_T | | 0.371 | | 0.016 | | 23.680 | | < .001 | 0.371 |
|  | Zc2_a1_T | | -0.728 | | 0.015 | | -48.560 | | < .001 | -0.728 |
|  | Zc2_a2 | | -0.318 | | 0.016 | | -19.290 | | < .001 | -0.318 |
|  | Zc2_a3 | | -0.328 | | 0.016 | | -20.080 | | < .001 | -0.328 |
| Complacency | Zc3_k1_T | | 0.320 | | 0.015 | | 21.110 | | < .001 | 0.320 |
|  | Zc3_k2_T | | 0.338 | | 0.015 | | 22.440 | | < .001 | 0.338 |
|  | Zc3_a1_T | | -0.741 | | 0.014 | | -53.340 | | < .001 | -0.741 |
|  | Zc3_a2 | | -0.598 | | 0.014 | | -41.560 | | < .001 | -0.598 |
| Calculation | Zc4_a1 | | 1.000 | | 0.010 | | 97.510 | | < .001 | 1.000 |
| Coll. responsibility | Zc5_a1 | | 1.000 | | 0.010 | | 97.540 | | < .001 | 1.000 |
| Social conformism | Zc6_a1 | | 1.001 | | 0.010 | | 97.510 | | < .001 | 1.000 |

All factors covary suggesting that they refer to a common latent second-order.

| **Factor** | | **Estimate** |  | **SE** | **Z** | **p** | **Stand. Estimate** |
| --- | --- | --- | --- | --- | --- | --- | --- |
| Confidence system | Confidence system | 1.000 | ᵃ |  |  |  |  |
|  | Confidence vaccine | 0.119 |  | 0.014 | 8.267 | < .001 | 0.119 |
|  | Complacency | 0.099 |  | 0.018 | 5.362 | < .001 | 0.099 |
|  | Convenience | 0.108 |  | 0.018 | 6.067 | < .001 | 0.108 |
|  | Calculation | 0.084 |  | 0.015 | 5.750 | < .001 | 0.084 |
|  | Coll. responsibility | 0.099 |  | 0.015 | 6.830 | < .001 | 0.099 |
|  | Social conformism | 0.134 |  | 0.014 | 9.320 | < .001 | 0.134 |
| Confidence vaccine | Confidence vaccine | 1.000 | ᵃ |  |  |  |  |
|  | Complacency | 0.574 |  | 0.014 | 40.162 | < .001 | 0.574 |
|  | Convenience | 0.604 |  | 0.013 | 46.861 | < .001 | 0.604 |
|  | Calculation | 0.395 |  | 0.012 | 32.295 | < .001 | 0.395 |
|  | Coll. responsibility | 0.341 |  | 0.013 | 26.552 | < .001 | 0.341 |
|  | Social conformism | 0.290 |  | 0.013 | 21.806 | < .001 | 0.290 |
| Complacency | Complacency | 1.000 | ᵃ |  |  |  |  |
|  | Convenience | 1.006 |  | 0.015 | 67.589 | < .001 | 1.006 |
|  | Calculation | 0.494 |  | 0.015 | 32.143 | < .001 | 0.494 |
|  | Coll. responsibility | 0.485 |  | 0.016 | 31.103 | < .001 | 0.485 |
|  | Social conformism | 0.384 |  | 0.017 | 23.054 | < .001 | 0.384 |
| Convenience | Convenience | 1.000 | ᵃ |  |  |  |  |
|  | Calculation | 0.569 |  | 0.013 | 42.337 | < .001 | 0.569 |
|  | Coll. responsibility | 0.638 |  | 0.012 | 51.427 | < .001 | 0.638 |
|  | Social conformism | 0.499 |  | 0.014 | 34.581 | < .001 | 0.499 |
| Calculation | Calculation | 1.000 | ᵃ |  |  |  |  |
|  | Coll. responsibility | 0.367 |  | 0.013 | 29.225 | < .001 | 0.367 |
|  | Social conformism | 0.332 |  | 0.013 | 25.724 | < .001 | 0.332 |
| Coll. responsibility | Coll. responsibility | 1.000 | ᵃ |  |  |  |  |
|  | Social conformism | 0.287 |  | 0.013 | 21.590 | < .001 | 0.287 |
| ᵃ fixed parameter |  |  |  |  |  |  |  |

Indices do not reach the threshold to indicate a good fit of the theoretical model in our population.

|  | **CFI** | **TLI** | **SRMR** | **RMSEA** | | | **Chi²/dF** | **AIC** |
| --- | --- | --- | --- | --- | --- | --- | --- | --- |
|  |  |  |  |  | 90% CI | |  |  |
|  | *>0.90* | *>0.90* | *<0.08* | *<0.07* | *Lower* | *Upper* | *<5* |  |
| Model 2 | **0.8342** | **0.7527** | 0.05568 | 0.07193 | 0.06943 | **0.07446** | 37.82 | 185437 |

Modification indices suggest adding covariance between indicators from the same latent factors which was done in a Model 3:

- **Zc2_a3** (‘I find it difficult to discuss HPV vaccination with a health professional (doctor, nurse, etc.).’) | **Zc2_a2** (‘I find it difficult to talk about HPV vaccination with my relative.’) which refer to the difficulties to speak about HPV vaccination (MI = 953.6255);
- **Zc2_k2_T** (‘HPV vaccination results in a better immune response [better antibody production] if given before age 14’) | **Zc2_k2_T** (‘HPV vaccination is ideally administered’ with suggested responses using age ranges) refer to the ideal age or age range for completing HPV vaccination (MI = 219.1967);
- **Zc3_a2** (‘I find myself too young to be vaccinated against HPV.’) | **Zc2_a2** (‘I find it difficult to talk about HPV with my relatives.’) which refer to the age-related difficulties of talking about sexual issues (MI = 141.217);
- **Zc3_a2** (‘I find myself too young to be vaccinated against HPV.’) | **Zc2_a3** (‘I find it difficult to talk about HPV vaccination with HCP.’) which refer to the age-related difficulties of talking about sexual issues (MI = 132.308).

These additions were made in a third model.

##### Addition of covariance between indicators (model 3)

*Script:*

| Model = ‘*factor* =~ *variable of interest*  *indicator~~indicator*’  Model.fit = cfa(Model, data = *data*, missing = “ML”)  summary(Model.fit, fit.measures = TRUE)  modindices(Model.fit, sort = TRUE, maximum.number = 5)  Model.fit.predict = lavpredict(Model.fit, type = “lv”, method = “Bartlett”, label = TRUE, std.lv = TRUE) |
| --- |
| jmv::cfa(data = data,  factors = list(  list(label="*factor name*", vars=c("*variables of interest*")),  resCov = list (  list (i_n_ = "*variables of interest*")),  stdEst = TRUE,  fitMeasures = c("cfi", "tli", "rmsea", "srmr", "aic")  mi = TRUE) |

The indicators: Zc2_k1_T, Zc2_a2 and Zc2_a3 became non-contributing when the covariance were added. These indicators were removed. As a consequence, only the covariance between Zc2_k2_T and Zc3_k2_T remain. All indicators significantly contribute to the definition of their respective latent factor

| **Factor** | **Indicator** | | **Estimate** | | **SE** | | **Z** | | **p** | **Stand. Estimate** |
| --- | --- | --- | --- | --- | --- | --- | --- | --- | --- | --- |
| Confidence system | Zconfiance_systeme | 1.000 | | 0.008 | | 119.310 | | < .001 | | 1.000 |
| Confidence vaccine | Zc1_a1 | 1.000 | | 0.010 | | 97.590 | | < .001 | | 1.000 |
| Convenience | Zc2_k2_T | 0.331 | | 0.016 | | 20.660 | | < .001 | | 0.331 |
|  | Zc2_a1_T | -0.741 | | 0.021 | | -35.470 | | < .001 | | -0.741 |
| Complacency | Zc3_k1_T | 0.301 | | 0.015 | | 19.620 | | < .001 | | 0.301 |
|  | Zc3_k2_T | 0.314 | | 0.015 | | 20.560 | | < .001 | | 0.315 |
|  | Zc3_a1_T | -0.777 | | 0.014 | | -56.370 | | < .001 | | -0.776 |
|  | Zc3_a2 | -0.575 | | 0.014 | | -39.710 | | < .001 | | -0.575 |
| Calculation | Zc4_a1 | 1.000 | | 0.010 | | 97.530 | | < .001 | | 1.000 |
| Coll. responsibility | Zc5_a1 | 1.000 | | 0.010 | | 97.560 | | < .001 | | 1.000 |
| Social conformism | Zc6_a1 | 1.001 | | 0.010 | | 97.530 | | < .001 | | 1.000 |

All factors covary suggesting the existence of a second-order latent factor.

| **Factor** | | **Estimate** |  | **SE** | **Z** | **p** | **Stand. Estimate** |
| --- | --- | --- | --- | --- | --- | --- | --- |
| Confidence system | Confidence system | 1.000 | ᵃ |  |  |  |  |
|  | Confidence vaccine | 0.119 |  | 0.014 | 8.242 | < .001 | 0.119 |
|  | Complacency | 0.116 |  | 0.019 | 6.019 | < .001 | 0.116 |
|  | Convenience | 0.115 |  | 0.017 | 6.568 | < .001 | 0.115 |
|  | Calculation | 0.083 |  | 0.015 | 5.731 | < .001 | 0.083 |
|  | Coll. responsibility | 0.099 |  | 0.015 | 6.810 | < .001 | 0.099 |
|  | Social conformism | 0.134 |  | 0.014 | 9.306 | < .001 | 0.134 |
| Confidence vaccine | Confidence vaccine | 1.000 | ᵃ |  |  |  |  |
|  | Complacency | 0.616 |  | 0.018 | 33.710 | < .001 | 0.616 |
|  | Convenience | 0.602 |  | 0.013 | 47.524 | < .001 | 0.602 |
|  | Calculation | 0.395 |  | 0.012 | 32.281 | < .001 | 0.395 |
|  | Coll. responsibility | 0.340 |  | 0.013 | 26.540 | < .001 | 0.340 |
|  | Social conformism | 0.290 |  | 0.013 | 21.797 | < .001 | 0.290 |
| Complacency | Complacency | 1.000 | ᵃ |  |  |  |  |
|  | Convenience | 0.976 |  | 0.024 | 40.677 | < .001 | 0.976 |
|  | Calculation | 0.518 |  | 0.019 | 27.829 | < .001 | 0.518 |
|  | Coll. responsibility | 0.474 |  | 0.019 | 25.180 | < .001 | 0.474 |
|  | Social conformism | 0.441 |  | 0.019 | 23.808 | < .001 | 0.441 |
| Convenience | Convenience | 1.000 | ᵃ |  |  |  |  |
|  | Calculation | 0.565 |  | 0.013 | 42.654 | < .001 | 0.565 |
|  | Coll. responsibility | 0.634 |  | 0.012 | 52.119 | < .001 | 0.634 |
|  | Social conformism | 0.500 |  | 0.014 | 35.498 | < .001 | 0.500 |
| Calculation | Calculation | 1.000 | ᵃ |  |  |  |  |
|  | Coll. responsibility | 0.367 |  | 0.013 | 29.210 | < .001 | 0.367 |
|  | Social conformism | 0.332 |  | 0.013 | 25.714 | < .001 | 0.332 |
| Coll. responsibility | Coll. responsibility | 1.000 | ᵃ |  |  |  |  |
|  | Social conformism | 0.287 |  | 0.013 | 21.582 | < .001 | 0.287 |
| ᵃ fixed parameter |  |  |  |  |  |  |  |

Goodness of fit indices do reach the thresholds to indicate a good fit of the theoretical model in our population.

|  | **CFI** | **TLI** | **SRMR** | **RMSEA** | | | **Chi²/dF** | **AIC** |
| --- | --- | --- | --- | --- | --- | --- | --- | --- |
|  |  |  |  |  | 90% CI | |  |  |
|  | *>0.90* | *>0.90* | *<0.08* | *<0.07* | *Lower* | *Upper* | *<5* |  |
| Model 3 | 0.9577 | 0.9139 | 0.03144 | 0.04908 | 0.04533 | 0.05294 | 18.14 | 145204 |

Although Wheaton *et al*’s relative/normed chi-square (Chi2/df) minimises the impact of sample size on the model, in the overall adolescents database, sample size is too large (n > 5000). In adolescents from the general population, a latent organisation on seven first-order factors is demonstrated.

##### CFA synthesis

Definition of the seven latent factors structure show constant improvement of the fit to the data.

|  | **General population – Adolescents (PrevHPV database)** | | | | | | | |
| --- | --- | --- | --- | --- | --- | --- | --- | --- |
| **Model** | **CFI** | **TLI** | **SRMR** | **RMSEA** | | | **Chi²/dF** | **AIC** |
|  |  |  |  |  | 90% CI | |  |  |
|  | *>0.90* | *>0.90* | *<0.08* | *<0.07* | *Lower* | *Upper* | *<5* |  |
| Initial | 0.8264 | 0.7640 | 0.04958 | 0.05874 | 0.05678 | 0.06072 | 25.56 | 225430 |
| #2 | 0.8342 | 0.7527 | 0.05568 | 0.07193 | 0.06943 | 0.07446 | 37.82 | 185437 |
| final | 0.9577 | 0.9139 | 0.03144 | 0.04908 | 0.04533 | 0.05294 | 18.14 | 145204 |

#### First-order latent structure internal consistency

*Script:*

| jmv::reliability(data = data,  vars = vars(*variables of interest*),  alphaScale = FALSE,  omegaScale = TRUE) |
| --- |

| **Internal consistency – 1^st^-order** | **McDonald’s ώ** |
| --- | --- |
| General population – Adolescents | 0.7905 |

Reliability analysis demonstrate a good internal consistency of the first-order latent structure.

### Latent factors scores estimation

In order to respect the meaning of the VR concept, no reverse coding must be apply:

| Hight VR when | High | Confidence system |  | Confidence system |
| --- | --- | --- | --- | --- |
|  | High | Confidence vaccine |  | Confidence vaccine |
|  | Low | Complacency |  | Low complacency |
|  | High | Convenience |  | High convenience |
|  | High | Calculation |  | High benefice risk balance (BRB) |
|  | High | Collective responsibility |  | Collective responsibility |
|  | Low | Social conformism |  | Favourable environment |

Mean score per subpopulation:

| **Adolescents** | **Confidence system** | **Confidence vaccine** | **High convenience** | **Low complacency** | **High BRB** | **Collective responsibility** | **Favourable environment** |
| --- | --- | --- | --- | --- | --- | --- | --- |
| Overall | -1.83e−5 | 0.004 | -6.08e−4 | -0.005 | 0.003 | 0.003 | 0.004 |
| 4^th^ grade | 0.069 | -0.028 | -0.047 | -0.068 | -0.047 | -0.047 | -0.005 |
| 3^rd^ grade | -0.080 | 0.038 | 0.049 | 0.063 | 0.057 | 0.058 | 0.014 |

Kiviat diagram show opposition between the two groups.

Per subpopulations mean comparison show significant differences for all the factors except for Sceptic environment.

*Script:*

| jmv::ttestIS(formula = ADO_distrust_system + ADO_distrust_vaccine + ADO_low_convenience + ADO_high_compalcency + ADO_low_BRB + ADO_selfcentred_vision + ADO_sceptic_environment ~ classe,  data = data,  vars = vars(ADO_distrust_system, ADO_distrust_vaccine, ADO_low_convenience, ADO_high_compalcency, ADO_low_BRB, ADO_selfcentred_vision, ADO_sceptic_environment),  welchs = TRUE,  norm = TRUE,  eqv = TRUE,  effectSize = TRUE) |
| --- |

| **Adolescents** | **Test** | **Statistic** | **Df** | **p-value** | **Cohen’s d** |
| --- | --- | --- | --- | --- | --- |
| Confidence system | Student's t | 6.2875 | 7116 | < .001 | -0.149 |
| Confidence vaccine | Welch's t | -2.2907 | 4779 | 0.022 | 0.066 |
| Low complacency | Welch's t | -2.4946 | 4779 | 0.013 | -0.072 |
| High convenience | Welch's t | -3.7291 | 4786 | < .001 | -0.108 |
| High BRB | Student's t | -3.5836 | 4765 | < .001 | 0.104 |
| Collective responsibility | Student's t | -3.6008 | 4762 | < .001 | 0.104 |
| Favourable environment | Student's t | -0.6585 | 4764 | 0.510 | 0.019 |

## Second-order latent structure

*Script:*

| Model = ‘*factor* =~ *variable of interest*’  Model.fit = cfa(Model, data = *data*, missing = “ML”)  summary(Model.fit, fit.measures = TRUE)  Model.fit.predict = lavpredict(Model.fit, type = “lv”, method = “Bartlett”, label = TRUE, std.lv = TRUE) |
| --- |
| jmv::cfa(data = data,  factors = list(  list(label="*factor name*", vars=c("*variables of interest*")),  resCov = NULL,  stdEst = TRUE,  fitMeasures = c("cfi", "tli", "rmsea", "srmr", "aic")  mi = TRUE) |

### Definition of the latent factor

#### Confirmatory factor analysis

All indicators significantly contribute to the definition of the latent factor but Confidence in the system show a loading under 0.30 indicating that it participates poorly to the definition of the second order latent factor.

| **Factor** | **Indicator** | **Estimate** | **SE** | **Z** | **p** | **Stand. Estimate** |
| --- | --- | --- | --- | --- | --- | --- |
| VR | Confidence system | 0.145 | 0.016 | 9.244 | < .001 | **0.145** |
|  | Confidence vaccine | 0.607 | 0.014 | 42.601 | < .001 | 0.608 |
|  | High convenience | 0.935 | 0.018 | 51.225 | < .001 | 0.700 |
|  | Low complacency | 1.030 | 0.016 | 65.407 | < .001 | 0.846 |
|  | High BRB | 0.583 | 0.014 | 40.228 | < .001 | 0.582 |
|  | Collective responsibility | 0.594 | 0.014 | 41.348 | < .001 | 0.593 |
|  | Social conformism | 0.497 | 0.015 | 33.470 | < .001 | 0.496 |

The goodness of fit indices do reach the thresholds to indicate a good fit of the theoretical model in our population.

|  | **CFI** | **TLI** | **SRMR** | **RMSEA** | | | **Chi²/dF** | **AIC** |
| --- | --- | --- | --- | --- | --- | --- | --- | --- |
|  |  |  |  |  | 90% CI | |  |  |
|  | *>0.90* | *>0.90* | *<0.08* | *<0.07* | *Lower* | *Upper* | *<5* |  |
| Second order | 0.974 | 0.961 | 0.023 | 0.046 | 0.041 | 0.051 | 16.064 | 98269 |

Although Wheaton *et al*’s relative/normed chi-square (Chi2/dF) minimises the impact of sample size on the model, in the PrevHPV database sample size is too large (n> 7000). In adolescents from the general population, a second order latent variable is demonstrated.

#### Second-order latent structure internal consistency

| **Internal consistency – 2^nd^-order** | **McDonald’s ώ** |
| --- | --- |
| General population – Adolescents | 0.7711 |

Reliability analysis demonstrate a good internal consistency of the second-order latent structure.

#### Second-order latent score estimation

Mean score per subpopulation:

| **Adults** | **Vaccine readiness** |
| --- | --- |
| Overall | 0.011 |
| 4^th^ grade | -0.049 |
| 3^rd^ grade | 0.076 |

Per group mean comparison show significant differences with higher VR in 3^rd^ grade than in 4^th^:

| **Adolescents** | **Test** | **Statistic** | **p-value** |
| --- | --- | --- | --- |
| Vaccine readiness | Mann-Whitney U | 2.661e+6 | <.001 |

### Comparison with a 5 C theoretical model

#### Definition of a non-nested 5 C model

We followed the same procedure as for the seven latent factors antecedents to obtain a fitted model with five latent factors antecedents. This 5 C concurrent model (CM) is not nested to the 7 C. The aim is to propose an unconstrained 5 C model to give it every chance of outperforming our proposed 7 C model.

*Script:*

| ADO_5C='  ADO_confidence_vaccine =~ Zc1_k1_T + Zc1_a1  ADO_convenience =~ Zc2_k1_T + Zc2_k2_T + Zc2_a1_T + Zc2_a2 + Zc2_a3  ADO_compalcency =~ Zc3_k1_T + Zc3_k2_T + Zc3_a1_T + Zc3_a2  ADO_calculation =~ Zc4_k1_T + Zc4_a1  ADO_coll_responsibility =~ Zc5_k1_T + Zc5_a1  '  ADO_5C.2='  ADO_confidence_vaccine =~ Zc1_a1  ADO_convenience =~ Zc2_k1_T + Zc2_k2_T + Zc2_a1_T + Zc2_a2 + Zc2_a3  ADO_compalcency =~ Zc3_k1_T + Zc3_k2_T + Zc3_a1_T + Zc3_a2  ADO_calculation =~ Zc4_a1  ADO_coll_responsibility =~ Zc5_a1  '  ADO_5C.3='  ADO_confidence_vaccine =~ Zc1_a1  ADO_convenience =~ Zc2_k1_T + Zc2_k2_T + Zc2_a1_T  ADO_compalcency =~ Zc3_k1_T + Zc3_k2_T + Zc3_a1_T + Zc3_a2  ADO_calculation =~ Zc4_a1  ADO_coll_responsibility =~ Zc5_a1  Zc2_k2_T ~~Zc3_k2_T' |
| --- |

Goodness of fit indices do reach the thresholds to indicate a good fit of the theoretical model in our population. Although Wheaton *et al*’s relative/normed chi-square (Chi2/df) minimises the impact of sample size on the model, in the overall adolescents database, sample size is too large (n > 5000).

| **Adults** | **CFI** | **TLI** | **SRMR** | **RMSEA** | | | **Chi²/dF** | **AIC** |
| --- | --- | --- | --- | --- | --- | --- | --- | --- |
|  |  |  |  |  | 90% CI | |  |  |
|  | *>0.90* | *>0.90* | *<0.08* | *<0.07* | *Lower* | *Upper* | *<5* |  |
| 5 C model | 0.950 | 0.916 | 0.036 | 0.063 | 0.058 | 0.067 | 19.83 | 125921 |

A five latent factors structure is defined in our population.

#### Vuong’s test for model selection

We performed a Vuong’s test (Vuong, 1989) with package Performance (Lüdecke et al., 2021) run with R Studio, version 3.6, run under R (R Core Team, 2021), version 4.1.

*Script:*

| fit1.ado = lm(Mean_VR ~ CM_distrust_vaccine+  CM_low_convenience+  CM_high_complacency+  CM_low_BRB+  CM_selfcentred_vision, data = ADO_v2)  fit2.ado = lm(Mean_VR ~  ADO_distrust_vaccine+  ADO_low_convenience+  ADO_high_compliance+  ADO_low_BRB+  ADO_selfcentred_vision+  ADO_sceptic_environment, data = ADO_v2)  fit3.ado = lm(Mean_VR ~ ADO_distrust_system+  ADO_distrust_vaccine+  ADO_low_convenience+  ADO_high_compliance+  ADO_low_BRB+  ADO_selfcentred_vision+  ADO_sceptic_environment, data = ADO_v2)  test_vuong(fit1.ado, fit2.ado, fit3.ado)  test_wald(fit2.ado, fit3.ado) |
| --- |

The Vuong’s test consist of two tests:

1. The Test of Distinguishability (the Omega2 [*Ώ²*] column and its associated p-value) indicates whether or not the models can possibly be distinguished on the basis of the observed data. If its p-value is significant, it means the models are distinguishable;
2. The Robust Likelihood Test (the LR column and its associated p-value) indicates whether each model fits better than the reference model, set as the 5 C model.

| **Model** | **Distinguishability** | | **Robust Likelihood Test** | |
| --- | --- | --- | --- | --- |
|  | *Ώ²* | *p-value* | *LR value* | *p-value* |
| 5 C |  |  |  |  |
| 7 C | 1.75 | <0.001 | -63.61 | <0.001 |

The models are distinguishable and the 7 C model fit better than the 5C model.

### Comparison within the 7C model

**Objective**: based on the 7C model, to demonstrate that addition of antecedents of vaccine readiness improve the definition of the dependent variable.

*Script:*

| fit7C.ADO=lm(ADO_VR ~  ADO_distrust_system +  ADO_distrust_vaccine +  ADO_low_convenience +  ADO_high_compliance +  ADO_low_BRB +  ADO_selfcentred_vision +  ADO_sceptic_environment, data = ADO_v2)  fit6C.ADO=lm(ADO_VR ~  ADO_distrust_system +  ADO_distrust_vaccine +  ADO_low_convenience +  ADO_high_compliance +  ADO_low_BRB +  ADO_selfcentred_vision, data = ADO_v2)  fit6.2C.ADO=lm(ADO_VR ~  ADO_distrust_vaccine +  ADO_low_convenience +  ADO_high_compliance +  ADO_low_BRB +  ADO_selfcentred_vision +  ADO_sceptic_environment, data = ADO_v2)  fit5C.ADO=lm(ADO_VR ~  ADO_distrust_vaccine +  ADO_low_convenience +  ADO_high_compliance +  ADO_low_BRB +  ADO_selfcentred_vision, data = ADO_v2)  test_wald(fit5C.ADO, fit6C.ADO, fit7C.ADO)  test_wald(fit5C.ADO, fit6.2C.ADO, fit7C.ADO)  compare_performance(fit5C.ADO, fit6C.ADO, fit6.2C.ADO, fit7C.ADO, rank = TRUE) |
| --- |

| **Model** | **Df** | **Δdf** | **Wald’s test** | **p-value** | **Performance score** |
| --- | --- | --- | --- | --- | --- |
| 5 C | 4757 |  |  |  | 0.00% |
| 6 C.1: with confidence system | 4756 | 1 | 4.45e+29 | <0.001 | 51.59% |
| 6 C.2: with favourable environment | 4756 | 1 | 2.23e+30 | <0.001 | 8.97% |
| 7 C compared to 6C.1 | 4755 | 1 | 2.07e+30 | <0.001 | 100% |
| 7 C compared to 6C.2 | 4755 | 1 | 2.88e+29 | <0.001 |  |

The final model with seven antecedents of vaccine readiness (7 C) is of better performance than a nested 5 C or a nested 6 C model.

## Influence of VR and its antecedent on intention to be vaccinated against HPV

*Data management:*

| **Name** | **Formula** | **Comments** |
| --- | --- | --- |
| HPV_knowledge | IF(connaissance_HPV = 2  AND (mg_parle_t0_v2 = 2 OR mg_parle_t0_v2 = 3)  AND (mg_vaccin_t0_v2 = 2 OR mg_vaccin_t0_v2 = 3)  THEN (HPV_knowledge = 2)  OTHERWISE (HPV_knowledge = 1) | 1 = knowledge of HPV  2 = no knowledge of HPV |
| PAR_intentionality | IF HPV_knowledge = 2  THEN ADO_intentionality = 1  IF Prochaska = 1  AND intention != 1  THEN ADO_intentionality = 2  IF Prochaska = 2  AND intention != 1  THEN ADO_intentionality = 3  IF Prochaska = 3  THEN PAR_intentionality = 5  IF intention = 1  AND Prochaska != 3  THEN PAR_intentionality = 4  IF (statut_vaccinal = 2 OR statut_vaccinal = 3 OR statut_vaccinal = 4)  THEN PAR_intentionality = 6 | 1 = ‘ignorance’  2 = ‘precontemplation’  3 = ‘contemplation’  4 = ‘intention’  5 = ‘preparation’  6 = ‘action’ |
| PAR_acceptance | IF (PAR_intentionality = 1 OR PAR_intentionality = 2)  THEN PAR_acceptance = 1  IF PAR_intentionality = 3  THEN PAR_acceptance = 2  OTHERWISE PAR_acceptance = 3 | 1 = ‘refusal’  2 = ‘deliberation’  3 = ‘acceptance’ |

The statistics were carried out using Jamovi software (The Jamovi Project, 2020), version 2.3 or R Studio, version 3.6, both run under R (R Core Team, 2021), version 4.1, and the libraries Nnet (Ripley & Venables, 2021) and Car (Fox & Weisberg, 2019; fox & Weisberg, 2021).

*Script:*

|  |
| --- |

### MLR with the antecedents of VR

We conducted a multinomial logistic regression on the intention to vaccinate their children against HPV with all the antecedents of VR.

#### MLR unadjusted for sociodemographic variables

| **Model** | **Components** | **Deviance** | **BIC** | **R²_N_** | **Overall model test** | | |
| --- | --- | --- | --- | --- | --- | --- | --- |
|  |  |  |  |  | *χ²* | *dF* | *p-value* |
| 1 | Confidence vaccine  High convenience  Low complacency  High BRB  Coll. Responsibility | 6737 | 6838 | 0.3313 | 2433 | 10 | < .001 |
| 2 | Confidence system  Favourable env. | 6676 | 6811 | 0.3388 | 2493 | 14 | < .001 |

In comparison with the 5 C model, the addition of the two new antecedents significantly improve the overall model.

| **Comparison** | | **Δχ²** | **Δdf** | **p-value** |
| --- | --- | --- | --- | --- |
| *Model* | *Model* |  |  |  |
| 1 | 2 | 60.59 | 4 | < .001 |

Omnibus likelihood ratio tests were performed on each variable.

| **Predictor** | **χ²** | **dF** | **p** |
| --- | --- | --- | --- |
| Confidence vaccine | 4.28 | 2 | 0.118 |
| High convenience | 273.85 | 2 | < .001 |
| Low complacency | 422.40 | 2 | < .001 |
| High BRB | 21.08 | 2 | < .001 |
| Collective responsibility | 33.62 | 2 | < .001 |
| Confidence system | 8.23 | 2 | 0.016 |
| Social conformism | 55.42 | 2 | < .001 |

|  | **Predictor** | **Estimate** | **SE** | **Z** | **p** | **OR** |
| --- | --- | --- | --- | --- | --- | --- |
| Refusal vs. Deliberation | Intercept | -0.590 | 0.065 | -9.016 | < .001 | 0.554 |
|  | Confidence vaccine | 0.059 | 0.059 | 1.004 | 0.315 | 1.061 |
|  | High convenience | -0.061 | 0.047 | -1.303 | 0.193 | 0.941 |
|  | **Low complacency** | **-0.524** | **0.057** | **-9.157** | **< .001** | **0.592** |
|  | High BRB | -0.118 | 0.063 | -1.883 | 0.060 | 0.889 |
|  | **Collective responsibility** | **-0.300** | **0.053** | **-5.679** | **< .001** | **0.741** |
|  | **Confidence system** | **0.127** | **0.050** | **2.566** | **0.010** | **1.136** |
|  | **Social conformism** | **-0.215** | **0.056** | **-3.824** | **< .001** | **0.807** |
| Acceptance vs. Deliberation | Intercept | 0.930 | 0.044 | 21.196 | < .001 | 2.534 |
|  | **Confidence vaccine** | **0.106** | **0.051** | **2.071** | **0.038** | **1.112** |
|  | **High convenience** | **0.599** | **0.043** | **14.039** | **< .001** | **1.821** |
|  | **Low complacency** | **0.638** | **0.055** | **11.687** | **< .001** | **1.893** |
|  | **High BRB** | **0.155** | **0.051** | **3.023** | **0.002** | **1.167** |
|  | **Collective responsibility** | **-0.172** | **0.052** | **-3.287** | **0.001** | **0.842** |
|  | Confidence system | -0.003 | 0.044 | -0.061 | 0.951 | 0.997 |
|  | **Social conformism** | **0.200** | **0.048** | **4.145** | **< .001** | **1.221** |

#### MLR adjusted for sociodemographic variables

| **Model** | **Components** | **Deviance** | **BIC** | **R²_N_** | **Overall model test** | | |
| --- | --- | --- | --- | --- | --- | --- | --- |
|  |  |  |  |  | *χ²* | *dF* | *p-value* |
| 1 | Confidence vaccine  High convenience  Low complacency  High BRB  Coll. Responsibility | 6737 | 6838 | 0.3313 | 2433 | 10 | < .001 |
| 2 | Confidence system  Favourable env. | 6676 | 6811 | 0.3388 | 2493 | 14 | < .001 |
| 3 | Gender  Class | 6512 | 6681 | 0.3591 | 2658 | 18 | < .001 |

In comparison with the 5 C model, the addition of the two new antecedents significantly improve the overall model.

| **Comparison** | | **Δχ²** | **Δdf** | **p-value** |
| --- | --- | --- | --- | --- |
| *Model* | *Model* |  |  |  |
| 1 | 2 | 60.59 | 4 | < .001 |
| 2 | 3 | 164.34 | 4 | < .001 |

Omnibus likelihood ratio tests were performed on each variable.

| **Predictor** | **χ²** | **dF** | **p** |
| --- | --- | --- | --- |
| Confidence vaccine | 5.736 | 2 | 0.057 |
| High convenience | 265.377 | 2 | < .001 |
| Low complacency | 385.116 | 2 | < .001 |
| High BRB | 26.444 | 2 | < .001 |
| Collective responsibility | 30.699 | 2 | < .001 |
| Confidence system | 8.925 | 2 | 0.012 |
| Social conformism | 58.543 | 2 | < .001 |
| Gender | 163.186 | 2 | < .001 |
| Class | 1.054 | 2 | 0.59 |

|  | **Predictor** | **Estimate** | **SE** | **Z** | **p** | **OR** |
| --- | --- | --- | --- | --- | --- | --- |
| Refusal vs. Deliberation | Intercept | -0.874 | 0.099 | -8.821 | < .001 | 0.417 |
|  | Confidence vaccine | 0.042 | 0.058 | 0.728 | 0.467 | 1.043 |
|  | High convenience | -0.069 | 0.047 | -1.472 | 0.141 | 0.933 |
|  | **Low complacency** | **-0.523** | **0.057** | **-9.122** | **< .001** | **0.593** |
|  | **High BRB** | **-0.128** | **0.062** | **-2.056** | **0.04** | **0.880** |
|  | **Collective responsibility** | **-0.274** | **0.053** | **-5.155** | **< .001** | **0.761** |
|  | **Confidence system** | **0.136** | **0.050** | **2.738** | **0.006** | **1.146** |
|  | **Social conformism** | **-0.235** | **0.056** | **-4.173** | **< .001** | **0.790** |
|  | Gender |  |  |  |  |  |
|  | **Boy - Girl** | **0.425** | **0.098** | **4.342** | **< .001** | **1.530** |
|  | classe: |  |  |  |  |  |
|  | 3rd grade - 4th grade | 0.078 | 0.097 | 0.800 | 0.424 | 1.081 |
| Acceptance vs. Deliberation | Intercept | 1.212 | 0.071 | 16.976 | < .001 | 3.361 |
|  | **Confidence vaccine** | **0.124** | **0.052** | **2.390** | **0.017** | **1.132** |
|  | **High convenience** | **0.598** | **0.043** | **13.862** | **< .001** | **1.818** |
|  | **Low complacency** | **0.615** | **0.055** | **11.122** | **< .001** | **1.850** |
|  | **High BRB** | **0.180** | **0.051** | **3.495** | **< .001** | **1.197** |
|  | **Collective responsibility** | **-0.207** | **0.053** | **-3.902** | **< .001** | **0.813** |
|  | Confidence system | 0.002 | 0.044 | 0.049 | 0.961 | 1.002 |
|  | **Social conformism** | **0.200** | **0.049** | **4.112** | **< .001** | **1.222** |
|  | Gender |  |  |  |  |  |
|  | **Boy - Girl** | **-0.781** | **0.087** | **-8.977** | **< .001** | **0.458** |
|  | classe: |  |  |  |  |  |
|  | 3rd grade - 4th grade | 0.079 | 0.086 | 0.918 | 0.359 | 1.082 |

### MLR with VR

We conducted a multinomial logistic regression on the intention to get vaccinated against HPV with VR alone.

#### MLR unadjusted for sociodemographic variables

| **Model** | **Components** | **Deviance** | **BIC** | **R²_N_** | **Overall model test** | | |
| --- | --- | --- | --- | --- | --- | --- | --- |
|  |  |  |  |  | *χ²* | *dF* | *p-value* |
| 1 | Vaccine readiness | 6868 | 6902 | 0.3148 | 2301 | 2 | < .001 |

Omnibus likelihood ratio tests were performed on each variable.

| **Predictor** | **χ²** | **dF** | **p** |
| --- | --- | --- | --- |
| Vaccine readiness score | 2301 | 2 | < .001 |

|  | **Predictor** | **Estimate** | **SE** | **Z** | **p** | **OR** |
| --- | --- | --- | --- | --- | --- | --- |
| Refusal vs. deliberation | Intercept | -0.5914 | 0.06331 | -9.342 | < .001 | 0.5535 |
|  | **Vaccine readiness score** | **-0.894** | **0.05785** | **-15.453** | **< .001** | **0.409** |
| Acceptance vs. deliberation | Intercept | 0.8924 | 0.0424 | 21.046 | < .001 | 2.441 |
|  | **Vaccine readiness score** | **1.3388** | **0.05294** | **25.286** | **< .001** | **3.8143** |

#### MLR adjusted for sociodemographic variables

| **Model** | **Components** | **Deviance** | **BIC** | **R²_N_** | **Overall model test** | | |
| --- | --- | --- | --- | --- | --- | --- | --- |
|  |  |  |  |  | *χ²* | *dF* | *p-value* |
| 1 | Vaccine readiness | 6868 | 6902 | 0.3148 | 2301 | 2 | < .001 |
| 2 | Gender  Class | 6704 | 6771 | 0.3354 | 2466 | 6 | < .001 |

| **Comparison** | | **Δχ²** | **Δdf** | **p-value** |
| --- | --- | --- | --- | --- |
| *Model* | *Model* |  |  |  |
| 1 | 2 | 164.4 | 4 | < .001 |

Omnibus likelihood ratio tests were performed on each variable.

| **Predictor** | **χ²** | **dF** | **p** |
| --- | --- | --- | --- |
| Vaccine readiness score | 2142.185 | 2 | < .001 |
| Gender | 164.040 | 2 | < .001 |
| Class | 0.373 | 2 | 0.83 |

|  | **Predictor** | **Estimate** | **SE** | **Z** | **p** | **OR** |
| --- | --- | --- | --- | --- | --- | --- |
| Refusal vs. deliberation | Intercept | -0.857 | 0.096 | -8.957 | < .001 | 0.425 |
|  | **Vaccine readiness score** | **-0.902** | **0.058** | **-15.449** | **< .001** | **0.406** |
|  | Gender: |  |  |  |  |  |
|  | **Boy – Girl** | **0.444** | **0.096** | **4.617** | **< .001** | **1.558** |
|  | Class: |  |  |  |  |  |
|  | 3e – 4e | 0.036 | 0.096 | 0.373 | 0.709 | 1.036 |
| Acceptance vs. deliberation | Intercept | 1.165 | 0.069 | 16.909 | < .001 | 3.207 |
|  | **Vaccine readiness score** | **1.319** | **0.053** | **24.673** | **< .001** | **3.739** |
|  | Gender: |  |  |  |  |  |
|  | **Boy – Girl** | **-0.746** | **0.085** | **-8.784** | **< .001** | **0.474** |
|  | Class: |  |  |  |  |  |
|  | 3e – 4e | 0.050 | 0.084 | 0.593 | 0.553 | 1.051 |

## References

| Estabrook, R., & Neale, M. (2013). A Comparison of Factor Score Estimation Methods in the Presence of Missing Data: Reliability and an Application to Nicotine Dependence. *Multivariate Behavioral Research*, *48*(1), 1–27. https://doi.org/10.1080/00273171.2012.730072  Fox, J., & Weisberg, S. (2019). *An R companion to applied regression* (Third edition). SAGE.  fox, J., & Weisberg, S. (2021). *car: Companion to Applied Regression* (3.0-12) [R]. https://CRAN.R-project.org/package=car  Hooper, D., Coughlan, J., & Mullen, M. R. (2008). Structural Equation Modelling: Guidelines for Determining Model Fit. *Electronic Journal of Business Research Methods*, *6*(1), 53–60.  Hoyle, R. H. (Ed.). (2012). *Handbook of structural equation modeling*. Guilford Press.  Hu, L., & Bentler, P. M. (1999). Cutoff criteria for fit indexes in covariance structure analysis: Conventional criteria versus new alternatives. *Structural Equation Modeling: A Multidisciplinary Journal*, *6*(1), 1–55. https://doi.org/10.1080/10705519909540118  Lenth, R. V., Buerkner, P., Herve, M., Love, J., Miguez, F., Riebl, H., & Singmann, H. (2022). *emMeans: Estimated Marginal Means, aka Least-Squares Means* (1.7.4-1). https://CRAN.R-project.org/package=emmeans  Lüdecke, D., Ben-Shachar, M., Patil, I., Waggoner, P., & Makowski, D. (2021). performance: An R Package for Assessment, Comparison and Testing of Statistical Models. *Journal of Open Source Software*, *6*(60), 3139. https://doi.org/10.21105/joss.03139  Oudin Doglioni, D., Pham-Hung D’Alexandry D’Orengiani, A.-L., Galactéros, F., & Gay, M.-C. (2021). Psychometric characteristics of the Revised Illness Perception Questionnaire (IPQ-R) in adults with sickle cell disease. *Health Psychology and Behavioral Medicine*, *10*(1), 60–80. https://doi.org/10.1080/21642850.2021.2016411  R Core Team. (2021). *R: A Language and environment for statistical computing.* (4.1).  Revelle, W. (2022). *Psych: Procedures for Psychological, Psychometric, and Personality Research* (2.2.5) [R package]. Northwestern University. https://CRAN.R-project.org/package=psych  Ripley, B., & Venables, W. (2021). *nnet: Feed-Forward Neural Networks and Multinomial Log-Linear Models* (7.3-17) [R]. https://cran.r-project.org/web/packages/nnet/index.html  Rosseel, Y. (2012). **lavaan**: An *R* Package for Structural Equation Modeling. *Journal of Statistical Software*, *48*(2). https://doi.org/10.18637/jss.v048.i02  Rosseel, Y., & Jorgensen, T. D. (2019). *Package ‘lavaan’* (0.6-10) [R]. https://cran.r-project.org/web/packages/lavaan/lavaan.pdf  Steiger, J. H. (2002). When constraints interact: A caution about reference variables, identification constraints, and scale dependencies in structural equation modeling. *Psychological Methods*, *7*(2), 210–227. https://doi.org/10.1037/1082-989X.7.2.210  Steiger, J. H. (2007). Understanding the limitations of global fit assessment in structural equation modeling. *Personality and Individual Differences*, *42*(5), 893–898. https://doi.org/10.1016/j.paid.2006.09.017  The Jamovi Project. (2020). *Jamovi* (1.2).  Vuong, Q. H. (1989). Likelihood Ratio Tests for Model Selection and Non-Nested Hypotheses. *Econometrica*, *57*(2), 307. https://doi.org/10.2307/1912557  Wheaton, B., Muthen, B., Alwin, D. F., & Summers, G. F. (1977). Assessing Reliability and Stability in Panel Models. *Sociological Methodology*, *8*, 84–136. https://doi.org/10.2307/270754 |
| --- |

# Supplemental material 5: MLR resultat synthesis

**Coefficients (β) estimated in unadjusted and adjusted multinomial logistic regression models.**

|  |  | HCW | | | Parents | | | Adolescents | | |
| --- | --- | --- | --- | --- | --- | --- | --- | --- | --- | --- |
|  |  | unadjusted | | | unadjusted | | | unadjusted | | |
|  |  | Estimate | 95% CI | | Estimate | 95% CI | | Estimate | 95% CI | |
|  |  |  | Lower | Upper |  | Lower | Upper |  | Lower | Upper |
| Refusal | Confidence vaccine | -0.370 | -0.495 | -0.244 | 0.187 | -0.116 | 0.490 | 0.059 | -0.056 | 0.173 |
|  | Convenience | -0.062 | -0.160 | 0.035 | -0.014 | -0.251 | 0.224 | -0.061 | -0.152 | 0.031 |
|  | Complacency | -0.136 | -0.209 | -0.064 | -0.164 | -0.415 | 0.088 | -0.524 | -0.636 | -0.412 |
|  | Calculation | -0.476 | -0.595 | -0.357 | 0.149 | -0.098 | 0.397 | -0.118 | -0.241 | 0.005 |
|  | Collective responsibility | -0.499 | -0.616 | -0.383 | -0.175 | -0.345 | -0.004 | -0.300 | -0.404 | -0.197 |
|  | Confidence system | -0.154 | -0.240 | -0.068 | 0.048 | -0.162 | 0.258 | 0.127 | 0.030 | 0.224 |
|  | Social conformism | -0.341 | -0.450 | -0.231 | -0.073 | -0.302 | 0.157 | -0.215 | -0.325 | -0.105 |
|  | Vaccine readiness | -1.685 | -1.839 | -1.531 | -0.156 | -0.385 | 0.073 | -0.894 | -1.007 | -0.781 |
| Acceptance | Confidence vaccine | 0.906 | 0.790 | 1.021 | 0.180 | -0.037 | 0.397 | 0.106 | 0.006 | 0.207 |
|  | Convenience | 0.031 | -0.076 | 0.137 | 0.731 | 0.512 | 0.949 | 0.599 | 0.516 | 0.683 |
|  | Complacency | 0.081 | -0.005 | 0.167 | 1.185 | 0.947 | 1.423 | 0.638 | 0.531 | 0.745 |
|  | Calculation | 0.946 | 0.827 | 1.065 | 0.428 | 0.244 | 0.612 | 0.155 | 0.054 | 0.255 |
|  | Collective responsibility | 0.522 | 0.380 | 0.665 | -0.041 | -0.197 | 0.114 | -0.172 | -0.275 | -0.070 |
|  | Confidence system | 0.198 | 0.121 | 0.275 | 0.039 | -0.139 | 0.217 | -0.003 | -0.089 | 0.083 |
|  | Social conformism | 0.579 | 0.478 | 0.681 | 0.352 | 0.163 | 0.541 | 0.200 | 0.105 | 0.295 |
|  | Vaccine readiness | 2.933 | 2.750 | 3.115 | 2.408 | 2.167 | 2.649 | 1.339 | 1.235 | 1.443 |
|  |  |  |  |  |  |  |  |  |  |  |
|  |  | adjusted for sociodemographic data | | | adjusted for sociodemographic data | | | adjusted for sociodemographic data | | |
|  |  | Estimate | 95% CI | | Estimate | 95% CI | | Estimate | 95% CI | |
|  |  |  | Lower | Upper |  | Lower | Upper |  | Lower | Upper |
| Refusal | Confidence vaccine | -0.421 | -0.571 | -0.270 | 0.211 | -0.098 | 0.519 | 0.042 | -0.071 | 0.156 |
|  | Convenience | -0.078 | -0.194 | 0.037 | 0.018 | -0.227 | 0.262 | -0.069 | -0.161 | 0.023 |
|  | Complacency | -0.111 | -0.195 | -0.026 | -0.157 | -0.412 | 0.099 | -0.523 | -0.635 | -0.411 |
|  | Calculation | -0.436 | -0.578 | -0.295 | 0.189 | -0.064 | 0.442 | -0.128 | -0.250 | -0.006 |
|  | Collective responsibility | -0.616 | -0.758 | -0.475 | -0.169 | -0.342 | 0.004 | -0.274 | -0.378 | -0.170 |
|  | Confidence system | -0.136 | -0.239 | -0.032 | 0.019 | -0.195 | 0.234 | 0.136 | 0.039 | 0.234 |
|  | Social conformism | -0.364 | -0.496 | -0.233 | -0.081 | -0.316 | 0.153 | -0.235 | -0.346 | -0.125 |
|  | Vaccine readiness | 2.933 | 2.750 | 3.115 | -0.087 | -0.328 | 0.153 | -0.902 | -1.017 | -0.788 |
| Acceptance | Confidence vaccine | 0.863 | 0.722 | 1.004 | 0.186 | -0.034 | 0.407 | 0.124 | 0.022 | 0.225 |
|  | Convenience | -0.032 | -0.157 | 0.092 | 0.773 | 0.551 | 0.996 | 0.598 | 0.513 | 0.682 |
|  | Complacency | 0.054 | -0.045 | 0.153 | 1.176 | 0.936 | 1.415 | 0.615 | 0.507 | 0.724 |
|  | Calculation | 0.963 | 0.823 | 1.103 | 0.486 | 0.296 | 0.675 | 0.180 | 0.079 | 0.281 |
|  | Collective responsibility | 0.556 | 0.388 | 0.723 | -0.008 | -0.167 | 0.150 | -0.207 | -0.312 | -0.103 |
|  | Confidence system | 0.174 | 0.082 | 0.265 | 0.027 | -0.155 | 0.208 | 0.002 | -0.085 | 0.089 |
|  | Social conformism | 0.546 | 0.424 | 0.667 | 0.360 | 0.167 | 0.553 | 0.200 | 0.105 | 0.296 |
|  | Vaccine readiness | 2.817 | 2.600 | 3.034 | 2.526 | 2.271 | 2.780 | 1.319 | 1.214 | 1.424 |
|  | Ajustement on: | Gender |  |  | Gender |  |  | Gender |  |  |
|  |  | Age |  |  | Age |  |  | class |  |  |
|  |  | Profession |  |  | Profession |  |  |  |  |  |
